# Continuous optical–electrical field diagnostics separate environmental modulation from irreversible perovskite solar-cell failure

**DOI:** 10.1039/d6ee05477c

**Published:** 2026-10-06

**Authors:** Jaume Noguera-Gómez, Rafael Abargues, Pablo P. Boix, Sandy Sánchez-Alonso

**Affiliations:** a School of Physical and Mathematical Sciences, Nanyang Technological University 637371 Singapore; b Instituto de Ciencia de los Materiales de la Universitat de València (ICMUV) Valencia Spain; c Instituto de Tecnología Química (ITQ, UPV-CSIC) Valencia Spain; d School of Engineering and Architecture of Fribourg, HES-SO Fribourg Switzerland sandy.sanchez@hefr.ch; e Laboratory of Photonics and Interfaces, École Polytechnique Fédérale de Lausanne Lausanne Switzerland

## Abstract

Outdoor photovoltaic records confound reversible weather response with irreversible degradation. Here, we establish a continuous optical–electrical field-diagnostic framework that separates shared environmental modulation from persistent cell-specific failure and tests optical timing, transferability and predictive value. We monitored encapsulated perovskite cells in Alpine and Mediterranean climates using synchronised electrical, spectral, thermal, humidity and passive RGB measurements. A fixed C1/C3 peer reference removed the dominant cohort-wide response and exposed four persistent Alpine terminal failures; seasonal recovery of both reference cells showed that the deep winter minimum was predominantly reversible. An analysis-locked retrospective colour replay matched three of four retrospective terminal markers (optical-first offsets of 1, 4 and 6 days), and a locked marker-definition audit then resolved the timing. With an earlier daily-persistent marker, C2 became coincident, C8 electrical-first, and the only optical-first change, at C5/C6, was shared with non-failing C3/C4. The Alpine record therefore establishes day-scale optical–electrical co-evolution around failure, and the same-date C3/C4 control shows that this optical-first change is not device-specific. At Paterna, optical changes occurred 18 and 13 days after electrical onset and did not reproduce the Alpine timing. A date-disjoint hidden test established reliable passive measurement of wetness and thaw, which did not improve three-day health prediction. Endpoint diffraction distinguished PbI_2_-rich photocurrent-loss states from the lower-ratio mixed voltage–current failure. Over the common winter window, Alpine survivors recovered while Paterna C3/C4 declined despite overlapping per-cell integrated yields. The framework separates environmental response, irreversible failure, optical timing and cross-climate transfer within a single field-diagnostic workflow.

## Abstract 2

Continuous optical-electrical monitoring separates reversible weather modulation from cell-specific perovskite failure. Optical timing depends on the electrical marker, and no device-specific advance warning is established at either site.

## Graphical abstract

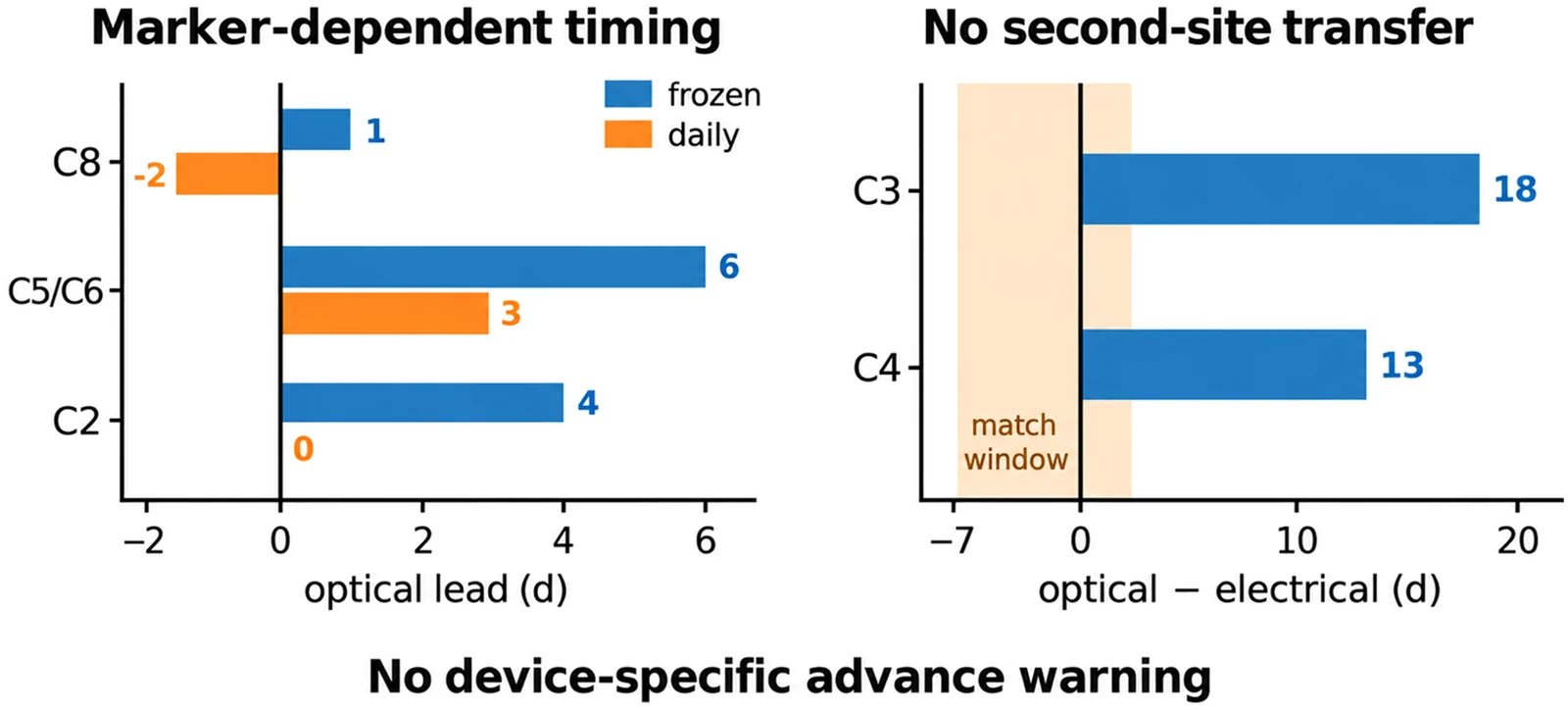

**Broader context**

Scaling emerging photovoltaics requires not only high efficiency, but a credible understanding of how devices fail under real weather. This is particularly important for perovskite solar cells, where reversible responses to temperature, irradiance and moisture can mask the onset of irreversible degradation during outdoor operation. Here, we establish a continuous field-diagnostic framework that combines cell-level electrical measurements, optical imaging, device temperature, irradiance and meteorology to separate the dominant common-mode environmental response from persistent, cell-specific degradation. This transforms outdoor stability testing from endpoint lifetime ranking into event-resolved monitoring. It tests whether optical changes precede electrical failure rather than assuming they do. Here, colour changes tracked the same day-scale failure period in Alpine and followed electrical onset at Paterna, so no device-specific advance signal was demonstrated at either site. The second-site replay also shows when a diagnostic signature does not transfer across contrasting climates. By resolving degradation against the environmental background in which it occurs, the approach provides a route towards climate-aware reliability metrics and more informative accelerated tests. More broadly, it offers a general strategy for qualifying emerging energy technologies under complex, non-stationary operating conditions, helping bridge the gap between laboratory durability claims and dependable field performance.

## 1. Introduction

Perovskite solar cells have reached high power-conversion efficiencies, yet operational lifetime and long-term reliability remain major barriers to widespread deployment. A central field question is whether irreversible degradation can be distinguished from reversible performance fluctuations under different climates before substantial output is lost. This is especially relevant when comparing cold, high-irradiance, snow-prone Alpine sites with warm, humid Mediterranean sites close to major photovoltaic demand. Laboratory tests isolate individual stresses and compare device designs. Outdoor operation instead combines changes in irradiance and spectrum, temperature, moisture, oxygen, ultraviolet exposure, mechanical and thermal cycling, electrical bias and dark recovery. No single constant-stress test reproduces this sequence. International Summit on Organic Photovoltaic Stability (ISOS) procedures and later perovskite-specific guidance have improved reporting consistency, and field campaigns now provide realistic energy-yield and lifetime baselines.^1–14^ However, an electrical trace alone cannot show whether a winter loss is temporary or whether one cell has begun to fail. The practical task is therefore to separate weather-driven performance changes from persistent damage.

Outdoor records make this separation difficult. Perovskite cells and minimodules respond strongly to temperature and irradiance, behave differently from crystalline silicon at low light, and can recover during darkness or between seasons.^15,16^ A long record may therefore contain winter minima, recovery periods and architecture-dependent ageing that are poorly represented by a single *T*_80_ value. Continuous illumination is not necessarily a conservative substitute for natural operation because dark intervals can restore metastable states or expose different degradation pathways.^10,11^ Accelerated tests reproduce field ageing more reliably when they explicitly represent the relevant temperature, illumination and interface-dependent processes.^13,14,17^

This electrical ambiguity has a physical basis. Composition, crystal structure, processing history and defects define the thermodynamic and kinetic landscape of metal-halide perovskites.^18–20^ Current–voltage hysteresis, scan-history dependence and time-dependent maximum-power output arise from coupled electronic and ionic processes, including mobile defects, interfacial charge accumulation and field-dependent redistribution.^21–26^ Reversible light-induced defect formation, halide redistribution and phase segregation can change current, voltage, colour and luminescence without permanent absorber loss.^27–31^ Their magnitude and recovery time depend on illumination history, architecture, composition and temperature. Raw power or efficiency is therefore not a direct measure of cell health. Weather can move several devices together, while an early cell-specific loss remains smaller than this shared variation.

Irreversible degradation also follows several coupled routes. Oxygen and illumination can drive superoxide-mediated reactions. Trapped charge can accelerate moisture-assisted decomposition, heat can destabilise organic and hybrid perovskites, and ultraviolet exposure can activate photocatalytic and interfacial reactions.^20,32–40^ Mobile halides, iodine-containing volatile products and metal species can redistribute across contacts. Thermal mismatch, imperfect adhesion and encapsulant mechanics can promote cracking or delamination.^2,3,17,41–46^ These pathways often coexist and become spatially non-uniform near edges, contacts and local barrier defects. Thermal decomposition and rapid non-equilibrium processing can also leave spatial fingerprints in the film.^47–50^ Post-mortem diffraction and microscopy constrain the recovered material state, but they cannot reconstruct the field chronology unless sample identity, recovery, storage and unblinding are controlled.^17,41^

Materials and device engineering have substantially extended operational stability. Flash infrared annealing (FIRA) removes the antisolvent step and reduces absorber crystallisation from minutes to seconds while retaining control over nucleation, growth and thermal history. Early planar and mesoscopic studies established efficient devices, rapid manufacturing and lower processing burden, and pulse-time studies resolved the corresponding nucleation and growth regimes.^51–54^ For FAPbI_3_-rich absorbers, thermally controlled growth, multication screening and solution-crystallisation studies connect heating rate and composition to phase selection and durability.^18,55–59^ TEMPO bulk passivation has extended this route to rapid-annealed FAPI devices above 20% efficiency with multi-thousand-hour operational and thermal retention.^60^ Surface passivation, low-defect interfaces, two-dimensional/three-dimensional heterojunctions, ion-blocking contacts, ultraviolet-tolerant transport layers and robust encapsulation have also produced major gains under controlled and outdoor-relevant tests.^4,36,43,44,61–74^ As one layer becomes more stable, the dominant failure mode can shift to another interface, an electrode or the package. Field diagnostics must therefore distinguish the operating environment, reversible device response and irreversible endpoint instead of combining them in one efficiency curve.

This separation is also a measurement and validation problem. Reliable characterisation requires stabilised output or maximum-power tracking, explicit scan protocols, temperature and irradiance metadata, and transparent treatment of censored records, missing observations and measurement history.^1,12,21,75^ FAIR databases and large-scale analyses have exposed the heterogeneity of stability reporting and identified population-level relationships between efficiency, architecture and durability.^76–78^ Outdoor platforms now combine parallel maximum-power tracking, current–voltage scans and environmental sensing. Machine-learning (ML) methods can describe diurnal and long-term behaviour or forecast performance within a defined training domain.^79–81^ Because neighbouring observations in a field record are strongly related, prediction tests must keep complete time blocks apart between training and evaluation. Preprocessing, event definitions and negative controls must also be fixed before testing, followed by replay on an independent dataset.

Optical records offer an underused source of information because degradation products, wet surfaces, obstructions, contact-edge changes and process-induced spatial patterns can often be visibly or spectrally distinct.^48^ Field images are nevertheless affected by illumination colour, glare, snow, water, shadows, camera-state changes and missing observations. Outdoor studies have mainly focused on energy yield, temperature coefficients, seasonal performance, lifetime and post-exposure analysis. Longer ML records and sunlight-excited outdoor photoluminescence imaging now show the value of spatially resolved diagnostics.^5–15,17,41,79,82^ Photoluminescence and implied-voltage imaging reveal device physics that colour cannot, but they require dedicated optics, filtering and acquisition hardware at each site. Passive RGB imaging uses commodity cameras, no active excitation and low-compute analysis, at the cost of specificity. A port-referenced colour index can therefore support continuous window-level surveillance across a larger fleet. Its value must still be tested explicitly: the changepoint specification must be locked before replay and its provenance stated, the matched fraction, changepoint exposure and timing against an early electrical marker must be reported, a same-date control in non-failing windows must be used to test device specificity, and any camera-derived environmental state must be distinguished from actual degradation prediction.

Here, we monitor matched sets of encapsulated perovskite cells in Alpine and Mediterranean climates using synchronised electrical, spectral, environmental and passive RGB measurements. A fixed peer reference built retrospectively from the two full-campaign Alpine electrical survivors removes the dominant shared response and exposes persistent cell-specific divergence; raw autumn–winter–spring recovery of those controls provides an independent check that the deep winter common mode is predominantly reversible. We then quantify the in-sample timing and specificity of an analysis-locked retrospective colour replay, validate camera-derived wetness and thaw measurements, and assess whether they improve three-day health prediction. A Paterna replay locked before formal electrical-onset unblinding tests whether the Alpine optical–electrical timing transfers to a second climate. Cell-resolved endpoint XRD compares the recovered absorber states with the electrical failure type. Finally, an eight-position string benchmark contrasts two retrospective switching calendars. The analysis keeps measurement validity, prediction, transfer, endpoint interpretation and engineering benchmark value as separate questions.

## 2. Results

### 2.1. Two climates, one matched device design

Eight independently contacted Alpine cells were distributed across four two-cell FTO-glass substrates. The Alpine and Paterna cohorts were fabricated in the same batch and used the same n–i–p architecture: glass/FTO/compact TiO_2_/mesoporous TiO_2_/(FA_0.95_Cs_0.05_)PbI_3_/PEAI/Spiro-OMeTAD/Au. Fig. 1a shows the common device and encapsulation design. The schematic identifies the functional layers and the outer thin-glass cover with its perimeter seal. The cross-sectional SEM shows the inner conformal parylene/TiO_2_ barrier. Keeping the fabrication batch, device stack and package fixed across the two cohorts holds device preparation constant for the subsequent field comparison. The full-size SEM images, with their acquisition scale bars, are reproduced in Fig. S1a, and the shared architecture is drawn in Fig. S1b.

**Fig. 1:**
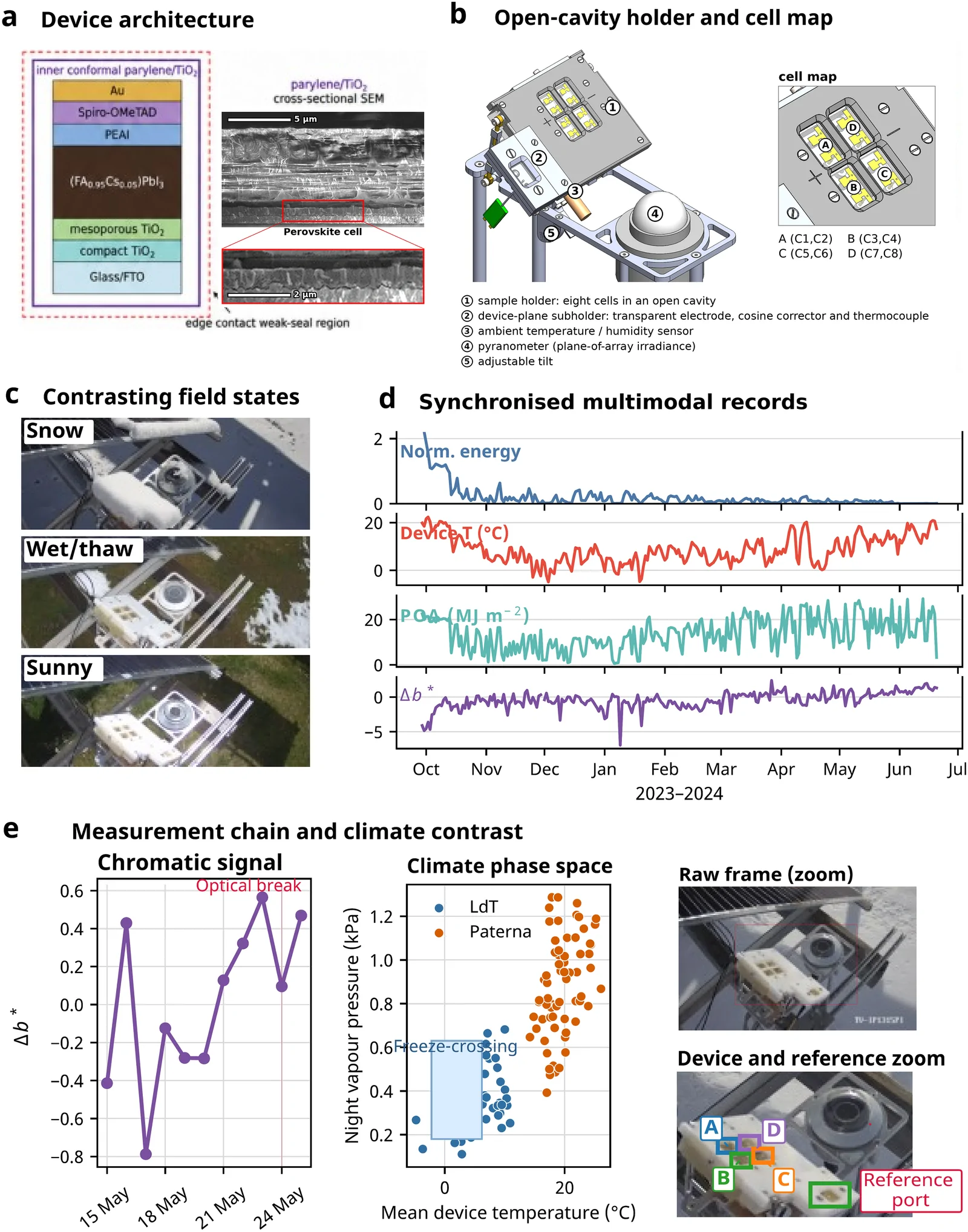
Outdoor platform and measurement chain. (a) Device stack, dual encapsulation and cross-sectional SEM. (b) Open-cavity holder, co-planar irradiance/environment sensors, reference-cell Pt probe and A–D/C1–C8 map. (c) Snow, wet/thaw and dry-sunny camera states. (d) Synchronised electrical, thermal, irradiance and colour records. (e) Port-referenced Δb* example, site climate phase space, raw frame and ROI/reference-port overlay.

Fig. 1b shows the open-cavity holder, sensor and probe geometry, and permanent sample map. The holder maintained the electrical contacts while exposing the active surfaces to outdoor conditions. Irradiance, ambient temperature and relative humidity were measured next to the device plane; a Pt probe on the reference-cell back contact provided a local film-temperature measurement. The inset maps substrates A–D to cells C1–C8 and links each physical position to its electrical channel and optical region. The adjustable tilt kept the cells and incident-radiation sensors in a common plane. The matched Paterna set used the same device design and synchronised electrical, spectral and environmental acquisition. Both platforms also carried a fixed RGB camera. The Alpine archive was used for the main optical analysis, whereas the Paterna archive remained unexamined until the replay locked before formal electrical-onset unblinding in Section 2.5. The public project webpage documents both installations and their remote acquisition system.^83^

Fig. 1c illustrates three common visual states: snow cover, wet or actively thawing surfaces, and dry sunny exposure. These states produce large changes in obstruction, illumination and surface condition. Fig. 1d places representative electrical, thermal, irradiance and colour records on the same timeline. The traces show strong seasonal changes in normalised electrical output and their relation to device temperature and plane-of-array irradiance. They also show additional structure in the port-referenced Δb* colour signal.

Fig. 1e defines the optical measurement. Each image was registered to fixed device regions of interest and normalised against a fixed reference port. Port-referenced Δb* is the yellowness of a cell region minus the yellowness of the reference port. Illumination changes shared by both regions therefore largely cancel. An increase in Δb* indicates visible yellowing of the cell area, which can accompany PbI_2_-rich or δ-phase decomposition products. The camera does not identify phase directly. The climate plot in the same panel separates the colder Alpine regime, including repeated freeze crossings, from the warmer Paterna regime with higher night-time vapour pressure. Sections S1–S3 describe the workflow, camera archive and annotation labels. Fig. S2 and S2b show both deployments, the station hardware and the complete monitoring chain.

### 2.2. Reversible dips separate from terminal loss

Winter conditions changed raw responsivity several-fold as device temperature approached or crossed 0 °C and plane-of-array irradiance varied. Responsivity was calculated as *J*_sc_ divided by plane-of-array irradiance. For the retrospective health construction, C1 and C3, the two cells that remained electrically active through the final powered record, were used as a fixed same-day peer reference. For C2, C4, C5, C7 and C8 the denominator was the same-day median of C1 and C3; C1 and C3 used leave-self-out referencing to the other control. Thus C4 and the terminal cells never entered the denominator, and the reference composition did not change as cells failed. Because C1 and C3 were identified from their full-campaign survival, this is a retrospective diagnostic construction rather than a prospectively designated control rule.

This fixed reference removes the dominant cohort-wide motion and exposes persistent cell-specific divergence, but any degradation shared by C1 and C3 cancels. Their absolute plane-of-array-normalised responsivity therefore provides an independent reversibility check. Using the same seasonal windows as Fig. 2d, the spring medians of C1 and C3 reached 107% and 109% of their autumn medians after pronounced winter suppression. The cohort, including C1 and C8, also lost most raw responsivity in October 2023; that early step did not recover, and the 5 October–5 November autumn window straddles it. The ratios therefore describe seasonal rebound, not reversibility of all common loss. C8, which later failed, showed the same winter rebound before its late-May collapse, so the rebound is not an artefact of selecting C1 and C3 on full-campaign survival. The dominant winter common mode is reversible in these controls, although a smaller irreversible component shared by both cannot be excluded. Section S4 reports the absolute-control and reference-sensitivity analyses.

**Fig. 2:**
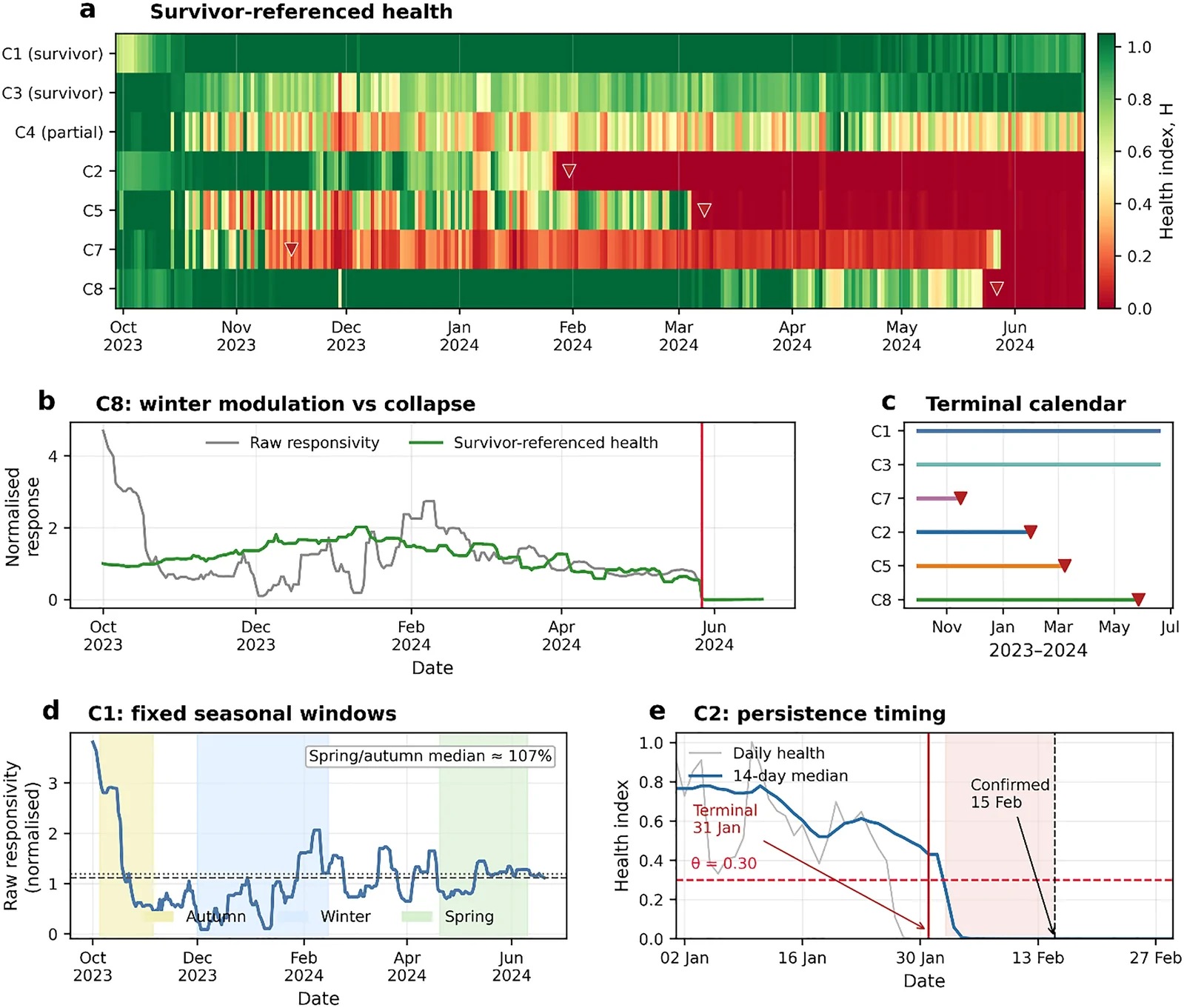
Fixed C1/C3 peer referencing separates dominant reversible common-mode modulation from persistent cell-specific terminal loss. (a) Daily health for the seven Alpine cells retained in the peer-referenced analysis; C6 was excluded after becoming electrically unusable on 2 October 2023. (b) C8 raw and peer-referenced responsivity, showing the winter rebound that precedes its distinct late-May collapse. (c) Retrospective terminal-marker calendar; C1 and C3 are right-censored. (d) C1 fixed-window autumn–winter–spring comparison; the autumn window straddles the early October cohort-wide step, and the corresponding C3 recovery is reported in Table S4a. (e) C2 daily health and 14 day median, showing the frozen retrospective marker (31 January), first *θ* = 0.30 crossing (2 February) and persistence confirmation (15 February; Table S4d).

Fig. 2a shows the health trajectories of the seven Alpine cells retained for peer-referenced analysis. C6 became electrically unusable on 2 October 2023 and was therefore excluded from the peer-referenced health and terminal-event analyses; its cell identity was retained for the optical/XRD mapping and it was handled separately in the retrospective string reconstruction. C1 and C3 were still operating when the station stopped and are treated as right-censored survivors. C4 developed a persistent partial loss. C7, C2, C5 and C8 reached terminal endpoints. Fig. 2b illustrates the separation for C8. Its raw responsivity follows the strong winter decline of the batch, whereas the fixed C1/C3 reference removes most of that shared trend and retains the abrupt late-May collapse. Fig. 2c summarises the retrospective terminal outcomes. Sensitivity analyses using C1 alone, C3 alone, a leave-one-out peer median and raw responsivity preserved the orders-of-magnitude terminal collapse of C2, C5 and C8 and the weaker mixed-mode C7 phenotype (Section S4).

Fig. 2d shows the same fixed-window comparison for C1: a decline through winter and a return to approximately its autumn level in spring; the corresponding C3 recovery is reported in Table S4a. A large seasonal decrease in raw output is therefore not sufficient evidence of irreversible failure. A *T*_80_-type threshold applied directly to raw output would have classified this reversible winter minimum as failure. Fig. 2e shows the C2 chronology. The frozen retrospective terminal marker is 31 January; the 14 day median first crosses the fixed *θ* = 0.30 threshold on 2 February. The 14-recorded-day persistence requirement is met on 15 February (Table S4d). The retrospective terminal calendar was held fixed during the final Alpine optical replay. Rule-derived 7 day and 14 day rolling-median markers, each crossing *H* < 0.30 with 14 recorded days of persistence, preserved the same 3/4 optical classification and the same four other device-window changepoints without refitting the detector (Table S4c).

### 2.3. Optical timing depends on the electrical marker

The analysis-locked retrospective colour replay matched three of the four retrospective terminal markers (Fig. 3a). It preceded those markers by 4, 6 and 1 days in C2, C5 and C8, respectively. The median optical-first offset was 4 days, and the matched fraction was 3/4 (exact 95% interval 19–99%). Four other device-window changepoints occurred over 2.90 monitored window-years. This corresponds to 1.38 per window-year (exact 95% interval 0.38–3.53) and a matched fraction of 0.43. Each matched changepoint identified the two-cell substrate containing the later electrical failure. C7 was missed during its low-illumination winter onset, which defines the present correspondence limit.

**Fig. 3:**
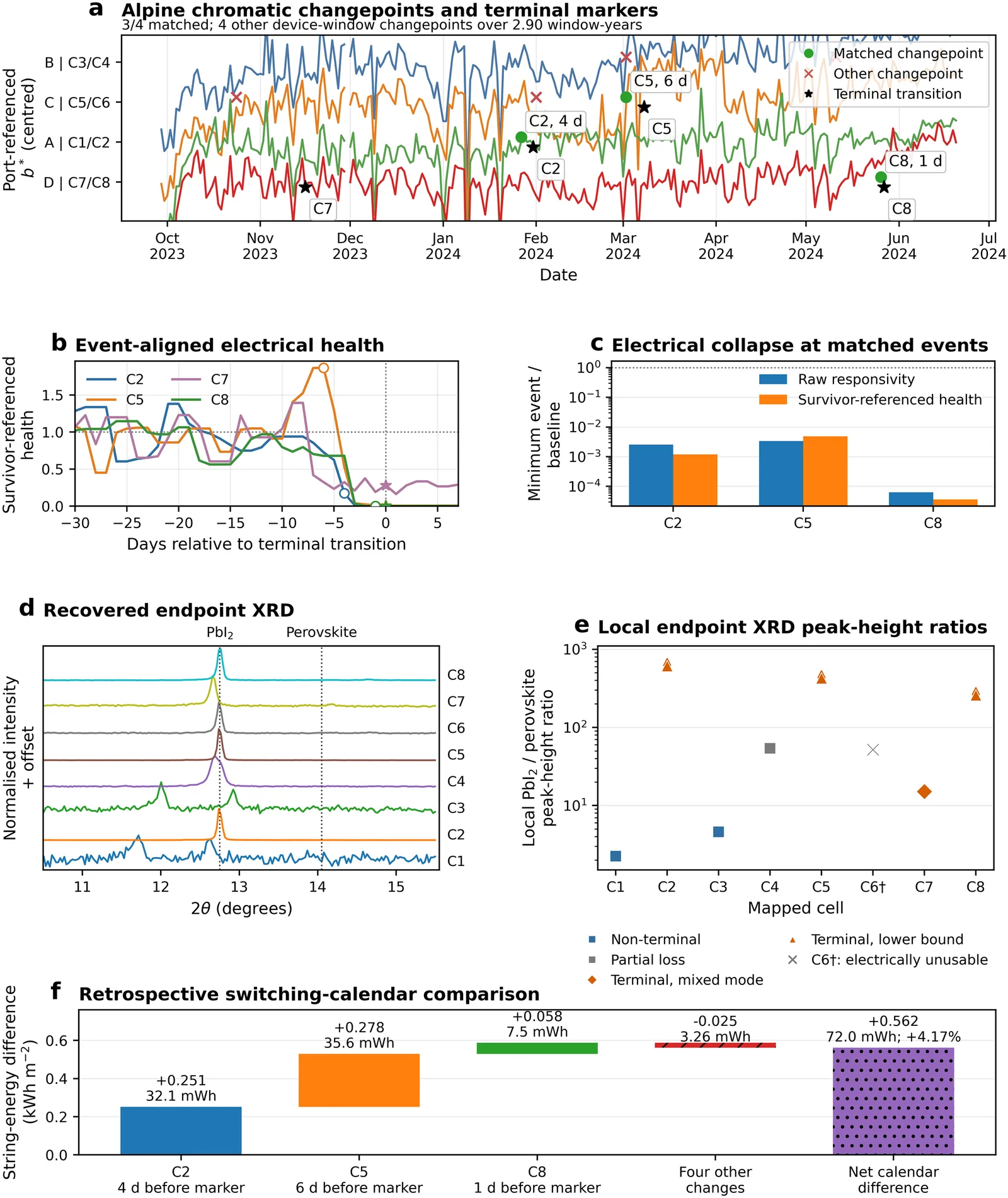
Alpine optical changepoints, terminal loss, endpoint XRD and retrospective switching benchmark. (a), Analysis-locked retrospective port-referenced b* changepoints against the frozen terminal markers: 3/4 matched, median optical-first offset 4 days, and four other device-window changepoints over 2.90 window-years (1.38 per window-year). Against the earlier daily-persistent electrical marker the same changepoints give 1/4 (Table S4c). (b) Terminal-aligned survivor-referenced health. (c) Electrical loss from changepoint to terminal date. (d) Cell-resolved recovered endpoint XRD patterns under the confirmed map; the raw diffraction patterns are included in the SI and Code package. (e) PbI_2_/perovskite peak-height ratios by field outcome; C6† denotes the electrically unusable cell with no frozen terminal transition, and upward markers identify lower-bound ratios where the perovskite reflection approached the local background. (f) Retrospective switching-calendar benchmark: 0.588 kWh m^−2^ gross and 0.562 kWh m^−2^ net after the 0.025 kWh m^−2^ cost of the four unmatched changepoints, a net relative difference of 4.17% on the terminal-date campaign energy. The benchmark compares two retrospective switching calendars and is not incremental energy attributed to advance warning.

With four terminal events, these metrics describe the Alpine cohort, not a population-level correspondence rate. The 3/4 fraction measures correspondence to the retrospective terminal calendar, not electrical precedence. Using the deposited daily health series and an alternative unsmoothed daily-persistent marker—the first recorded day with *H* < 0.30 followed by 14 consecutive recorded days below 0.30—leaves the optical changepoints unchanged. Under this definition, C2 is coincident, C8 is electrical-first by 2 days, C5/C6 is optical-first by 3 days, and C7 remains missed. Under the historical 1–14 d matching rule, the correspondence falls from 3/4 to 1/4 (Table S4c). The non-failing C3/C4 window also changed on 2 March, the same date as C5/C6, and this synchrony persisted across all 15 tested combinations of PELT penalty 4–12 and minimum segment length 7–14 d. Thus, the only optical-first change is shared with a non-failing window, and this four-event Alpine record does not establish a device-specific advance optical precursor.

Fig. 3b aligns all four terminal events to their failure dates. In C2, C5 and C8, survivor-referenced health was still measurable at the changepoint and then fell towards the terminal endpoint. C7 is the missed-event control. Fig. 3c shows that raw responsivity and survivor-referenced health decreased by several orders of magnitude between changepoint and failure. Photocurrent loss dominated these transitions, while *V*_oc_ and fill factor changed less. This pattern is consistent with progressive loss of electrically active area. The field measurements do not resolve the microscopic cause. Raw C5 responsivity stays within its preceding winter range until 4 March and collapses on 5 March, the same date as the daily-persistent peer-referenced marker, so the 2 March optical change is temporally earlier but is not created by the C1/C3 denominator.

Endpoint XRD distinguished the recovered absorber states under the confirmed cell-resolved map (Fig. 3d and e; Section S5, Tables S5a and S5b, and Fig. S5 and S5b). C1 and C3 retained the strongest perovskite reflections and the lowest PbI_2_/perovskite ratios. C2, C5 and C8 were strongly PbI_2_-rich. Their ratios are reported as lower bounds because the remaining perovskite peak approached the local background. C4 and C6 were intermediate. C6 is included in this endpoint comparison because XRD was performed on the recovered physical cells independently of its exclusion from the survivor-referenced electrical analysis. C7 retained a clear perovskite reflection after mixed-mode terminal failure, giving a lower finite PbI_2_/perovskite ratio than the PbI_2_-rich photocurrent-loss endpoints and a distinct recovered state. C7 also had the longest terminal-to-recovery interval (228 days *versus* 152, 115 and 35 days for C2, C5 and C8), so this contrast does not by itself separate field degradation from storage and preparation effects. Endpoint XRD therefore describes the recovered state through descriptive local peak-height ratios; it does not provide phase fractions or reconstruct the initiating field sequence.

Fig. 3f compares two retrospective switching calendars. In the reconstructed eight-position string, bypassing at the optical dates rather than the frozen terminal dates changes energy by 0.251, 0.278 and 0.058 kWh m^−2^ for C2, C5 and C8, respectively. The gross difference is 0.588 kWh m^−2^ over the nominal eight-position active area. The four unmatched changepoints cost 0.025 kWh m^−2^ (3.26 mWh), leaving a net difference of 0.562 kWh m^−2^ (72.0 mWh). This is 4.17% of the campaign energy delivered under terminal-date bypass. The estimate assumes prompt, low-loss switching and applies only to the tested active area. Because the earlier-marker audit makes C2 coincident and C8 electrical-first, and the C5/C6 optical change is not device-specific, this is an engineering benchmark between two retrospective switching calendars, not energy attributed to advance camera warning. Section S6, Table S6 and Fig. S6a–c report the event-level energy and sensitivity analyses; Section S5 and Fig. S5 and S5b report the endpoint mapping.

### 2.4. The camera measures wetness but does not improve prediction

Two frozen annotation campaigns converted the RGB archive into defined environmental states. The first assigned five observation-context labels to 135 environment-valid images across 82 date or episode groups and was later audited on a separate frozen 52-image set. The second assigned a 0–3 wetness/active-thaw score over the device area and a binary obstruction flag. The wetness model was developed from 247 quality-approved images, with complete dates kept together during training and validation. It used a fixed pretrained image representation followed by two conventional regression models (Methods). Fig. 4a shows predictions for images excluded from their corresponding training fold. The complete pipeline was then frozen and tested on a separate 48-image manifest spanning 37 dates. Five images with obstruction were excluded, leaving 43 images across 36 represented dates.

**Fig. 4:**
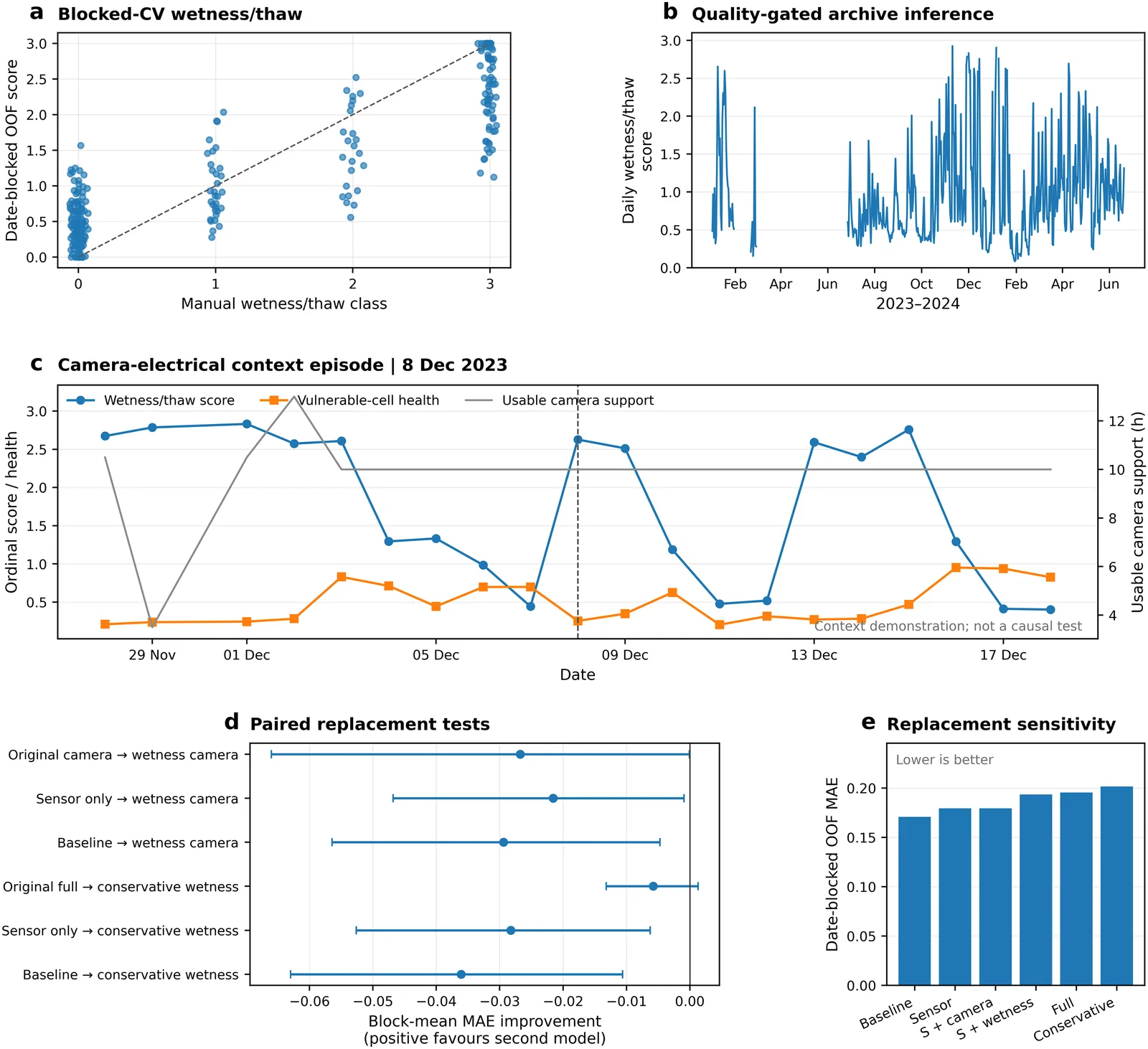
Camera wetness/thaw validation and predictive test. (a) Date-blocked out-of-fold development predictions *versus* manual class; the separate date-disjoint hidden audit is reported in the text. (b) Quality-gated daily archive inference. (c) Camera–electrical context on 8 December 2023. (d) Paired block-MAE differences for replacement tests. (e) Out-of-fold prediction error across model configurations.

On this unseen set, the mean absolute error (MAE) was 0.344 on the 0–3 scale. Rank agreement was high (Spearman *ρ* = 0.899), agreement after accounting for the ordered classes was also high (quadratic-weighted *κ* = 0.877), and the area under the receiver-operating-characteristic curve for wetness presence was 0.969. Most errors were between neighbouring severity classes. These metrics show that the model reproduced the manual wetness/thaw labels on dates that were not used for development.

Fig. 4b applies the frozen model to the full archive. Of 18 939 readable images, 10 067 quality-approved daytime observations were retained across 395 days. Missing periods were left missing rather than interpolated. Fig. 4c shows a predeclared example on 8 December 2023. The device area was visibly wet or actively thawing while vulnerable-cell health, the same-day median peer-referenced health of C2, C4, C5, C7 and C8, was 0.254, above the predefined at-risk floor of 0.15; the selection rule also required the date to lie more than 21 days from every terminal transition. Across 392 dates, morning wetness also agreed with independent nocturnal sensors. Spearman *ρ* was −0.571 with the minimum dew-point margin and 0.536 with mean relative humidity. The camera therefore measured a physically consistent surface state.

This valid wetness measurement did not improve short-term electrical prediction. All blocked health models used the same ridge-regression estimator and evaluation scheme; only the input variables changed (Methods). Fig. 4d shows the paired replacement tests. Relative to the sensor-only model, replacing the earlier camera proxy with validated wetness changed the block-mean MAE by −0.0216. Positive values would favour wetness, so the negative value indicates slightly worse prediction. The two-sided sign-flip randomisation test gave *p* = 0.101. With 12 independent date blocks, the analysis had 80% power to detect an MAE improvement of 0.0348. Fig. 4e reports the out-of-fold MAE: 0.171 for the electrical-history/seasonal baseline, 0.179 for sensor-only, 0.179 for sensor plus the original camera variables, 0.193 after wetness replacement, 0.195 for the original full model and 0.202 for the conservative full replacement. Neither the original image variables nor the validated wetness score improved three-day health prediction beyond electrical history and environmental sensors.

The full archive still clarifies exposure context. C2 transitioned during the strongest sustained thermomechanical period, and C8 during the most humid period. However, matched control windows were also elevated. The available terminal events therefore cannot assign failure to one short compound episode. This result is limited to the tested cells, inputs, endpoint and three-day horizon. Sections S7–S17, together with Fig. S8–S9, S11 and S15–S17, report the annotation rules, hidden-audit uncertainty, archive application, replacement tests and sensor comparison.

### 2.5. Two climates, two outcomes

The same 66 day winter window exposed the two device sets to different climates. Fig. 5a shows a mean device temperature of 4.9 °C in the Alpine deployment and 19.6 °C at Paterna. The Alpine devices crossed freeze–thaw conditions on 32 days; Paterna recorded none. Paterna also received more modelled plane-of-array energy, 814 *versus* 638 MJ m^−2^. Fig. 5b shows the moisture contrast. Mean night-time vapour pressure was 0.882 kPa at Paterna and 0.376 kPa in the Alpine environment.

**Fig. 5:**
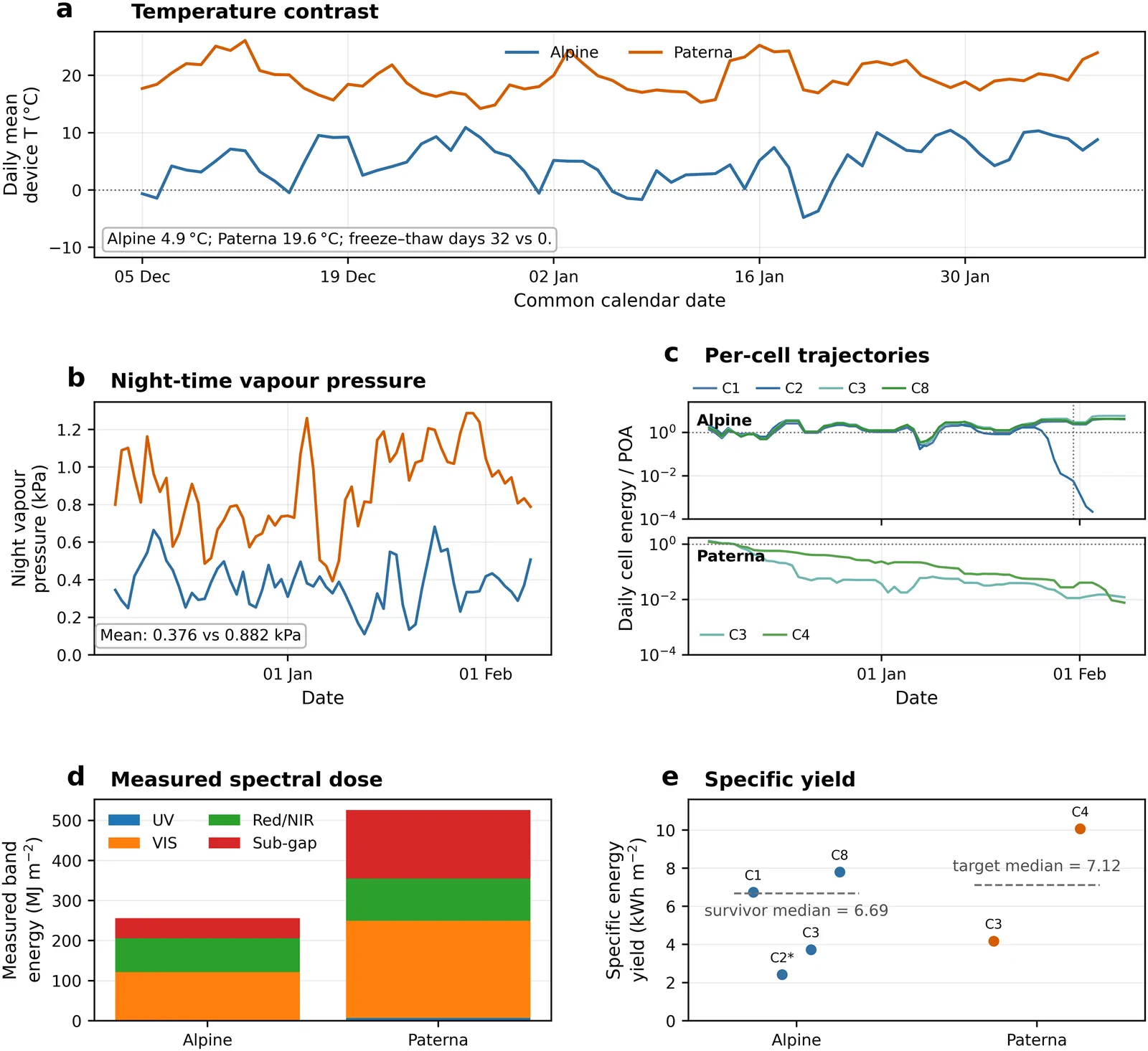
Two-site exposure and per-cell outcomes over the common 66 day window. (a) Daily device temperature and freeze–thaw contrast. (b) Night-time vapour pressure. (c) Normalised per-cell energy responsivity shown as separate Alpine and Paterna subpanels for readability; the Paterna subpanel is restricted to target cells C3/C4. (d) Paired spectral-band energy on 51 common valid dates. (e) Per-cell specific yield; the dashed Alpine line is the integrated same-day median of survivor trajectories C1/C3/C8 (6.69 kWh m^−2^), whereas the Paterna line is the frozen C3/C4 daily-median integral from the earlier Paterna daily-summary yield pipeline (7.12 kWh m^−2^). The asterisk marks Alpine C2, whose record ends at its 31 January terminal marker and therefore covers only part of the window.

The cell trajectories differ more clearly than the aggregate energy. In Fig. 5c, over the common 66 day window, Alpine C1, C3 and C8 recover after deep winter suppression, while C2 fails on 31 January. Paterna target cells C3 and C4 decline persistently throughout the same window. Fig. 5d shows spectral dose over the 51 dates with valid spectra at both sites. Paterna received more energy in every measured band and a UV dose of 6.62 rather than 1.74 MJ m^−2^. These band doses are instrument-relative: the measured bands captured 0.51 and 0.79 of plane-of-array energy at Alpine and Paterna, respectively (Table S18b), so the UV contrast is not used for causal attribution. Fig. 5e reports the per-cell yields. The dashed Alpine value (6.69 kWh m^−2^) is the common-window integral of the same-day median energy density across survivors C1/C3/C8; it is not the median or mean of the four plotted per-cell integrals. The dashed Paterna value (7.12 kWh m^−2^) is the C3/C4 daily-median integral from the archived Paterna daily-summary yield pipeline (Methods 5.11). Because the two summaries use different selected cohorts and source tables, they are not like-for-like site-level yield estimates. The per-cell integrals nevertheless overlap, while the temporal trajectories differ strongly. Heat, moisture, UV exposure and deployment history co-varied with degradation, so the paired design cannot isolate their individual contributions or support a population-level climate ranking.

All 66 common dates contribute to the physical and energy comparisons. Spectral totals use only the 51 dates with valid measurements at both sites. Irradiance was compared on the same modelled plane, humidity overshoots were retained in the raw Paterna record, and Alpine camera states were not imputed to Paterna. Section S18, Fig. S18 and Tables S18a, S18b provide the harmonised audit.

The Paterna side-camera archive provided a second-site transfer test of the Alpine optical–electrical correspondence. C3 and C4 were selected as the electrical target cells because they retained the strongest electrical performance during the early Paterna deployment, whereas the other six cells underwent rapid electrical loss. All eight cell regions were processed identically by the optical pipeline. Only C3 and C4 entered the locked transfer classification; the remaining Paterna electrical channels are shown as descriptive context in Tables S19b and S19c. The transfer inference is therefore limited to C3/C4 and is not an eight-cell population estimate.

Before the electrical onsets were matched, we checksum-froze the inventory of 3168 readable frames, the registration, cell and reference regions, quality thresholds, daily support and pruned exact linear time (PELT) changepoint settings with a radial-basis-function cost. Of these frames, 833 passed the primary quality gates, and 65 of 66 days had at least four accepted frames. Electrical decline began on 14 December for C3 and 21 December for C4. Their optical changepoints occurred on 1 and 3 January, 18 and 13 days after electrical onset. Both were outside the predeclared −7 to +2 day matching window. The Alpine optical–electrical timing therefore did not transfer in these two target trajectories. With a 10 day minimum segment and a 5 December series start, the earliest detectable changepoint is 15 December, so only 15 and 16 December lay inside the C3 matching window. Other device regions changed during the same 23 December–3 January period, while both scene controls remained stable. This pattern indicates a broad device-area optical transition rather than a C3- or C4-specific precursor. The two target changepoints were only 2 days apart although their electrical onsets were 7 days apart, which is more consistent with a shared device-area change than with a fixed per-cell optical lag. Section S19 and Fig. S19 report the full timing and target-provenance record.

Exploratory analyses did not recover a transferable optical predictor. Upper-tail colour change, changed-area fraction, spatial b* heterogeneity and gradient change showed no onset-specific C3/C4 pattern. After each site's shared daily variation was removed, substrate-specific optical residuals were uncorrelated with subsequent electrical loss (*ρ* = 0.055 Alpine; 0.002 Paterna). Adding these residuals increased blocked MAE from 0.321 to 0.332 at the Alpine site and from 0.717 to 0.721 at Paterna. The Paterna model reached 0.737 when the spatial residuals were also included. These exploratory analyses do not alter the frozen Alpine operating characteristics or the frozen Paterna transfer result. Section S20 and Fig. S20 report the complete analysis.

## 3. Discussion

Continuous field monitoring separated the dominant reversible winter common mode from persistent cell-specific perovskite solar-cell failure. The fixed C1/C3 reference did not change as other cells failed. Because C1 and C3 were chosen retrospectively as the two full-campaign electrical survivors, this is a retrospective diagnostic rather than a prospective fleet rule, and any degradation shared by both controls would cancel. Their raw responsivity nevertheless recovered in spring to 107% and 109% of the autumn medians, confirming that the dominant winter excursion was reversible. A smaller shared irreversible component cannot be excluded, and the early October cohort-wide loss did not recover. C8, which later failed, showed the same winter rebound, so the rebound is not an artefact of selecting the two final survivors.

The locked retrospective colour replay matched three of the four Alpine terminal markers, with a median optical-first offset of 4 days and four other device-window changepoints over 2.90 window-years. The result is marker-dependent. Against the earlier daily-persistent marker, C2 is coincident, C8 is electrical-first by 2 days, and the only optical-first change, C5/C6 by 3 days, is synchronous with the non-failing C3/C4 window; C5 raw responsivity collapses on 5 March. The evidence therefore supports day-scale optical/electrical co-evolution around terminal loss, not a device-specific advance precursor. The retrospective string analysis compares optical-date and terminal-date switching: 0.588 kWh m^−2^ gross and 0.562 kWh m^−2^ net after unmatched changepoints, or 4.17% of the terminal-date-bypass campaign energy. This is an engineering benchmark for prompt, low-loss switching or inspection, not energy attributed to advance warning. The missed C7 event also shows weaker correspondence during low-light winter conditions and for a different failure type.

The camera analysis separates measurement from prediction. A date-disjoint hidden test confirmed that passive RGB images measure visible wetness and active thaw. The score also agreed with independent humidity and dew-point measurements. For the tested cells, inputs, endpoint and three-day horizon, adding wetness or the environmental sensors did not improve prediction over electrical history and seasonal terms; longer time scales were not tested. Chromatic changepoints therefore remain retrospective diagnostic correlates, not a prospectively validated detector, degradation mechanism or lifetime model.

The two sites differed in both cell trajectories and replay outcome. During the common 66 day winter window, Alpine C1, C3 and C8 recovered after winter suppression, while Paterna targets C3 and C4 declined persistently. Per-cell integrated yields overlapped, but the 7.12 kWh m^−2^ Paterna line summarises C3/C4 only and is not a site-level statistic; similar integrated yield therefore does not imply similar temporal behaviour or site-level yield parity. The Alpine optical–electrical timing did not transfer to C3/C4: their optical changes followed electrical onset and formed part of a broad device-area transition across all eight optical regions. Additional spatial features did not restore predictive value. Temperature, moisture, spectral exposure and deployment history co-varied, so no single causal stressor or population-level climate ranking can be assigned. At both sites, no device-specific colour change demonstrably preceded electrical loss. Multi-season, larger-cohort and module-scale tests are needed before generalising the climate response or timing rule.

Endpoint XRD resolved more than one recovered failure state. Under the confirmed map, C1 and C3 retained the strongest perovskite reflections, while C2, C5 and C8 were strongly PbI_2_-rich after photocurrent-dominated loss. C7 retained a lower finite PbI_2_/perovskite ratio after mixed-mode failure. Because C7 also had the longest interval between failure and recovery, the endpoint contrast cannot separate field degradation from storage and preparation effects. The measurements therefore describe recovered states, not the initiating field chronology.

The framework links each inference to a separate test. The Alpine terminal calendar is fixed before the optical replay; the matched fraction, optical-first offsets, changepoint exposure, earlier-marker timing and same-date specificity are reported together. Camera wetness is validated separately from electrical prediction, and the optical replay is transferred to Paterna without retuning to its onset results. Sample identity is preserved for endpoint XRD, and the string benchmark includes the cost of unmatched changepoints. Table 1 summarises the supported conclusions. Matched-window and delayed-response analyses follow established case-crossover and distributed-lag methods.^84,85^ Sections S21–S23 provide the regime-evidence hierarchy, machine-readable replay protocol and archive/reporting checklist, including an event-resolved ISOS-O template that keeps per-cell trajectories, environmental context, optical quality control, event definitions and endpoint provenance linked.

**Table 1:** Validation framework and supported interpretation

Question	Primary test	Supported conclusion
Validity	Hidden image audit, quality gates and sensor comparison	Passive RGB resolves visible wetness/thaw and measurement context; it does not identify perovskite chemistry
Prediction and transfer	Locked retrospective Alpine replay; daily-persistent timing and specificity checks; Paterna transfer replay; date-blocked replacement tests	The Alpine replay matches 3/4 retrospective terminal markers under the frozen calendar, but 1/4 against the earlier daily-persistent marker: C2 coincident, C5/C6 3 d optical-first, C8 2 d electrical-first, C7 missed. The only optical-first change is synchronous with the non-failing C3/C4 window, so no device-specific advance precursor is established. In Paterna, C3 and C4 change 18 and 13 d after electrical onset, outside the −7 to +2 d window. Validated wetness does not improve three-day prediction
Endpoint interpretation	Confirmed sample-to-cell map, cell-resolved diffractograms and descriptive PbI_2_/perovskite peak-height ratios	C1 and C3 retain the strongest perovskite reflections and lowest PbI_2_/perovskite ratios; C2, C5 and C8 are PbI_2_-rich lower-bound endpoints after photocurrent-dominated loss; C7 retains a lower finite ratio after mixed-mode failure. The endpoint therefore supports more than one recovered state
Engineering value	Retrospective eight-position switching-calendar benchmark including the four unmatched changepoints	Optical-date *versus* terminal-date bypass differs by 0.588 kWh m^−2^ gross and 0.562 kWh m^−2^ net after a 0.025 kWh m^−2^ unmatched-changepoint cost, or 4.17% of terminal-date-bypass campaign energy under the tested string policy. This is an engineering benchmark between two retrospective switching calendars, not energy attributed to advance camera warning. Components are rounded independently

## 4. Summary

Continuous optical–electrical monitoring separated dominant reversible winter modulation from persistent cell-specific perovskite solar-cell failure. The retrospective fixed C1/C3 reference cannot detect degradation shared by both controls, but their raw responsivity recovered to 107% and 109% of the autumn medians, confirming that the dominant winter minimum was reversible; the early October cohort-wide loss did not recover. In Alpine, the locked retrospective colour replay matched three of four terminal markers with a median optical-first offset of 4 days. That result depends on marker choice: the earlier daily-persistent marker makes C2 coincident, C8 electrical-first, and leaves only the C5/C6 window optical-first by 3 days; the same-day C3/C4 change removes device specificity. The string analysis therefore remains a retrospective engineering comparison (0.588 kWh m^−2^ gross, 0.562 kWh m^−2^ net, 4.17%), not energy attributed to advance warning. Passive RGB measured wetness and thaw reproducibly but did not improve three-day health prediction. The Alpine optical–electrical timing also failed to transfer to Paterna C3/C4, where optical changes followed electrical onset by 18 and 13 days. Endpoint XRD separated PbI_2_-rich photocurrent-loss endpoints C2/C5/C8 from the lower-ratio mixed-mode C7 state. Over the common winter window, Alpine survivors recovered while Paterna C3/C4 declined; overlapping per-cell integrated yields therefore do not imply equivalent trajectories or site-level yield parity. The two-site comparison does not support a population-level climate ranking, and the observed contrast is reported for these cells, this encapsulation and this 66 day window only. Together, the results provide an auditable framework that separates weather modulation, cell-specific failure, optical timing and specificity, cross-site transfer and predictive value.

## 5. Methods

### 5.1. Device fabrication and encapsulation

The Alpine and Paterna field devices were fabricated in the same batch using the process described below. For the Alpine cohort, four patterned FTO-glass substrates were prepared, each carrying two independently contacted cells. The n–i–p stack was Glass/FTO/compact TiO_2_/mesoporous TiO_2_/ (FA_0.95_Cs_0.05_)PbI_3_/PEAI/Spiro-OMeTAD/Au. After deposition of the compact and mesoporous TiO_2_ layers, the mixed-cation iodide absorber was deposited and crystallised by FIRA using the frozen batch pulse programme. The process followed the reported antisolvent-free planar and mesoscopic FIRA protocols, pulse-time nucleation study and thermally controlled FAPbI_3_ framework.^51–56,59^ PEAI surface treatment was followed by Spiro-OMeTAD deposition and thermal evaporation of Au. The active area used for electrical normalisation was 0.16 cm^2^ per cell.

After electrical screening, the devices received a conformal parylene/TiO_2_ inner barrier. A 10 µm Parylene C layer was deposited at room temperature under primary vacuum by vapour-phase polymerisation using a Comelec C-30-S system at the EPFL Center of MicroNanoTechnology. A thin-glass lid was then bonded with 3M Thermally Conductive Adhesive TC-2810 applied as a perimeter seal and allowed to cure for 24 hours at room temperature.

### 5.2. Outdoor platforms and deployment

Identical open-cavity holders exposed the active cell surfaces to the natural environment while keeping the electrical contacts mechanically fixed. Each platform accommodated eight cells, a device-plane reference cell, a cosine corrector positioned behind its substrate half, a broad-band pyranometer, ambient temperature and relative-humidity sensing, a Pt probe on the reference-cell back contact, and a device-plane UV/visible/NIR spectrometer. Both platforms carried a fixed RGB camera; the Alpine archive drove the main optical analysis, while the Paterna archive was reserved for the replay locked before formal electrical-onset unblinding (Section 2.5). At Paterna, C3 and C4 provided the paired electrical trajectories, while all eight registered cell regions were retained for the optical replay. The paired comparison used the identical 5 December 2023–8 February 2024 calendar window. Both systems were remotely accessible for acquisition checks. The public project page records the paired-site platform and instrumentation.^83^

### 5.3. Electrical acquisition and quality control

Maximum-power-point records were acquired at approximately 10.5 s cadence. Single-direction *J*–*V* scans were recorded periodically and processed to obtain *V*_oc_, *J*_sc_, fill factor, maximum power and PCE. PCE was retained only above the frozen irradiance gate. Implausible scans were excluded using the archived QC flags, including *V*_oc_ outside −0.2 to 1.4 V and |*J*_sc_| above 40 mA cm^−2^. Cell energy was integrated without bridging timestamp gaps longer than 1.01 h. Terminal markers were set retrospectively from persistent cell-level loss and then treated as censored outcomes where the campaign ended before a complete post-event window.

### 5.4. Irradiance, spectrum, temperature and humidity

Logger timestamps were retained in local CET/CEST-as-recorded time. The pyranometer night offset was estimated from the local 00:00–04:00 median and removed before isotropic plane-of-array transposition.

Device-plane spectral irradiance was measured with a StellarNet UV–visible–NIR spectrometer at the Alpine site and an Optosky ATP-series instrument at Paterna, both using cosine-corrected inputs mounted co-planar with the cells. Alpine spectra were acquired at 250 ms with ten-scan averaging; Paterna spectra used a non-saturating 160 ms integration time without filtering.

Each instrument was calibrated independently after dark subtraction. The StellarNet response was derived from a calibrated tungsten–halogen reference lamp and checked against a nominal one-sun spectrum. The Optosky response was corrected using archived dark and one-sun spectra together with the ASTM G173 global-tilt spectrum normalised to 1000 W m^−2^. Signals were normalised by integration time before applying the instrument-specific calibration factors.

Residual baseline drift was estimated below 285 nm. Calibrated spectra were integrated over 300–400 nm, 400–650 nm, 650–840 nm and 840–1100 nm. These limits separate the ultraviolet range from the above-bandgap response and from the sub-bandgap region beyond the FAPI absorption edge. Cross-site comparisons used band-integrated energies and spectral fractions rather than wavelength-by-wavelength ratios, and the spectral bands were not forced to close to the broader pyranometer response.

Temperature values outside −50 to 80 and invalid humidity records were retained in the raw archive, flagged and masked without interpolation. Finite humidity overshoots were clipped to 0–100% only for physical calculations.

### 5.5. Camera acquisition and chromatic extraction

The fixed Alpine analysis camera acquired images at a nominal 30-min cadence. A separate side camera supplied daylight snapshots approximately every 10 min for remote station inspection; these images were not substituted for the analysis archive. Camera filenames were aligned to the logger clock using the frozen offset. Frozen quality gates excluded night or infrared frames, severe glare, blur, obstruction and images outside the accepted visual range.

Four fixed device-window regions and one white reference port were verified to remain registered within one pixel. The effective device-plane ROI sampling was 0.25 mm. Device and reference-port regions were fixed and non-overlapping. RGB values were normalised to the white holder and converted to CIE Lab. The Alpine chromatic series was the daily median of b* (device window) – b* (reference port), restricted to images that passed the visible/day gate.

The Paterna validation used the complete 5 December 2023–8 February 2024 camera inventory. Holder geometry, eight active-area regions, two white-holder references and one background control were fixed before formal electrical-onset unblinding. Affine registration and image-quality thresholds were calibrated on six neutral frames from 4–5 December and then frozen. The primary Paterna signal was the daily median of cell b* minus the primary white-reference b* across quality-passed frames; L*, a*, chroma, a secondary reference and dispersion metrics were retained as supporting channels. A valid day required at least four accepted frames.

### 5.6. Survivor-referenced electrical health and terminal events

The survivor-referenced health trajectories use a fixed C1/C3 peer reference. C1 and C3 were selected retrospectively because they remained electrically active through the full Alpine campaign. For C2, C4, C5, C7 and C8, same-day photocurrent responsivity was divided by the median of C1 and C3; C1 and C3 each used the other control as reference. A fixed cell-specific normalisation scalar aligned the reference state using the first seven valid common-window days of each cell. C4 and the terminal cells therefore never entered the denominator, and the reference composition remained fixed as failures accumulated. C6 became electrically unusable on 2 October 2023 and was excluded from the health and terminal-event analyses but retained for optical mapping and endpoint XRD. Fig. 2a and 3b and the SI call this survivor-referenced health; it is also called peer-referenced health in this text. Both terms denote the same fixed C1/C3 reference.

The fixed reference removes dominant variation shared by the two controls but cannot identify irreversible degradation common to both. Absolute plane-of-array-normalised responsivity was therefore retained as an independent control. Over the pre-specified Fig. 2 seasonal windows (5 October–5 November, 1 December–15 February and 20 April–10 June), seven-day-median C1 and C3 spring responsivities were 107% and 109% of their autumn medians, respectively; the autumn window straddles the early October cohort-wide step and is not treated as a pristine pre-loss baseline. Reference sensitivity using C1 only, C3 only, a leave-one-out peer median and raw responsivity is reported in Section S4.

The retrospective terminal-marker calendar used downstream was C7, 16 November 2023; C2, 31 January 2024; C5, 8 March 2024; and C8, 27 May 2024. These markers were held fixed during the final Alpine optical replay. No separate dated record establishing when this four-date calendar was first set has been recovered; the authors report 14 June 2026, and the calendar is therefore treated as a retrospective outcome definition rather than a preregistered electrical endpoint. They were not all generated by the 14 day threshold shown for illustration in Fig. 2e: for C2, the frozen retrospective marker is 31 January; the 14 day median first falls below *θ* = 0.30 on 2 February, and the 14-recorded-day persistence requirement is met on 15 February (Table S4d). Rule-derived 7 day and 14 day rolling-median markers, re-derived without refitting the optical detector, preserved the same three matched events and the same four other device-window changepoints (Table S4c). For the earlier timing audit, an unsmoothed daily-persistent marker was defined *post hoc*, but again without optical refitting, as the first recorded day with *H*  <  0.30 followed by 14 consecutive recorded days below 0.30. This gives C7 = 16 November 2023, C2 = 27 January 2024, C5 = 5 March 2024 and C8 = 24 May 2024; relative to the fixed optical changepoints, C2 is coincident, the C5/C6 window is 3 d optical-first and C8 is 2 d electrical-first, with C7 missed, so the historical 1–14 d rule yields 1/4 rather than 3/4. C1 and C3 survived to the final powered record on 20 June 2024, while C4 remained a persistent partial-loss case without a terminal marker. Substrate, not the individual cell, was treated as the experimental unit for substrate-level optical analyses.

### 5.7. Analysis-locked Alpine chromatic replay

The Alpine chromatic analysis was retrospective and in-sample. The earliest recovered dated record in which the chromatic/electrical relationship was already documented is 17 July 2026. By 19 July 2026 the complete replay specification had been locked: visible/day-gated daily port-referenced b*, interpolation across gaps of at most 7 d, PELT segmentation with a radial-basis-function cost, penalty 8, minimum segment length 10 d, and one-to-one nearest-following matching to the fixed terminal-marker calendar within 1–14 d. The primary replay was not retuned after that lock. A separate *post hoc* specification audit tested the 2 March same-date synchrony across PELT penalty 4–12 and minimum segment length 7–14 d while retaining the historical detector and matching rule for the primary result. The archived Alpine changepoint date is the last day before the new segment, whereas the Paterna replay uses the first day of the new segment (Section S4.3). Changepoint exposure was four windows × 265 d = 2.902 window-years. The matched fraction used exact binomial confidence intervals, and the rate of other device-window changepoints used exact Poisson intervals. Section S4 provides the dated lock chronology and terminal-date sensitivity.

### 5.8. Paterna transfer replay

The Paterna image inventory, ROI and QC files were checksum-frozen before full-window extraction. Of 3168 readable images, 833 passed the primary quality gates; 65 of 66 days reached the four-frame minimum. The isolated invalid day, 8 January 2024, was linearly interpolated only for the optical changepoint sequence under the frozen one-day-gap rule. All eight cell signals were analysed identically using PELT–RBF, penalty 8, minimum segment length 10 d and no smoothing. The Paterna implementation reported the first day of the new segment; shifting to the previous-day convention would not change the transfer classification.

C3 and C4 were selected as the Paterna electrical target cells because they retained the strongest early electrical performance, and their target status was locked before formal electrical-onset unblinding. All eight optical cell regions were processed identically. Only C3/C4 entered the locked transfer classification; the remaining electrical channels are shown as descriptive context in Table S19c. The recovered lock establishes the ordering of target designation before formal unblinding; the author-reported dates are 23 June and 20 July 2026, respectively. The transfer inference is therefore limited to C3/C4.

Electrical onset was derived from the checksum-frozen historical Paterna responsivity file used at formal unblinding. For C3 and C4, the baseline was the median of the first seven calendar days and the threshold was 50% of baseline. Onset was the first of at least three consecutive days below threshold, provided that at least six of the seven days beginning at onset were also below threshold. No electrical interpolation was allowed. A qualifying optical event had to occur from 7 d before to 2 d after onset and could not lie within 2 d of a scene-control changepoint. Both-cell, one-cell and zero-cell matches were preclassified as full support, partial support and no transfer. The historical input is included with SHA-256 af6482ce5a8ffc9045f6a7569d81e74e8e84296b67bff9615e4cda02da99b1db, and the guarded unblinding script reproduces the 14/21 December onsets and the 18/13 d offsets. The later harmonised two_site_timeseries.csv used for paired-site figures is a separate input and was not used to recompute these onsets (Section S19).

### 5.9. Camera-state and wetness validation

The original five-label camera study used 135 environment-valid development images across 82 date or episode groups and a separate frozen 52-image audit. The observation-context workflow used principal-component features from the camera embeddings together with scalar features, with ridge regression for the ordinal scores and logistic regression for presence probabilities. Development splits kept the recorded date or episode group intact. The archived month-grouped gradient-boosted classifier is an earlier workflow and is not the source of these five-label results. A later targeted campaign defined an ordered 0–3 device-area wetness/active-thaw score and a binary obstruction flag. Dates, rather than individual images, were the blocking unit.

Images were represented with a torchvision ResNet-18 pretrained using ResNet18_Weights.DEFAULT. We applied the standard preprocessing associated with these weights and replaced the final classification layer with an identity operation. This produced a fixed 512-dimensional image embedding. No convolutional weights were retrained.

The wetness/thaw model combined the image embedding with the predefined auxiliary camera features. Its prediction was the equal-weight average of two models trained with the predefined class weights. The first was standardised ridge regression, with regularisation selected by grouped cross-validation from {1,10,100,1000}. The second was an ExtraTreesRegressor with 600 trees, minimum leaf size 3 and square-root feature subsampling. The averaged prediction was clipped to the frozen 0–3 range. A comparison model using only scalar image statistics was tested with the same date blocking.

The model and all preprocessing were frozen before the separate date-disjoint hidden set was annotated. Archive inference retained only quality-approved daytime frames, and missing intervals were not interpolated. In the health-prediction tests, the validated wetness score replaced the earlier device-region proxy. It was not added as an extra input beside the original camera variables.

### 5.10. Blocked prediction and matched episodes

The pre-specified prediction endpoint was the median survivor-referenced health over the current 3 d minus the median over days +1 to +3. The analysis was restricted to at-risk dates on which vulnerable-cell health remained above 0.15.

The model configurations differed only in their inputs. The baseline used electrical history and seasonal terms. The sensor-only model added irradiance, device temperature and humidity. A third model added the original camera variables. In the replacement models, those camera variables were replaced by the validated wetness score. Every configuration used ridge regression with median imputation and feature standardisation. The comparison therefore tests the information carried by the inputs rather than differences in model capacity.

Regularisation was selected internally with RidgeCV from 49 logarithmically spaced values between 10^−3^ and 10^3^. Five-fold GroupKFold produced predictions for held-out data while keeping each 14 day calendar block entirely in either training or testing. Paired uncertainty was evaluated over 12 non-overlapping date blocks. The primary comparison reported differences in block-mean MAE, cluster-bootstrap intervals, block sign-flip randomisation tests and the minimum improvement detectable with 80% power. The frozen V20.9.4 paired tests used 50 000 Monte Carlo sign-flip randomisations with the standard *p* = (*k* + 1)/(*B* + 1) correction. The random-number generator was initialised with seed 21 712 (the frozen V20.8 seed 20 808 plus 904), and the cluster-bootstrap intervals used 10 000 block resamples from the same frozen random-number stream. A later all-pairs concordance audit, reported in Section S16, enumerated all 2^12^ = 4096 sign configurations exactly; this secondary audit does not replace the frozen primary *p* values. Holm adjustment across the 15 stored comparisons gives a minimum adjusted *p* of 0.279, so no contrast remains significant after multiplicity correction (Table S16a).

Frozen compound-event dates and controls were not rematched after wetness validation. The matched-episode results are descriptive and cannot identify a unique causal stressor. The matched-window and lag-aware comparisons follow case-crossover and distributed-lag design principles.^84,85^

### 5.11. Two-site comparison

The paired comparison used 66 common calendar dates. Modelled plane-of-array energy used the same geometric basis at both sites; Alpine plane-of-array irradiance was not compared directly with Paterna global horizontal irradiance. Per-cell specific yield was primary. The Alpine C1/C3/C8 display summary is the integral of the same-day median cell energy density over the common window (6.688 kWh m^−2^). The Paterna C3/C4 per-cell yields (4.177 and 10.069 kWh m^−2^) and target daily-median integral (7.123 kWh m^−2^) come from the archived Paterna daily-summary yield pipeline, included in the data package with SHA-256 f327fc419a776367872e581c51b872a7c53e26cff8029231d4ece10a27ae822a. The later harmonised two_site_timeseries.csv is used for paired-site trajectories but not substituted for this yield source (Section S18). Because the summaries use different cohorts and source tables, they are descriptive rather than site-level estimates. Spectral-band totals use only the 51 dates with valid spectra at both sites. No Alpine RGB surface state was imputed to Paterna.

### 5.12. Exploratory spatial and common-mode analyses

These analyses were initiated only after the frozen Paterna transfer result and cannot alter it. A pixelwise illumination-corrected Lab baseline was the median of 80 quality-passed Paterna frames acquired from 5–11 December. For each cell and frame, exploratory features included the 90th percentile of Δ*E*_76_, the fraction of pixels with Δ*E*_76_ > 5, spatial b* interquartile range and the 90th percentile of gradient change. Daily medians were compared for C3 and C4 against the six other cell regions before, during and after the frozen electrical onsets.

For the exploratory cross-site analysis, the four substrates A–D were the experimental units (C1/C2, C3/C4, C5/C6 and C7/C8). For the Alpine C5/C6 substrate, C6 had no valid positive electrical baseline and therefore did not contribute to the substrate electrical aggregate; the co-located C5 series defined the electrical value, while the optical substrate mapping was retained. Daily optical and log-normalised electrical signals were divided into two parts: the site median, which represents variation shared across the site, and each substrate's deviation from that median. Future electrical loss was the current three-day median electrical deviation minus the median on days +1 to +3. Spearman correlations were interpreted descriptively.

All baseline and augmented comparisons used ridge regression with fixed regularisation (*α* = 1). The shared baseline included the current three-day electrical deviation, the site-level optical median and plane-of-array energy. The augmented models added the substrate-specific optical deviation. For Paterna, a further model also added two spatial optical deviations. All models used the same ten date-disjoint test blocks and excluded the 3 d on both sides of each held-out block from training. Changes in MAE therefore reflect the added optical variables rather than a change in estimator.

### 5.13. Endpoint X-ray diffraction

Devices were recovered on 1 July 2024, eleven days after the final powered record, and stored unopened in a drybox in the dark at room temperature. On 20 January 2025, adhesive tape was used to remove the electrical contact and upper encapsulation layer, and sequential layer removal exposed the perovskite. Residual Spiro-OMeTAD may remain; no solvent was used. Endpoint XRD was acquired on 21 January 2025. All cells underwent the same post-recovery storage and preparation.

Local PbI_2_/perovskite peak-height ratios were used as descriptive indicators of the recovered state, not as quantitative phase fractions. The C2, C5 and C8 ratios were treated as lower bounds because their perovskite reflections approached the local background. Endpoint XRD defines the recovered state and does not reconstruct historical field-state phase fractions or the initiating sequence.

### 5.14. Retrospective string decision-value scenario

A hypothetical eight-position series string was reconstructed from common-time maximum-power-point current and voltage and valid *J–V*-derived *J*_sc_, *V*_oc_ and FF descriptors. Energy density was normalised to the nominal active area of all eight string positions. Optical-date bypass was compared with terminal-date bypass using the frozen dates. In both policies, C6 was unavailable and bypassed from 2 October 2023; this date was not treated as a frozen terminal transition. The primary false-action scenario bypassed the stronger still-active cell in each unmatched two-cell window for one day. Delay and bypass-voltage-drop sensitivities were also evaluated. The decision-value estimate applies to the four observed Alpine terminal events and the idealised switching policy, not to population-level deployment benefit.

### 5.15. Statistics and computational freeze

Each analysis used a defined freeze point. The Alpine chromatic detector was developed retrospectively and locked by 19 July 2026; the primary replay was not retuned afterward, and terminal-date sensitivity was evaluated without refitting the detector. The endpoint XRD interpretation rules were fixed before the cell-resolved assignment was interpreted. The Paterna image, changepoint, electrical-onset and matching rules were checksum-frozen before formal electrical unblinding.

Wetness/prediction confidence intervals, blocking variables, event windows, thresholds, health horizons, at-risk cut-offs and model families were fixed before the final analyses. The separate Alpine penalty/minimum-segment check in Section 5.7 tested same-date synchrony without retuning the historical replay. Spatial and common-mode analyses were performed after the frozen Paterna transfer result and were treated as exploratory. Statistical tests are two-sided unless stated otherwise.

The accompanying SI and Code package contains the machine-readable figure inputs, derived tables, model reports, analysis scripts and checksum manifests needed to audit the reported values and regenerate the quantitative figures, together with the historical Paterna onset input, the archived Valencia daily power-sum file and the raw endpoint diffraction patterns. The complete long-form camera and sensor streams remain in the project repository because of their size.

## Author contributions

S.S.-A.: conceptualization; methodology; investigation; data curation; formal analysis; visualization; project administration; writing – original draft; writing – review & editing. P.P.B.: methodology; investigation; resources; writing – review & editing. R.A.: methodology; investigation; resources; writing – review & editing. J.N.-G.: methodology; investigation; resources; writing – review & editing. All authors reviewed and approved the manuscript.

## Conflicts of interest

The authors declare no conflicts of interest.

## Supplementary Material

EE-019-D6EE05477C-s001

## Data Availability

The data and derived records required to audit the figures and reported analyses are archived in the Supplementary information (SI) and Code deposit at Zenodo, https://doi.org/10.5281/zenodo.23084295. A top-level README and a FILES_AND_SCRIPTS.csv source-to-script map identify the files and scripts behind each revised analysis, and a packaged verification script reproduces the key results. The package contains frozen chromatic and terminal manifests, the full 48-image hidden-test manifest and 43-row evaluated wetness-prediction table, wetness-validation and paired-model tables, paired-site replay and Paterna optical/spatial metrics, the descriptive eight-channel Paterna electrical context table, raw endpoint diffraction patterns and cell-level endpoint ratios, the switching-benchmark ledger, representative camera inputs and checksum manifests. The historical Paterna electrical input used at formal unblinding and the archived Valencia daily power-sum file used for Fig. 5 are included with their SHA-256 checksums; the harmonised two-site table is supplied separately for paired-site analysis. The complete long-form sensor streams and full image archive are maintained with the project record because of their size and are available from the corresponding author upon reasonable request. Supplementary information (SI) is available. See DOI: https://doi.org/10.1039/d6ee05477c. Code availability: the same Zenodo deposit (https://doi.org/10.5281/zenodo.23084295) includes the analysis and plotting code used for the reported quantitative figures and audits, including Paterna registration, extraction, transfer and common-mode analyses, together with a shared plotting module and machine-readable inputs. Scripts requiring the complete camera archive, together with master-table construction and the remaining long-form pipeline, are maintained with the project repository and are available from the corresponding author upon reasonable request.
