# Supplementary material for: Continuous optical–electrical field diagnostics separate environmental modulation from irreversible perovskite solar-cell failure
Source: Energy Environ Sci. 2026 Oct 6;19(21):7447–65. doi: 10.1039/d6ee05477c (PMC13651458; doi:10.1039/d6ee05477c)
Supplement: Supplementary file 2 [file EE-019-D6EE05477C-s001.pdf]

Supplementary Information (SI) for Energy & Environmental Science.
This journal is © The Royal Society of Chemistry

Continuous optical–electrical field diagnostics separate environmental modulation from irreversible perovskite solar-cell failure

Jaume Noguera-Gómez[1], Rafael Abargues[2], Pablo P. Boix[3], and Sandy Sánchez-Alonso[4,5,*]

[1]School of Physical and Mathematical Sciences, Nanyang Technological University, Singapore 637371

[2]Instituto de Ciencia de los Materiales de la Universitat de València (ICMUV), Valencia, Spain

[3]Instituto de Tecnología Química (ITQ, UPV-CSIC), Valencia, Spain

[4]School of Engineering and Architecture of Fribourg, HES-SO, Fribourg, Switzerland

[5]Laboratory of Photonics and Interfaces, École Polytechnique Fédérale de Lausanne, Lausanne, Switzerland

[*]Corresponding author: sandy.sanchez@hefr.ch

S1. Evidence hierarchy and computational freeze

The Alpine and Paterna field cohorts were fabricated in the same batch and used the FTO/compact TiO_2/mesoporous TiO_2/ $(FA_{0.95}Cs_{0.05})PbI_3$/PEAI/Spiro-OMeTAD/Au architecture described in the main Methods. The absorber was crystallised by the batch-frozen FIRA sequence, following the antisolvent-free process, pulse-time nucleation analysis and solution-crystallisation framework.[7, 8, 9]

The camera pipeline separated visible environmental states from latent device degradation. Blocked development and a frozen audit established the observable labels, consistent with stability-reporting and FAIR-data principles.[1, 2, 10] A date-disjoint hidden set then tested the device-area wetness/thaw model before archive inference.

The frozen primary analysis remained the main robustness test. The wetness score replaced the earlier surface proxy in a sensitivity test because both use the same camera embeddings and auxiliary predictions. The freeze kept the event threshold, endpoint, at-risk criterion, date blocks, model settings and control matching unchanged.

Cross-section 1

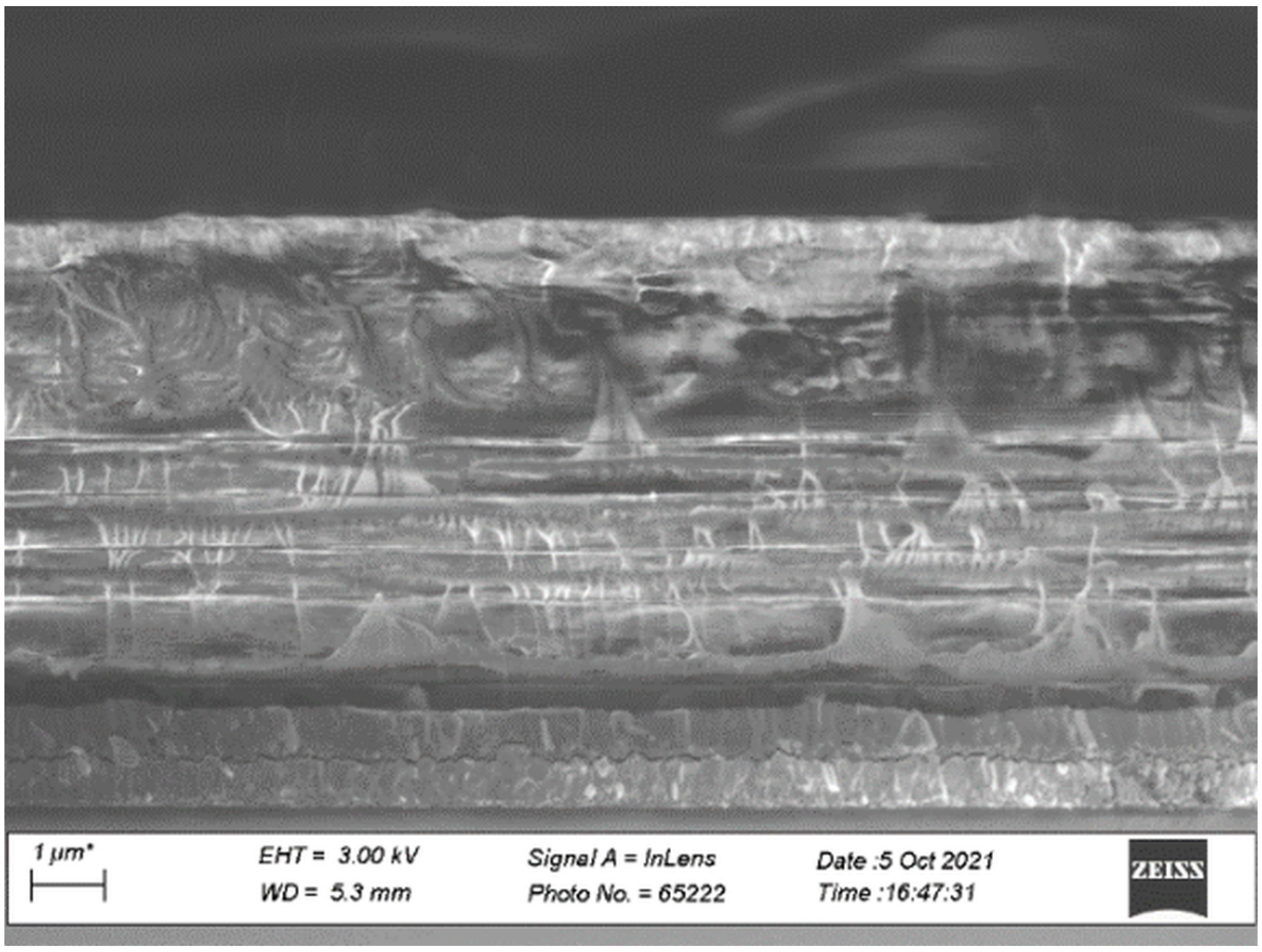

Cross-section 2

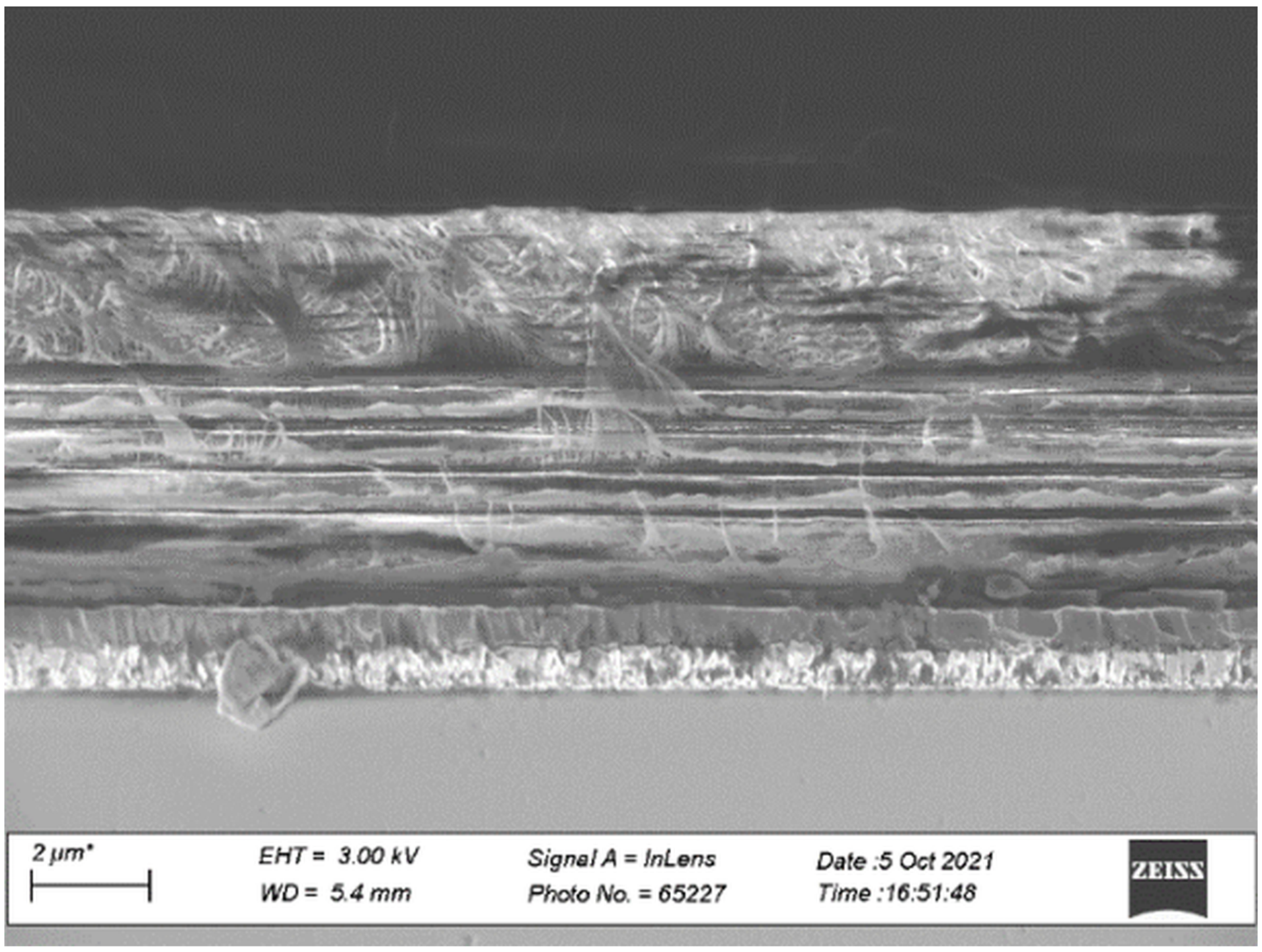

Figure S1. Cross-sectional SEM of the inner multilayer coating, with acquisition labels and scale bars.

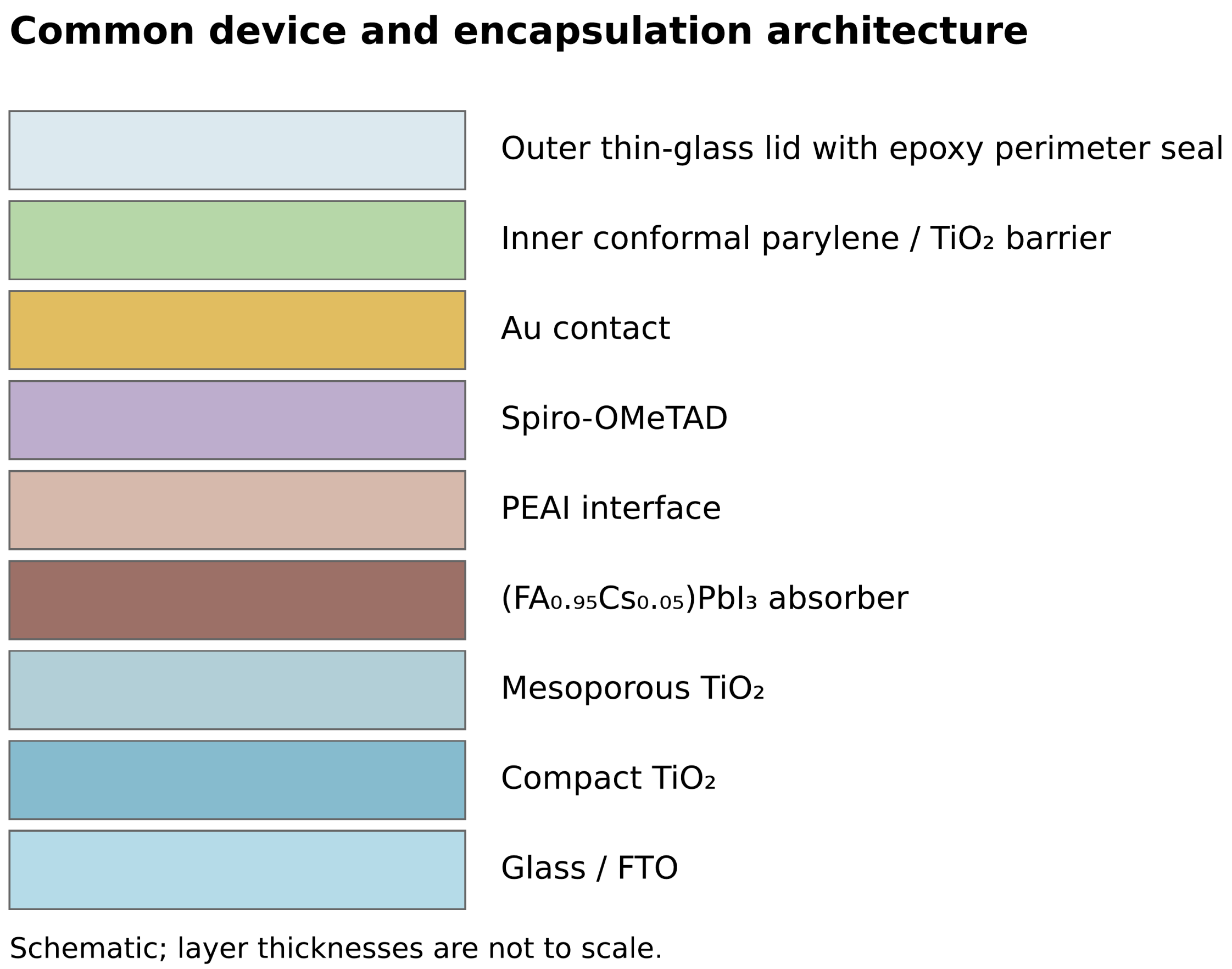

Figure S2. Shared device architecture and dual encapsulation. The inner conformal barrier and outer thin-glass/epoxy lid are distinct components; thicknesses are not to scale.

S2. Camera archive and image-quality control

The Alpine camera archive contained 18,965 JPEG images. Image decoding and pretrained embedding extraction succeeded for 18,939 images; the 26 unreadable files were confined to late June 2024. Existing night/infrared, glare, blur and out-of-distribution gates were applied before environmental-state inference. The same frozen quality decision was used for all archive labels and for the new wetness model.

Supplementary Fig. S3 shows two views of each deployment: Alpine (Lac des Toules / Grand-St-Bernard) and Valencia (Paterna). Supplementary Fig. S4 links the side-camera views to the Valencia telecontrol cabinet, sample holder and common acquisition workflow. Images, electrical outputs and environmental channels shared one time base. The public monitoring page shows both installations and the remote acquisition system.[11] Operational side cameras supplied approximately 10-min daylight snapshots for station checks. The fixed Alpine analysis camera recorded at nominal 30-min cadence. Related outdoor platforms, multi-year analyses and sunlight-excited imaging provide complementary field diagnostics.[3, 4, 5, 6]

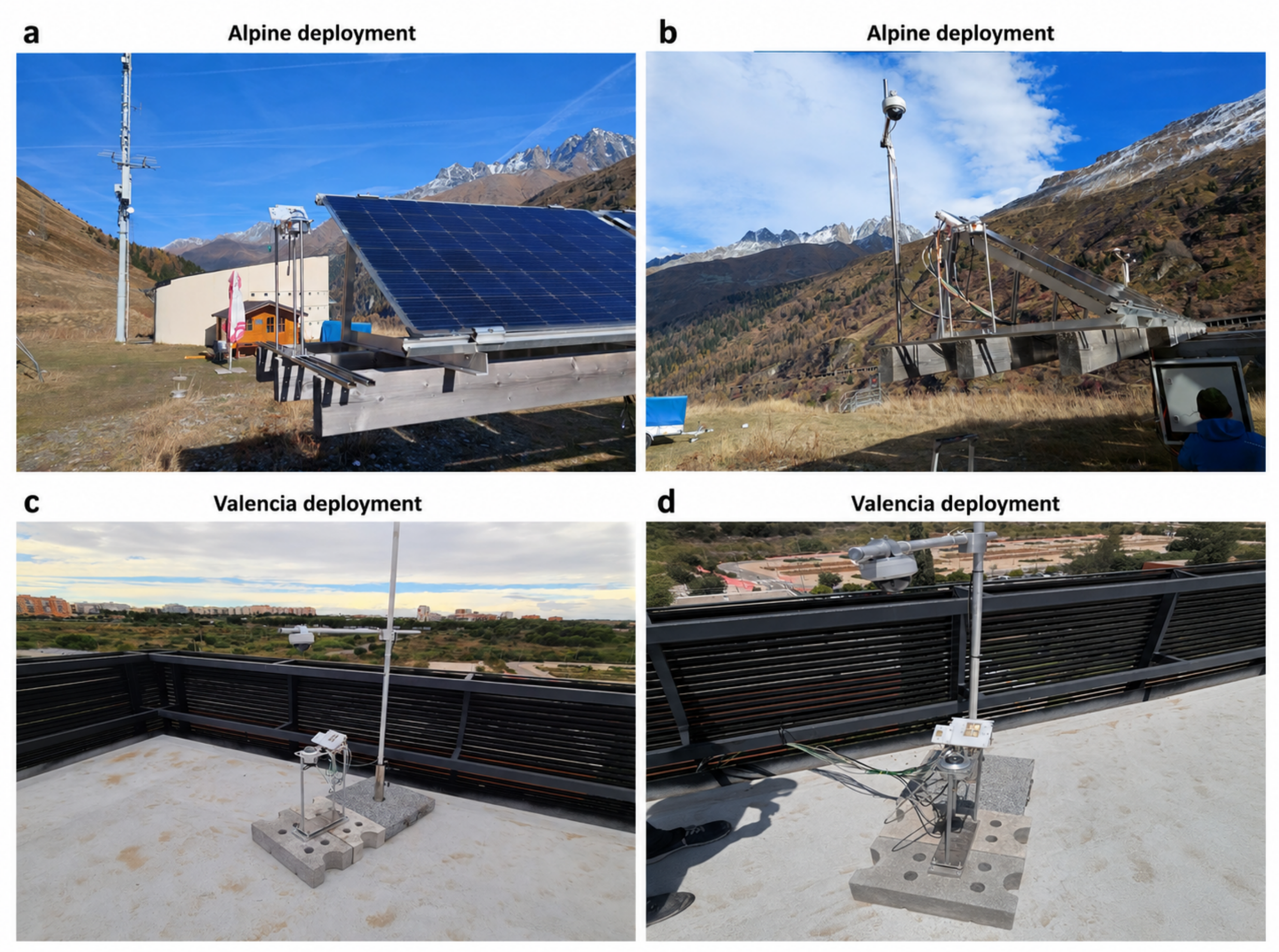

Figure S3. Paired outdoor deployments. a,b, Alpine platform at Lac des Toules / Grand-St-Bernard. c,d, Valencia platform at Paterna.

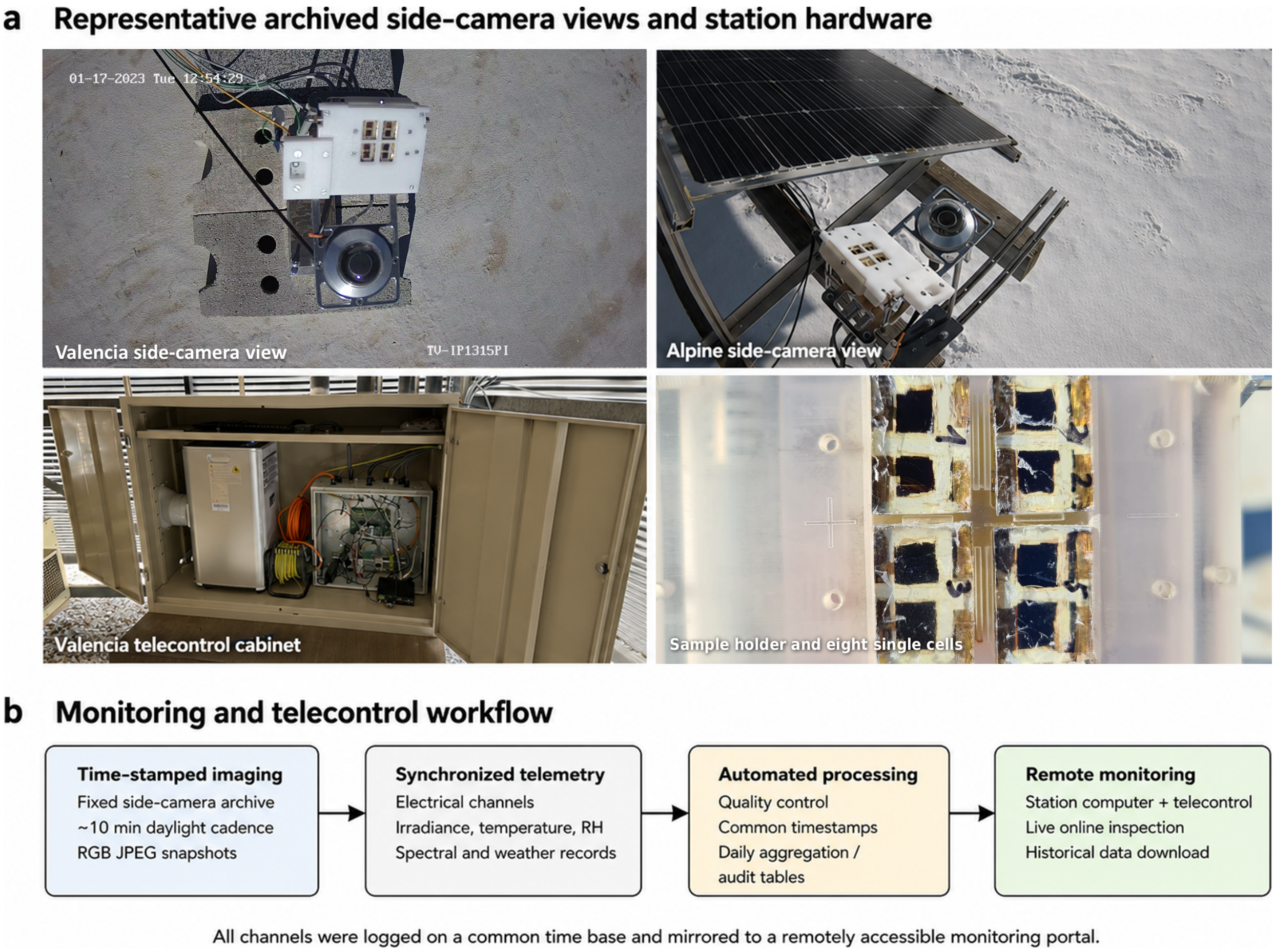

Figure S4. Camera views, station hardware and monitoring workflow. a, Valencia and Alpine side-camera views, the Valencia telecontrol cabinet and the eight-cell holder. b, Common path from time-stamped imaging and telemetry to quality control, daily aggregation and remote monitoring.

**Quantity**	**Value**
Archive JPEG files	18,965
Readable images with embeddings	18,939
Unreadable images	26
Wetness inference-usable images	10,067
Effective device-plane ROI sampling	0.25 mm
Device/reference ROI overlap	None; regions fixed and non-overlapping
Usable fraction	53.15%
Archive dates	400
Dates with usable wetness	395
Median usable images/day	25
Median represented coverage/day	12.50 h
5th–95th percentile coverage	10.0–16.50 h

S3. Manual annotation definitions

S3.1 Original visible environmental labels

- Direct-beam evidence (0–3): strength of clearly directional solar illumination.
- Soft/diffuse-light evidence (0–3): strength of diffuse or low-contrast illumination.

- Ground-snow fraction (0–3): visible snow fraction in the ground reference region.
- Snow or ice on the photovoltaic panel (0–3).
- Snow, ice or water on the device-sensor region (0–3).

The original manual set contained 135 environment-valid images across 82 date or episode groups. These five labels were retained after blocked development and later audited on the frozen 52-image hidden audit set.

S3.2 Revised device-area wetness/thaw label

Score	Criterion
0	Measured-cell holder and immediately adjacent support are clearly dry.
1	Possible or slight wetness, weak sheen or early thaw on the device area.
2	Clearly wet, melting, slushy or runoff on a substantial part of the device area.
3	Extensive water, runoff or active thaw affecting most of the device area.
Blank	Not judgeable because opaque snow, darkness, blur or camera obstruction hides the device area.

Wet grass, distant melting snow and snow cover alone were not scored as device-area liquid wetness.

S3.3 Camera/dome obstruction

A binary QC flag was used: 0 when the measured-device region remained reliably interpretable and 1 when droplets, frost, snow, condensation, haze or foreground occlusion materially prevented interpretation. This field was used only for exclusion and was not treated as an environmental stressor.

S4. Alpine peer-reference and chromatic-replay provenance audits

S4.1 Fixed C1/C3 peer reference and absolute reversibility control

The deposited health trajectories were re-audited against the daily electrical source table. They are reconstructed to numerical precision by a fixed C1/C3 same-day reference, not by a dynamic pool. For C2, C4, C5, C7 and C8 the denominator is the same-day median of C1 and C3. C1 and C3 use leave-self-out referencing to the other control. C4 and all terminal cells therefore never enter the denominator, so the reference composition does not change as cells fail. C1 and C3 were identified retrospectively as the two full-campaign electrical survivors; the peer-reference analysis is consequently retrospective rather than a prospectively designated control rule.

Peer normalisation cannot identify degradation common to both controls. We therefore retained raw plane-of-array-normalised responsivity as an independent reversibility check. Using the same windows as Fig. 2d and a seven-day rolling median, both controls recover from the winter depression to at least their autumn level.

Table S1. Absolute control recovery used to identify the dominant winter common mode as reversible.

Cell	Autumn median	Winter median	Spring median	Spring / autumn
C1	1.118	0.764	1.193	106.8%
C3	1.113	0.627	1.214	109.1%

Values are seven-day-median raw responsivity normalised to each cell's autumn baseline. Windows are 5 October–5 November 2023 (autumn), 1 December 2023–15 February 2024 (winter) and 20 April–10 June 2024 (spring). These controls establish reversibility of the dominant winter excursion, not absence of every possible degradation component shared by C1 and C3.

The terminal-collapse conclusion was also recomputed under four alternatives: C1-only referencing, C3-only referencing, a leave-one-out peer-median stress test and raw responsivity. The orders-of-magnitude C2, C5 and C8 collapses and the weaker C7 mixed-mode phenotype are preserved.

Table S2. Terminal response at the frozen marker under reference alternatives.

Cell	Frozen marker	Primary event / baseline	Range across alternatives	Interpretation
C7	16 Nov 2023	0.163	0.100–0.184	Mixed-mode loss remains distinct.
C2	31 Jan 2024	1.19×10^{-3}	$1.17–2.57 \times 10^{-3}$	Terminal collapse preserved.
C5	8 Mar 2024	4.81×10^{-3}	$3.34–5.87 \times 10^{-3}$	Terminal collapse preserved.
C8	27 May 2024	5.01×10^{-5}	$3.33–6.28 \times 10^{-5}$	Terminal collapse preserved.

S4.2 Terminal-marker definition checks

The submitted terminal dates are retrospective markers held fixed during the final Alpine optical replay. They were not prospectively registered. As marker-definition checks, electrical markers were re-derived without changing or refitting the optical detector. Seven- and fourteen-day rolling health medians crossing $H < 0.30$ with 14 recorded days of persistence each retained the 3/4 historical correspondence and the same four other device-window changepoints. The unsmoothed daily-persistent marker gave 1/4 under the historical 1–14 d optical-first matching rule.

Table S3. Terminal-definition checks without optical refitting.

Electrical marker rule	Matched / 4	Other changes	Median optical-first offset	Offset range
Frozen retrospective markers	3	4	4 d	1–6 d
7-d median, $H < 0.30$, 14-d persistence	3	4	3 d	1–6 d
14-d median, $H < 0.30$, 14-d persistence	3	4	6 d	4–9 d
Daily $H < 0.30$, 14 recorded days	1	6	3 d	3 d

For the daily-persistent definition, only C5/C6 qualifies under the historical 1–14 d optical-first rule: C2 is coincident, C5/C6 is optical-first by 3 d, C8 is electrical-first by 2 d and C7 is missed. The non-failing C3/C4 window also changes on 2 March, the same date as C5/C6. Both rolling-median definitions reproduce 3/4, but

the daily-persistent result shows that the correspondence depends on the electrical marker.

For C2, the frozen retrospective marker is 31 January. The trailing 14-day median first crosses $H < 0.30$ on 2 February and confirms 14 recorded days of persistence on 15 February.

Table S4. Rule-derived electrical-marker dates and confirmation dates.

Cell	Median window	First crossing	Persistence confirmed
C2	1 d	27 Jan 2024	9 Feb 2024
C5	1 d	5 Mar 2024	18 Mar 2024
C7	1 d	16 Nov 2023	29 Nov 2023
C8	1 d	24 May 2024	6 Jun 2024
C2	7 d	30 Jan 2024	12 Feb 2024
C5	7 d	8 Mar 2024	21 Mar 2024
C7	7 d	14 Nov 2023	27 Nov 2023
C8	7 d	27 May 2024	9 Jun 2024
C2	14 d	2 Feb 2024	15 Feb 2024
C5	14 d	11 Mar 2024	24 Mar 2024
C7	14 d	17 Nov 2023	30 Nov 2023
C8	14 d	30 May 2024	12 Jun 2024

S4.3 Dated Alpine analysis-lock chronology

The provenance audit distinguishes retrospective discovery from later analysis lock. The Alpine chromatic detector was not prospectively preregistered before the field outcomes occurred. The full rule was, however, locked before the final replay and no post-lock parameter search was performed.

Table S5. Recovered dated provenance for the Alpine optical analysis.

Date	Stage	Verified status
20 Jun 2024	End of powered Alpine record	Field outcomes complete; this precedes every recovered analysis-lock document.
17 Jul 2026	Evidence-bounded core revision	Chromatic/electrical alignment already documented; therefore Alpine discovery was retrospective.
19 Jul 2026	Provenance/replay lock	Complete detector and matching specification fixed: visible/day gate, port-referenced daily b^*, interpolation ≤ 7 d, PELT–RBF penalty 8, minimum segment 10 d, and one-to-one same-window matching 1–14 d before the fixed electrical marker. Complete replay: 3 matched device-window changepoints, 4 other device-window changes and 2 reference-port control breaks. No post-lock parameter or matching search.
Later validation	Independent Paterna replay	Optical bundle checksum-frozen before electrical-onset unblinding; this has stronger outcome-blind provenance than the Alpine discovery analysis.

S4.4 Analysis-locked Alpine chromatic correspondence characteristics

Table S6. Analysis-locked retrospective chromatic correspondence characteristics.

Quantity	Analysis-locked result
Terminal transitions	4
Matched terminal markers	3

Quantity	Analysis-locked result
Frozen-marker correspondence	0.75; exact binomial 95% CI 0.194–0.994
Optical-first offsets to frozen markers	1, 4 and 6 d; median 4 d
Illumination-conditioned miss	C7 during low-light winter onset
Monitored exposure	4 windows × 265 d = 2.902 window-years
Device-window changepoints	7
Matched device-window changepoints	3
Other device-window changepoints	4
Other changepoints/window-year	1.378; exact Poisson 95% CI 0.376–3.529
Matched fraction of device-window changepoints	0.429
Reference-port control breaks	2; reported separately

The complete denominator was regenerated under the analysis-locked retrospective daily chromatic configuration: daily median b^*(window) - b^*(reference port), image-level visible-light gate, interpolation limited to 7 days, PELT-rbf, penalty 8 and minimum segment size 10 days. A terminal match required a changepoint in the same mapped window 1–14 days before a terminal transition, with one-to-one nearest-following assignment. Across four windows and 265 calendar days per window (2.902 window-years), seven device-window changepoints comprised three matched device-window changepoints and four other device-window changes. The reference-port control contained two additional breaks and was not included in the device-window other-change rate.

Series/window	Cells	Break date	Classification	Matched terminal	Optical-first offset (d)
`reference_port`	none	2023-12-18	`reference_control_break`		
`reference_port`	none	2024-03-17	`reference_control_break`		
`win_BL`	C1;C2	2024-01-27	`true_positive_alarm`	C2	4
`win_BR`	C7;C8	2024-05-26	`true_positive_alarm`	C8	1
`win_TL`	C3;C4	2024-03-02	`false_positive_alarm`		
`win_TL`	C3;C4	2024-05-11	`false_positive_alarm`		
`win_TR`	C5;C6	2023-10-24	`false_positive_alarm`		
`win_TR`	C5;C6	2024-02-01	`false_positive_alarm`		
`win_TR`	C5;C6	2024-03-02	`true_positive_alarm`	C5	6

The machine-readable classification strings `true_positive_alarm` and `false_positive_alarm` are retained verbatim from the frozen provenance manifest. In the manuscript and SI prose, these entries are described as matched and other device-window changepoints.

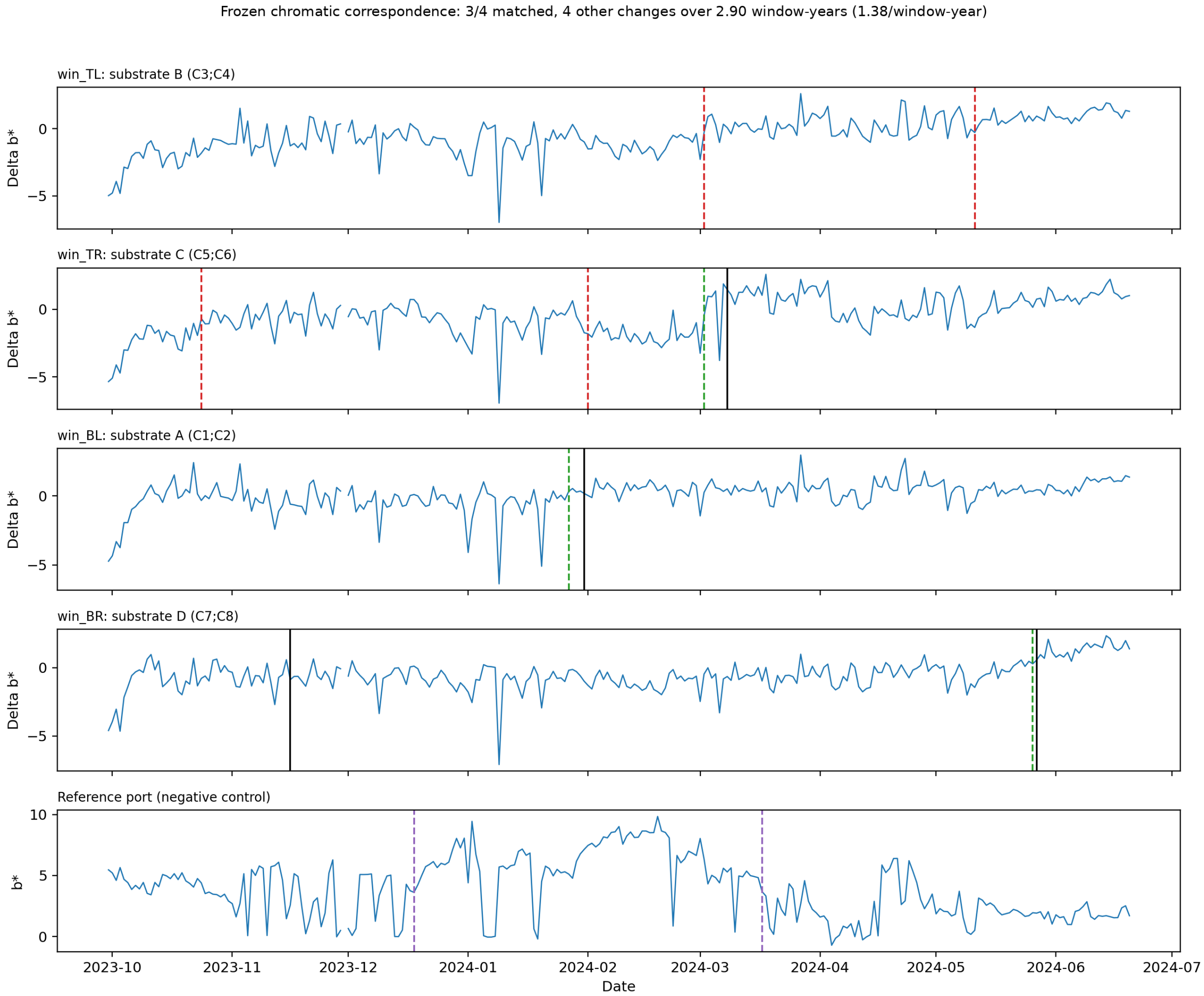

Figure S5. Complete analysis-locked retrospective chromatic correspondence denominator. Reference-port breaks are negative-control changes and are excluded from the device-window other-change rate.

S5. Endpoint XRD mapping and provenance requirements

Sample	PbI_2-window peak 2theta	Perovskite-window peak 2theta	Local peak-height ratio
Sample_1	12.739	14.236	51.90
Sample_2	12.739	14.183	610.67
Sample_3	12.686	14.131	53.89
Sample_4	12.765	13.999	259.07
Sample_5	12.739	13.921	425.43
Sample_6	12.660	14.183	15.11
Sample_7	12.634	13.868	2.25
Sample_8	12.923	14.262	4.60

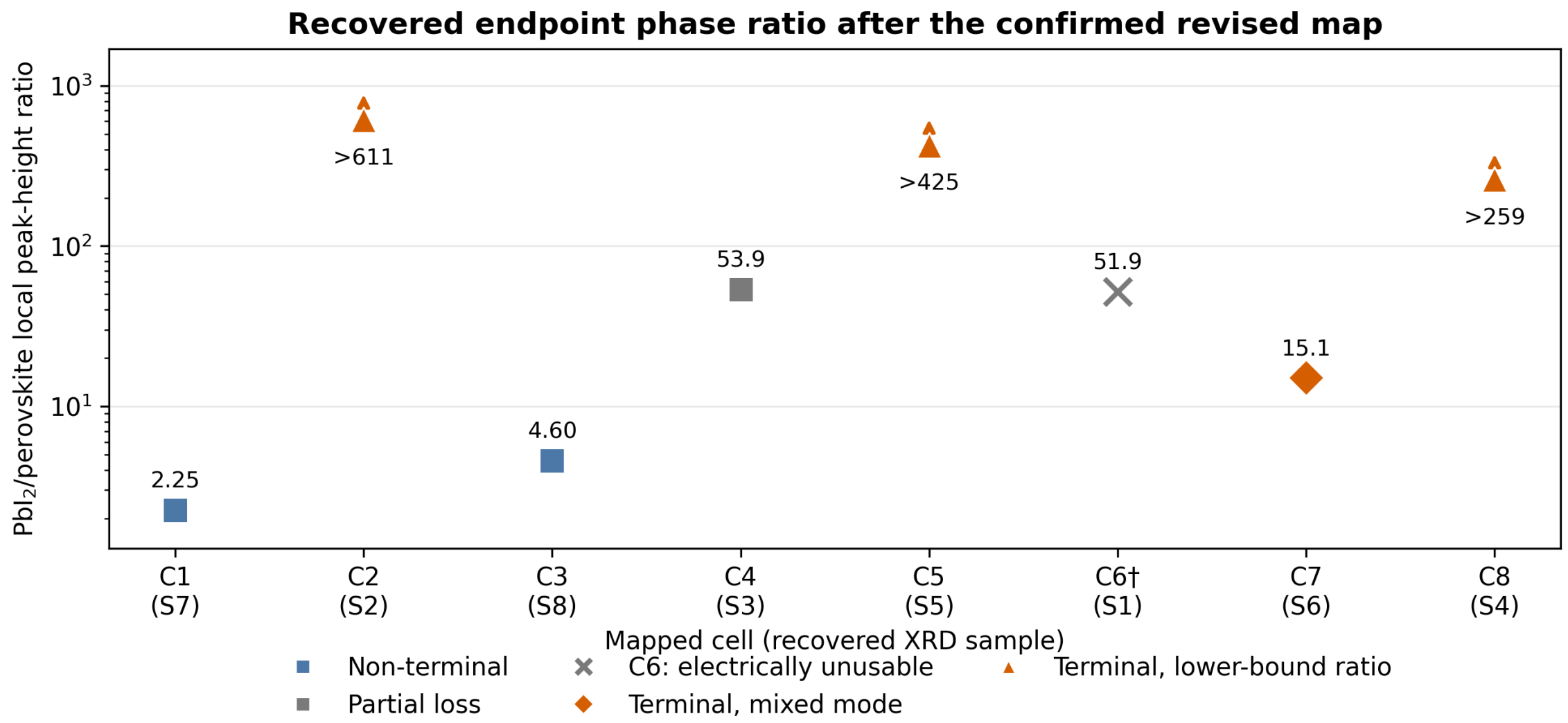

Figure S6. Cell-resolved endpoint PbI_2/perovskite local peak-height ratios after the confirmed revised map. C6 is marked separately because it was electrically unusable from 2 October 2023 and excluded from survivor-referenced health and terminal-event analyses, while its recovered endpoint XRD remained valid. Upward arrows mark lower-bound ratios where the perovskite reflection approached the local background. Ratios are descriptive, not quantitative phase fractions.

C1 and C3 retain the strongest perovskite reflections. For C2, C5 and C8, the remaining perovskite reflection approached the local background, so the reported PbI_2/perovskite ratios are lower bounds rather than finite phase-fraction estimates. C7 retains a clearly resolved perovskite reflection after mixed-mode terminal failure, supporting a second recovered-state route. Time since failure does not explain this contrast: C7 remained terminal for 228 days before recovery, longer than C2 (152 days), C5 (115 days) and C8 (35 days), yet retained more perovskite. The endpoint does not partition field and post-recovery evolution. Rules A and C remain archived alternatives. **Table S7. Confirmed XRD sample-to-cell mapping and field fate.**

XRD sample	Mapped cell	PbI_2/perovskite peak-height ratio	Frozen field fate
Sample_7	C1	2.25	Right-censored survivor to 2024-06-20
Sample_2	C2	>610.67	Terminal transition 2024-01-31; optically warned
Sample_8	C3	4.60	Right-censored survivor to 2024-06-20
Sample_3	C4	53.89	Persistent partial loss; no frozen terminal transition
Sample_5	C5	>425.43	Terminal transition 2024-03-08; optically warned

XRD sample	Mapped cell	PbI_2/perovskite peak-height ratio	Frozen field fate
Sample_1	C6	51.90	Electrically unusable from 2023-10-02; excluded from survivor-referenced health and terminal-event analyses; no frozen terminal transition
Sample_6	C7	15.11	Terminal transition 2023-11-16; mixed mode
Sample_4	C8	>259.07	Terminal transition 2024-05-27; optically warned

Table S8. Recovery, storage, delamination and endpoint-XRD provenance timeline.

Stage	Date	Conditions
Last powered field measurement	20 June 2024	Frozen field archive.
Recovery and storage	1 July 2024	Recovered and stored in a drybox.
Delamination	20 January 2025	Adhesive tape was used to remove the electrical contact and upper encapsulation layer. Sequential layer removal exposed the perovskite; residual Spiro-OMeTAD may remain. No solvent was used.
XRD	21 January 2025	Endpoint XRD measured after delamination.

Rules A and C remain archived alternatives. Rule B required Samples 7 and 8 to map to survivors C1 and C3; Rule C covered any other complete mapping. The confirmed revised map releases Rule B.

Rule	Mapping condition	Interpretation	Limit
A	All Sample_i map to C_i	Residual perovskite does not order with survival. Residual perovskite in C7 is inconsistent with complete absorber conversion as its sole electrical failure route and is compatible with its mixed-mode electrical phenotype. Extensive survivor-endpoint decomposition indicates a substantial post-recovery contribution, without quantifying field versus post-recovery fractions.	The data do not assign decomposition to storage or recover C7 field-state phase fractions.

Rule	Mapping condition	Interpretation	Limit
B	Samples 7 and 8 map to survivors C1 and C3	Residual phase content orders with field fate.	Endpoint PL/PLQY does not reconstruct the historical field state or initiating mechanism.
C	Any other complete mapping	Residual phase content does not order with field fate. Endpoint patterns cannot discriminate the in-field failure hierarchy; heterogeneous failure routes and post-recovery evolution remain inseparable, and the endpoint-only interpretation is retained.	A non-ordered map does not establish storage dominance.
Observed outcome	Confirmed revised map; Samples 7 and 8 correspond to survivors C1 and C3	Rule B released: C1 and C3 retain the strongest perovskite reflections; C2, C5 and C8 are PbI_2-rich lower-bound endpoints; C7 retains perovskite after mixed-mode failure, supporting a second route.	The endpoint does not yield field-state phase fractions or reconstruct the initiating sequence.

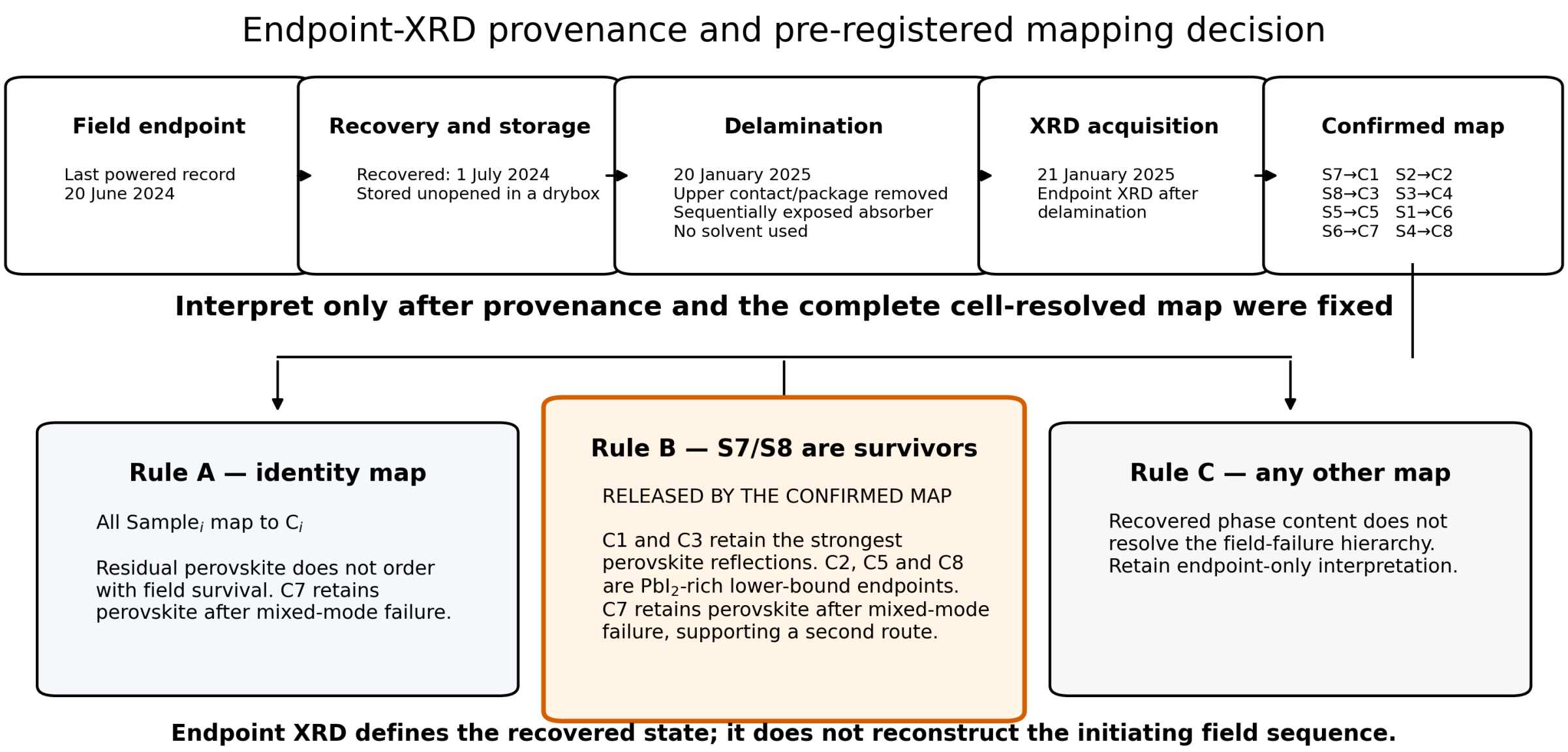

Figure S7. Endpoint-XRD provenance and pre-registered mapping decision. Recovery, drybox storage, delamination, XRD acquisition and the confirmed revised map precede the Rule-B interpretation.

S6. Retrospective series-string comparison using frozen optical dates

The decision-value analysis used the frozen optical changepoint dates, frozen terminal dates, the preclassified C6 unusable date and the existing electrical archive. We therefore constructed an eight-position series string and stated the calculation as an engineering approximation. The active area was fixed at 0.16 cm^2 and independently verified from the MPP identity P = |I|V/A. Valid JV scans were joined to the nearest common-time MPP point within 20 min. Cell-specific robust Jmp/J_{sc} and Vmp/V_{oc} ratios were rescaled so that their product reproduced the median measured FF. C6 had become electrically unusable on 2 October 2023 and was excluded from the survivor-referenced health and terminal-event analyses; for the string reconstruction it used the global median fallback because it lacked a valid cell-specific calibration set. At every common-time MPP record, J_{sc} and V_{oc} were reconstructed and an effective ideal-diode curve was selected to reproduce the frozen FF. String maximum power was max[J sum_i Vi(J)], restricted to the weakest active J_{sc}, with ideal zero-drop bypass. Only GHI >= 20 W m^{-2} was integrated, and gaps longer than 1.01 h were not bridged. All module-area energy densities are therefore divided by eight, the nominal number of string positions; actual string energy is obtained by multiplying the single-cell-area density by 0.16 cm^2.

Both string policies treated C6 as unavailable and bypassed it from 2 October 2023; this date was not classified as a frozen terminal transition. The terminal-date policy then bypassed C7 on 16 November 2023, C2 on 31 January 2024, C5 on 8 March 2024 and C8 on 27 May 2024. The optical-date policy changed only the three matched events, bypassing C2, C5 and C8 on 27 January, 2 March and 26 May, respectively. Event value was integrated over the half-open optical-date-to-terminal interval. The unmatched-change penalty deliberately bypassed the stronger still-active cell in each unmatched two-cell window for one day. This is a conservative energy-only scenario; labour, inspection, switching loss, bypass-diode voltage drop and hardware reliability were not quantified. Two distinct ratios are used and should not be conflated. The 4.17% quoted in the main text is the net relative gain: net recovered energy divided by the total campaign energy the same eight-position string delivered under the terminal-date bypass policy. The 3.98–4.38% band in Fig. S9 is a reconstruction sensitivity on gross campaign value. The event-level terminal-policy entries in Table S9 are optical-date-to-terminal interval energies, not the campaign denominator. An independent no-look-ahead audit froze calibration before the earliest matched device-window changepoint and localised cells using only trailing 48-h MPP data. Additional sensitivities imposed 0–3 d action delay and 0–0.60 V drop per bypassed position.

Table S9. Event-level series-string decision value under the primary reconstruction.

Event	Optical-first offset (d)	Terminal policy (kWh m^{-2} nominal string area)	Optical-date policy (kWh m^{-2} nominal string area)	Recovered (kWh m^{-2})	Actual recovered (mWh)
C2	4	0.021	0.272	0.251	32.1
C5	6	0.047	0.326	0.278	35.6
C8	1	0.000	0.058	0.058	7.5

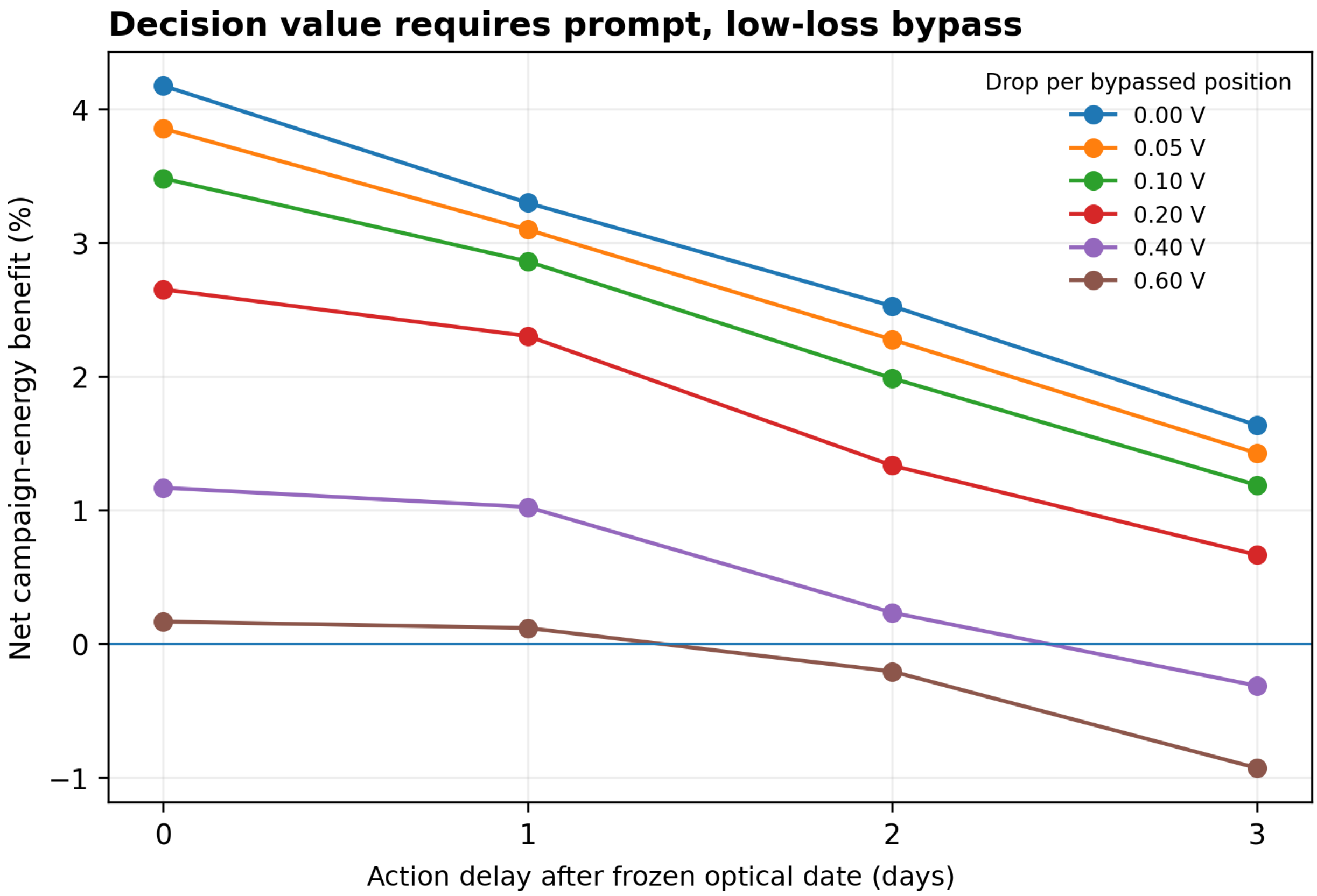

Figure S8. Delay and bypass-loss sensitivity. Net benefit falls with switching delay and voltage drop; a 3-day delay at 0.40 V per bypassed position removes the benefit.

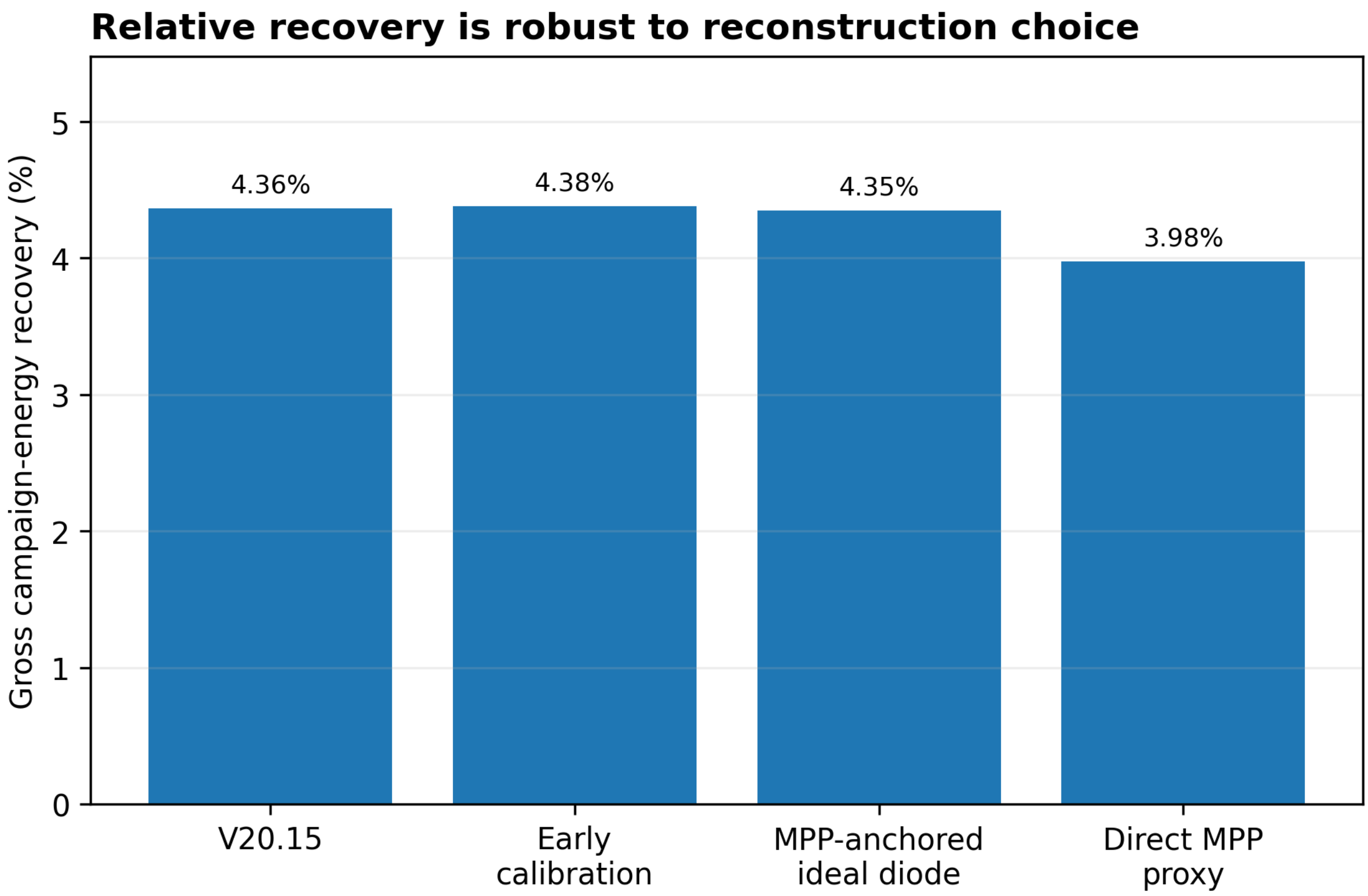

Figure S9. Reconstruction sensitivity. Four curve reconstructions retain 3.98–4.38% of gross campaign value. This gross-value band is a reconstruction sensitivity and is distinct from the 4.17% net relative gain quoted in the main text; the direct MPP proxy gives the lowest absolute recovery.

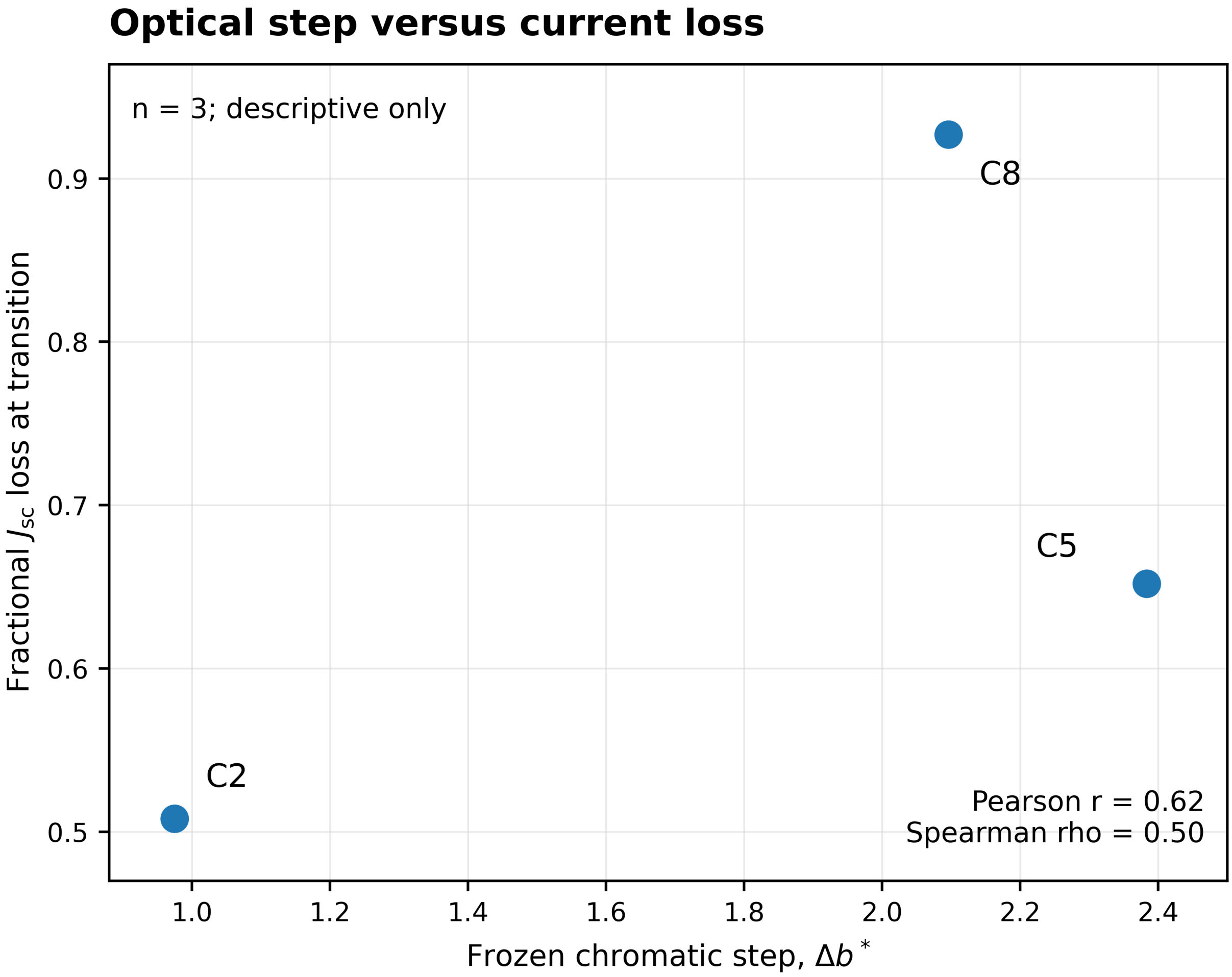

Figure S10. Chromatic step magnitude versus fractional J_{sc} loss for C2, C5 and C8 (n = 3; Pearson r = 0.62, Spearman ρ = 0.50).

The analysis values frozen decisions retrospectively; it does not test a field module, switch controller or maintenance economics. The chromatic detector operates at window level. Trailing 48-h electrical data selected C2, C5 and C8 at the three matched optical changes without future outcome information. After correction of the area normalisation, gross recovery is 0.588 kWh m^{-2} of nominal eight-cell active area and net recovery is 0.562 kWh m^{-2}, equivalent to 75.2 and 72.0 mWh for the hypothetical laboratory string. Curve-reconstruction sensitivities retain 3.98–4.38% gross campaign value.

S7. Original five-label frozen audit

Label	n	MAE	Spearman	QWK	Presence AUROC	Role
Direct beam	52	0.475	0.579	0.610	0.536	Archive context
Diffuse light	52	0.435	0.702	0.611	0.827	Archive context
Ground snow	52	0.477	0.709	0.764	0.900	Archive context
PV-panel snow/ice	52	0.356	0.494	0.553	0.914	Archive context

Label	n	MAE	Spearman	QWK	Presence AUROC	Role
Device-region snow/ice/water	52	0.663	0.561	0.497	0.875	Archive context

The direct-beam label was retained as an ordinal score rather than as a binary presence classifier because its frozen ordinal agreement was acceptable while its binary AUROC was weak. All five labels were used as visible context rather than chemical-state outputs.

S8. Wetness model development and frozen hidden evaluation

The revised training campaign produced 253 fully annotated images. Six obstruction-positive images were excluded, leaving 247 images across 169 calendar dates. Image predictors comprised 512 frozen pretrained embeddings and 27 auxiliary camera features. All 247 training images and all 48 hidden images matched the archive feature table.

Development used five-fold date-blocked cross-validation, matching the time-aware evaluation required for autocorrelated outdoor records and related machine-learning field analyses.[3, 10] Class weighting was applied during training, and the continuous ensemble score was clipped to 0–3. Model selection, preprocessing and the hidden predictions were frozen before manual annotation of the hidden set. The hidden manifest contained 48 images across 37 dates. Five images were excluded by the manual obstruction flag, leaving 43 evaluated images across 36 represented dates. The complete 48-image manifest and the 43-row evaluated prediction table are included as machine-readable CSV files in the accompanying Supporting Data and Code package.

Evaluation	n images	n dates	MAE	Spearman	QWK	Presence AUROC	Exact class accuracy
Date-blocked develop-ment	247	169	0.518	0.846	0.817	0.944	0.571
Frozen hidden test	43	36	0.344	0.899	0.877	0.969	0.698

Table S10. Frozen hidden wetness-test predictions for the 43 unobstructed evaluated images. The continuous frozen score is the primary prediction; the rounded class is descriptive. The complete 48-image manifest, including the five obstruction-positive exclusions, is included as a machine-readable CSV in the accompanying Supporting Data and Code package.

Hidden ID	Archive ID	Timestamp	Manual class	Frozen score	Rounded class	Obstruction
H2-0001	A018848	2024-06-18 13:19	0	0.000	0	0
H2-0002	A002163	2023-07-05 08:46	0	0.232	0	0
H2-0003	A002211	2023-07-06 08:46	1	0.000	0	0
H2-0004	A002641	2023-07-15 07:47	0	0.367	0	0
H2-0005	A003231	2023-07-27 14:47	0	0.000	0	0
H2-0006	A004758	2023-08-28 10:19	3	1.413	1	0
H2-0007	A018610	2024-06-13 14:19	0	0.072	0	0
H2-0009	A003892	2023-08-10 09:18	0	0.619	1	0
H2-0010	A004579	2023-08-24 16:49	0	0.000	0	0
H2-0011	A016150	2024-04-23 08:10	3	3.000	3	0
H2-0012	A002269	2023-07-07 13:46	0	0.169	0	0

Hidden ID	Archive ID	Timestamp	Manual class	Frozen score	Rounded class	Obstruction
H2-0013	A011066	2024-01-08 08:49	3	2.836	3	0
H2-0014	A005349	2023-09-09 17:49	0	0.000	0	0
H2-0016	A005532	2023-09-13 13:20	3	2.268	2	0
H2-0017	A009341	2023-12-03 10:18	3	1.905	2	0
H2-0018	A018413	2024-06-09 11:49	2	1.023	1	0
H2-0019	A010248	2023-12-22 07:49	1	1.188	1	0
H2-0020	A010969	2024-01-06 08:19	3	3.000	3	0
H2-0021	A008082	2023-11-05 15:17	3	2.229	2	0
H2-0022	A016972	2024-05-10 11:11	0	0.525	1	0
H2-0023	A007024	2023-10-14 15:16	3	2.327	2	0
H2-0024	A000172	2023-01-05 13:50	0	0.239	0	0
H2-0025	A002285	2023-07-07 21:46	2	1.844	2	0
H2-0026	A002552	2023-07-13 11:17	2	2.191	2	0
H2-0028	A011065	2024-01-08 08:19	3	2.812	3	0
H2-0029	A002787	2023-07-18 08:47	1	0.874	1	0
H2-0030	A018425	2024-06-09 17:49	2	2.110	2	0
H2-0031	A002756	2023-07-17 17:17	0	0.724	1	0
H2-0032	A010249	2023-12-22 08:19	1	1.303	1	0
H2-0033	A004619	2023-08-25 12:49	0	0.247	0	0
H2-0034	A004713	2023-08-27 11:49	2	1.610	2	0
H2-0035	A005544	2023-09-13 19:20	2	2.216	2	0
H2-0036	A000835	2023-01-19 09:20	3	2.800	3	0
H2-0037	A004663	2023-08-26 10:49	0	0.563	1	0
H2-0038	A004343	2023-08-19 18:48	0	0.516	1	0
H2-0039	A000834	2023-01-19 08:50	3	2.735	3	0
H2-0040	A016147	2024-04-23 06:40	3	2.919	3	0
H2-0041	A016776	2024-05-06 09:11	2	2.169	2	0
H2-0042	A016261	2024-04-25 15:40	0	0.000	0	0
H2-0044	A009295	2023-12-02 11:18	3	2.930	3	0
H2-0046	A011641	2024-01-20 08:20	3	3.000	3	0
H2-0047	A009223	2023-11-29 09:48	3	3.000	3	0
H2-0048	A009865	2023-12-14 08:18	3	2.151	2	0

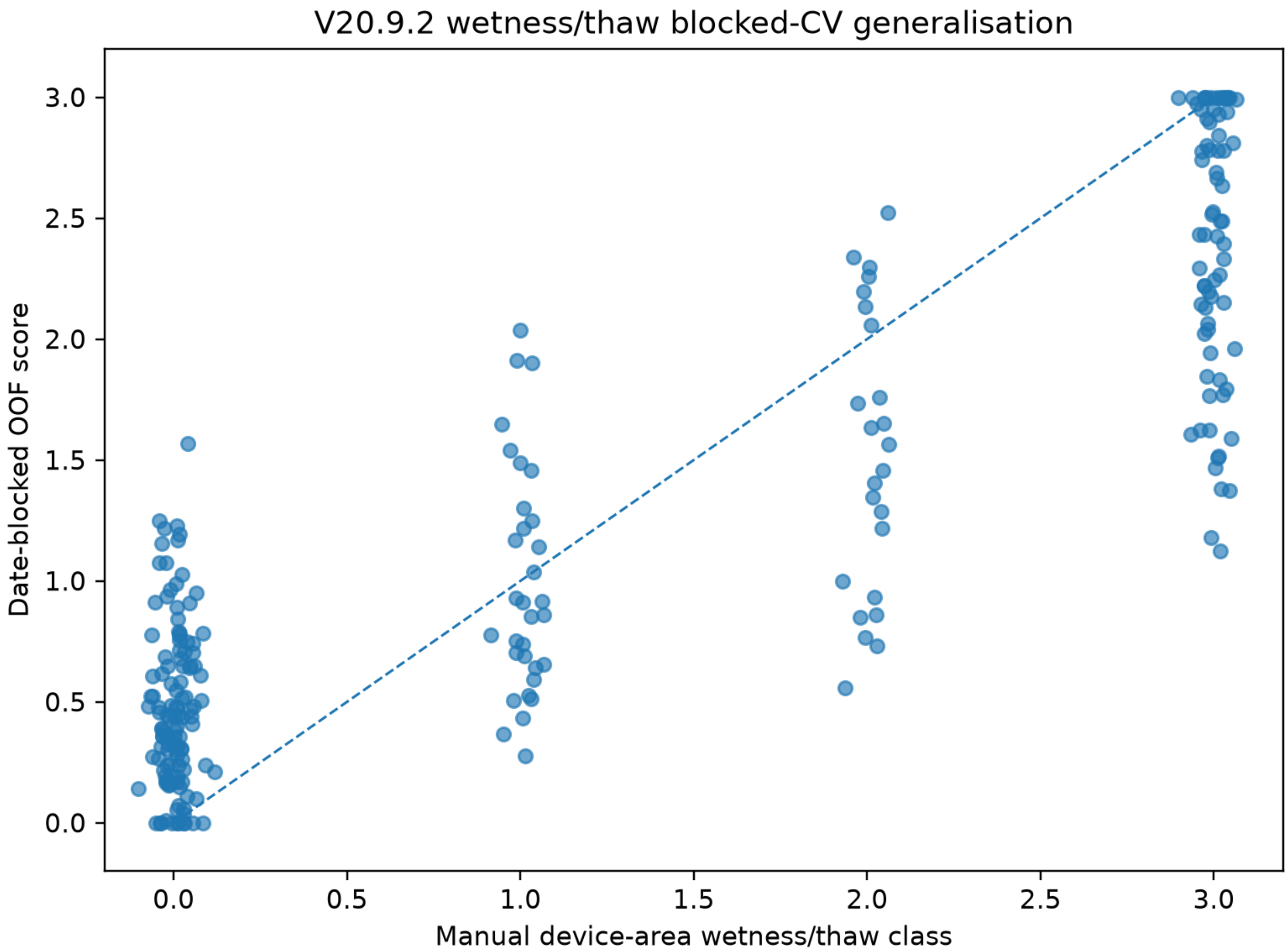

Figure S11. Date-blocked out-of-fold generalisation of the device-area wetness/thaw score. Most errors are between neighbouring ordinal classes.

S9. Archive inference and daily aggregation

The hidden-test decision promoted the ordinal score to archive inference. The frozen model processed all 18,939 readable images, and 10,067 images passed the pre-existing environmental inference gate. The usable-score distribution had mean 0.958, median 0.731, 5th percentile 0 and 95th percentile 2.729.

Daily aggregation used the frozen midpoint/Voronoi local-time support calculated from all archive timestamps, summed only for inference-usable images, with a 3 h per-image cap. This produced a median 12.50 h/day of represented usable coverage. Days without usable images were retained as missing rather than interpolated.

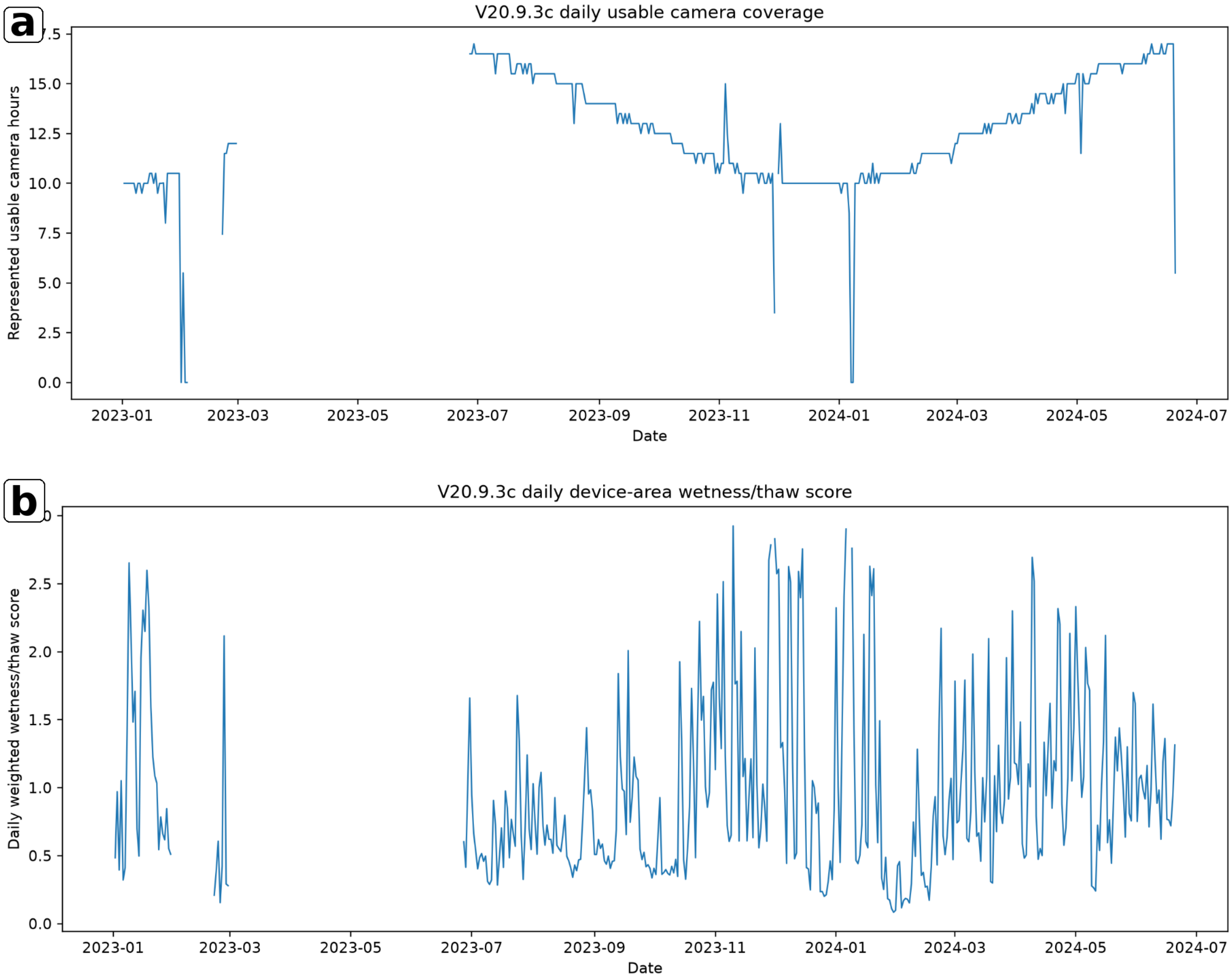

Figure S12. Archive support and wetness inference. a, Corrected daily usable camera coverage. b, Daily device-area wetness/thaw score. Long archive gaps are retained rather than connected by artificial interpolation.

S10. Dependence on existing camera variables

The wetness score was not treated as an independent camera channel because it was derived from the same frozen image embeddings and auxiliary camera outputs. Its strongest daily associations were with the earlier device-region surface proxy and illumination state.

Existing camera variable	n days	Spearman with wetness
Device-region surface mean	395	0.865
Device-region surface maximum	395	0.790
Direct-beam mean	395	-0.761
Device-region presence hours	395	0.725
Direct-beam high hours	395	-0.691
Direct-beam maximum	395	-0.664
Diffuse-light mean	395	0.636
Device-region uncertainty median	395	0.603
Direct-beam presence hours	395	-0.599
PV-panel snow presence hours	395	0.591

S11. Frozen wetness-replacement sensitivity

The primary robustness endpoint, vulnerable-cell outcome, at-risk floor of 0.15, 14-day blocks, five-fold GroupKFold split and RidgeCV model family were imported directly from the primary analysis. The original metrics were reproduced exactly. At 12 independent blocks, the two-sided sign-flip randomisation test is the primary inferential layer; the paired block-mean effect, cluster-bootstrap interval and 80%-power minimum detectable improvement are displayed alongside it. The frozen V20.9.4 paired tests used 50,000 Monte Carlo sign-flip randomisations with $p = (k+1)/(B+1)$, with the random-number generator initialised at seed 21712 (the frozen V20.8 seed 20808 plus 904). Cluster-bootstrap intervals used 10,000 block resamples from the same frozen random-number stream. Discordance is reported explicitly.

Model	Predictors	OOF MAE	OOF R2	OOF Spearman
Baseline	5	0.171	0.483	0.689
Sensor only	15	0.179	0.432	0.663
Sensor + original camera	21	0.179	0.424	0.654
Sensor + wetness replacement	21	0.193	0.316	0.599
Original full	26	0.195	0.328	0.589
Conservative wetness full	25	0.202	0.284	0.587

Comparison	Mean MAE improvement	95% block-bootstrap interval	Sign-flip p	80% MDE
Original camera → wetness replacement	-0.0268	[-0.0660, -0.0001]	0.115	0.0511
Sensor only → wetness replacement	-0.0216	[-0.0468, -0.0009]	0.101	0.0348
Baseline → wetness replacement	-0.0294	[-0.0564, -0.0047]	0.060	0.0393
Original full → conservative wetness full	-0.0058	[-0.0132, 0.0013]	0.162	0.0109
Sensor only → conservative wetness full	-0.0282	[-0.0526, -0.0063]	0.037	0.0345
Baseline → conservative wetness full	-0.0361	[-0.0630, -0.0106]	0.033	0.0391

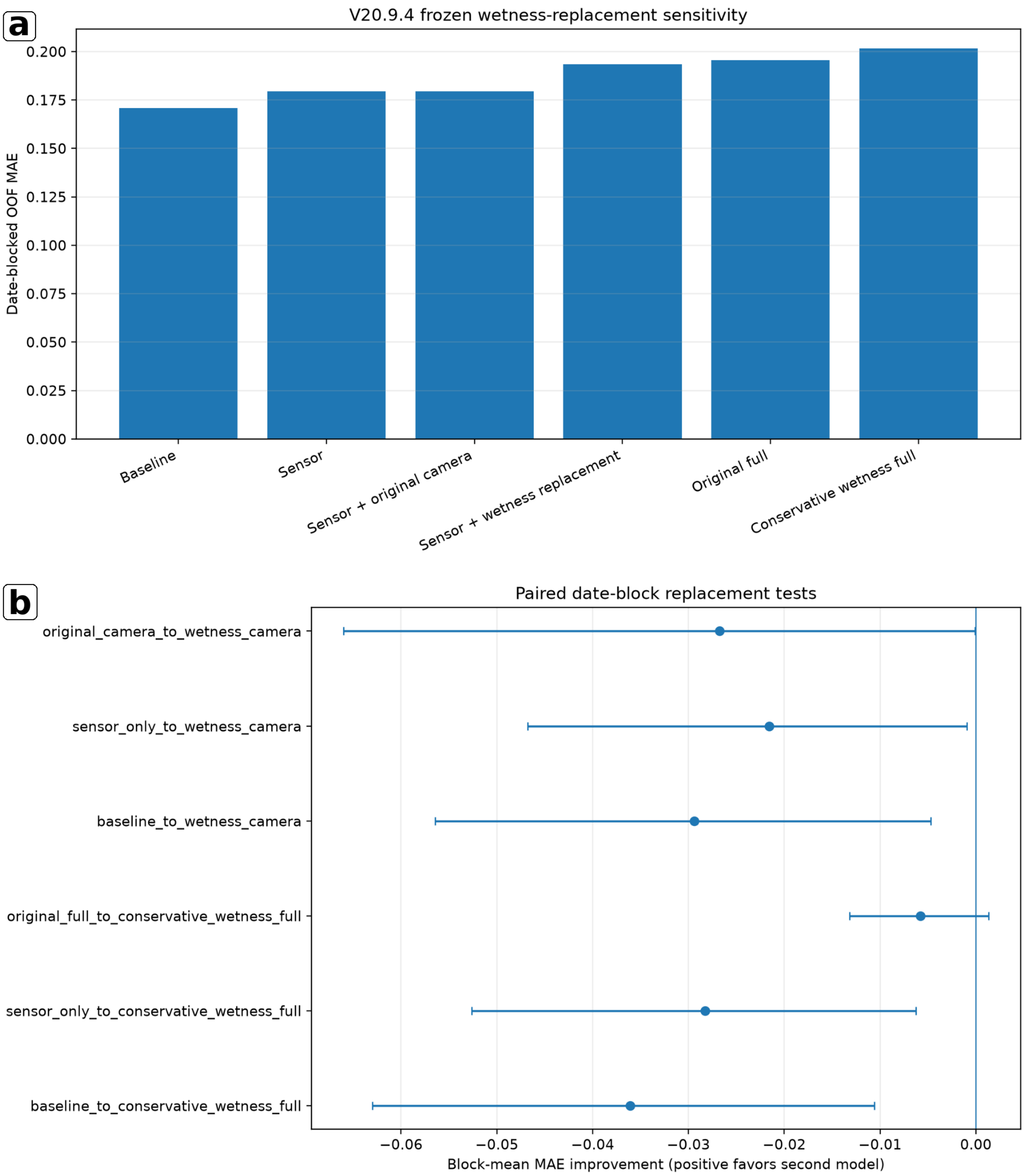

Figure S13. Frozen replacement sensitivity. Positive paired differences would favour the second model; observed differences are non-positive.

S12. Frozen event dates annotated with wetness context

The frozen event and control dates were retained without rematching. The validated wetness score provides descriptive surface-state context. Because event definitions and the wetness model share visible inputs, these contrasts describe co-occurrence rather than causal effects.

Frozen event class	n	Event wetness	Control wetness	Difference	Descriptive 95% CI
Humid diffuse exposure	13	2.14	0.40	1.73	[1.40, 2.06]
Snow + direct beam + UV/POA	8	0.44	1.24	-0.81	[-1.03, -0.61]
Snowmelt + rapid clear heating	4	0.65	1.06	-0.41	[-0.81, -0.01]
Clear thermomechanical cycling	2	0.38	1.49	-1.10	not estimable
Wet surface + freeze–thaw	9	1.73	0.79	0.94	[0.20, 1.68]

S13. Reporting rules

- Use the continuous wetness score as the primary variable; rounded classes are descriptive only.
- Use only images and days passing the frozen quality gate.
- Describe the score as device-area wetness/thaw, not perovskite chemical state.
- Do not add the wetness score beside all source camera variables and call it independent evidence.
- Retain the primary robustness result and the replacement analysis as a frozen replacement sensitivity.
- Do not search new wetness thresholds, compound definitions, event windows, health horizons or model families.
- Report the lack of predictive gain together with the 80%-power minimum detectable improvement.
- Treat event wetness contrasts as environmental-context validation, not causal confirmation.

S14. Development-to-audit label performance with clustered uncertainty

The original five-label audit used 52 images, whereas the independent wetness audit contained 48 images and evaluated 43 after five obstruction exclusions. They are distinct hidden sets. Development confidence intervals were bootstrapped over date/episode groups; hidden intervals were bootstrapped over calendar date.

Campaign	Evaluation	Label	n	groups/dates	QWK [95% CI]	Presence AUROC [95% CI]
Original five labels	Date/episode-blocked development	Direct-beam evidence	135	82	0.797 [0.727, 0.858]	0.914 [0.864, 0.960]
Original five labels	Frozen 52-image hidden audit	Direct-beam evidence	52	39	0.586 [0.347, 0.755]	0.547 [0.306, 0.800]

Campaign	Evaluation	Label	n	groups/dates	QWK [95% CI]	Presence AUROC [95% CI]
Original five labels	Date/episode-blocked development	Diffuse-light evidence	135	82	0.651 [0.546, 0.735]	0.957 [0.907, 0.991]
Original five labels	Frozen 52-image hidden audit	Diffuse-light evidence	52	39	0.585 [0.368, 0.744]	0.818 [0.635, 0.957]
Original five labels	Date/episode-blocked development	Ground-snow fraction	135	82	0.893 [0.843, 0.928]	0.977 [0.954, 0.994]
Original five labels	Frozen 52-image hidden audit	Ground-snow fraction	52	39	0.761 [0.581, 0.868]	0.891 [0.781, 0.968]
Original five labels	Date/episode-blocked development	PV-panel snow/ice	135	82	0.904 [0.859, 0.944]	0.985 [0.964, 1.000]
Original five labels	Frozen 52-image hidden audit	PV-panel snow/ice	52	39	0.532 [0.092, 0.770]	0.911 [0.833, 0.981]
Original five labels	Date/episode-blocked development	Device-region snow/ice/water	135	82	0.763 [0.668, 0.841]	0.954 [0.910, 0.986]
Original five labels	Frozen 52-image hidden audit	Device-region snow/ice/water	52	39	0.497 [0.301, 0.677]	0.869 [0.768, 0.961]
Targeted device-area wetness/thaw	Date-blocked development	Device-area wetness/thaw	247	169	0.817 [0.768, 0.856]	0.944 [0.912, 0.967]
Targeted device-area wetness/thaw	Frozen hidden test: 48 total, 43 unobstructed evaluated	Device-area wetness/thaw	43	36	0.877 [0.771, 0.940]	0.969 [0.900, 1.000]

The clustered intervals quantify finite-set uncertainty; small point-value differences can arise when archive predictions are regenerated. Direct-beam hidden binary AUROC was 0.536 in the official audit, supporting its use as an ordinal context score rather than a binary classifier.

S15. Linked camera-electrical observation-context example

The example was selected using a rule independent of electrical outcome: the first date with mean wetness at least 2.5, at least 10 h usable camera support, valid vulnerable-cell health and more than 21 d from every terminal transition. The selected date was 8 December 2023.

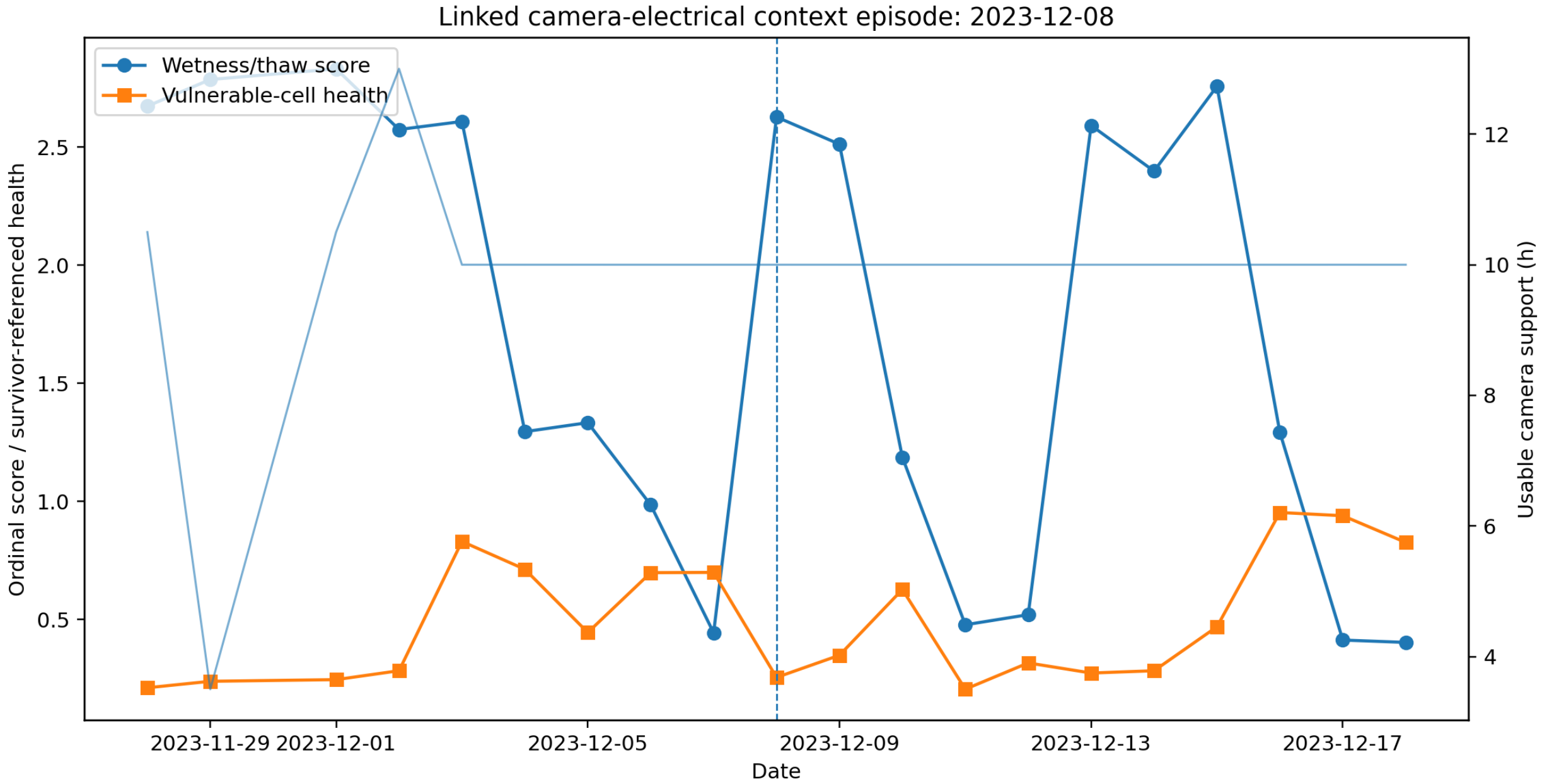

Figure S14. High-wetness context away from terminal transitions. The concurrent health trace remains non-terminal.

S16. Full pairwise concordance matrix for blocked predictive models

Positive paired block-mean delta means the second model has lower MAE. This secondary all-pairs concordance audit is distinct from the frozen six V20.9.4 paired replacement tests in Section S11, which retain their original 50,000-draw Monte Carlo sign-flip p values. For the concordance audit, the 12 stored block-level deltas permit deterministic enumeration of all $2^{12} = 4096$ sign configurations. Cluster-bootstrap intervals are complementary and used 30,000 block resamples with base random seed 2011, incremented by comparison index. At this small block count the bootstrap interval can be optimistic or discordant with the exact test. Therefore the interpretation is robust absence of incremental benefit, never wetness harm.

Table S11. Full pairwise blocked-model concordance with Holm correction.

Comparison: A -> B	Paired delta MAE	Cluster-bootstrap 95% CI	Sign-flip p	Holm p	80% MDE
Baseline -> Sensor only	-0.0078	[-0.0187, 0.0018]	0.1875	0.9702	0.0154
Baseline -> Sensor + original camera	-0.0026	[-0.0223, 0.0191]	0.8228	1.0000	0.0310
Baseline -> Sensor + wetness replacement	-0.0294	[-0.0564, -0.0047]	0.0596	0.7152	0.0393
Baseline -> Original full	-0.0303	[-0.0571, -0.0049]	0.0605	0.7152	0.0394
Baseline -> Conservative wetness full	-0.0361	[-0.0630, -0.0106]	0.0332	0.4648	0.0391
Sensor only -> Sensor + original camera	0.0052	[-0.0099, 0.0229]	0.6021	1.0000	0.0245
Sensor only -> Sensor + wetness replacement	-0.0216	[-0.0468, -0.0009]	0.1006	0.9054	0.0348
Sensor only -> Original full	-0.0224	[-0.0478, -0.0002]	0.1172	0.9224	0.0358
Sensor only -> Conservative wetness full	-0.0282	[-0.0526, -0.0063]	0.0372	0.4836	0.0345

Comparison: A -> B	Paired delta MAE	Cluster-bootstrap 95% CI	Sign-flip p	Holm p	80% MDE
Sensor + original camera -> Sensor + wetness replacement	-0.0268	[-0.0660, -0.0001]	0.1153	0.9224	0.0511
Sensor + original camera -> Original full	-0.0276	[-0.0628, -0.0024]	0.0669	0.7152	0.0469
Sensor + original camera -> Conservative wetness full	-0.0334	[-0.0685, -0.0081]	0.0186	0.2790	0.0464
Sensor + wetness replacement -> Original full	-0.0009	[-0.0115, 0.0081]	0.8730	1.0000	0.0147
Sensor + wetness replacement -> Conservative wetness full	-0.0067	[-0.0167, 0.0023]	0.2236	0.9702	0.0143
Original full -> Conservative wetness full	-0.0058	[-0.0132, 0.0013]	0.1617	0.9702	0.0109

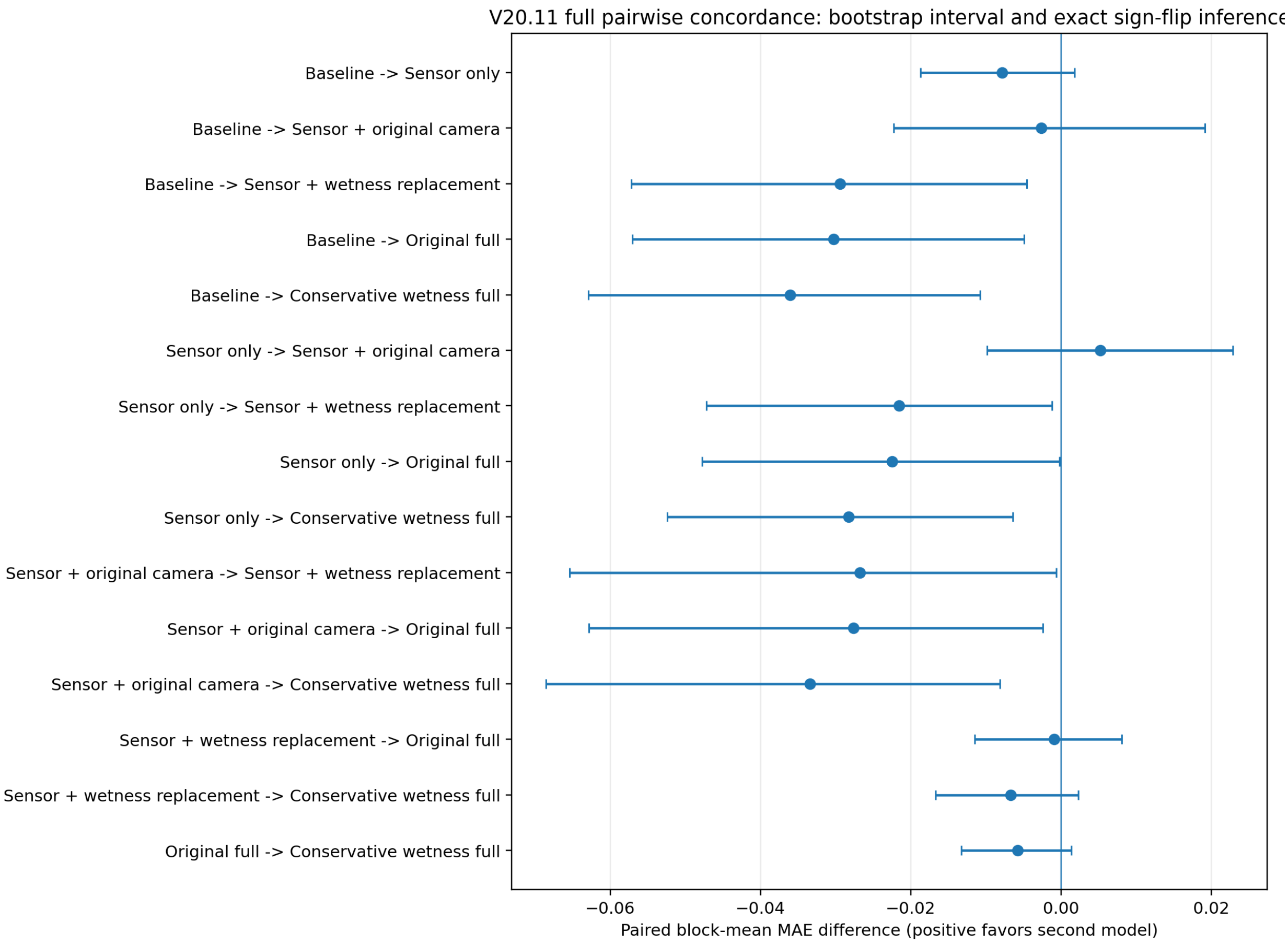

Figure S15. All 15 pairwise model comparisons. Sign-flip randomisation p values provide the primary inference; Holm correction across the 15 stored comparisons gives a minimum adjusted $p = 0.279$, so no contrast remains significant after multiplicity correction.

S17. Daytime limitation and diurnal moisture triangulation

The RGB wetness score is principally a visible daytime surface-state variable because night/infrared frames were excluded. Across 392 dates, morning wetness correlated inversely with the previous-night minimum dew-point margin (Spearman ρ -0.571; date-bootstrap 95% interval -0.635 to -0.499) and positively with

previous-night mean RH (rho 0.536). This is descriptive triangulation, not a new degradation predictor.

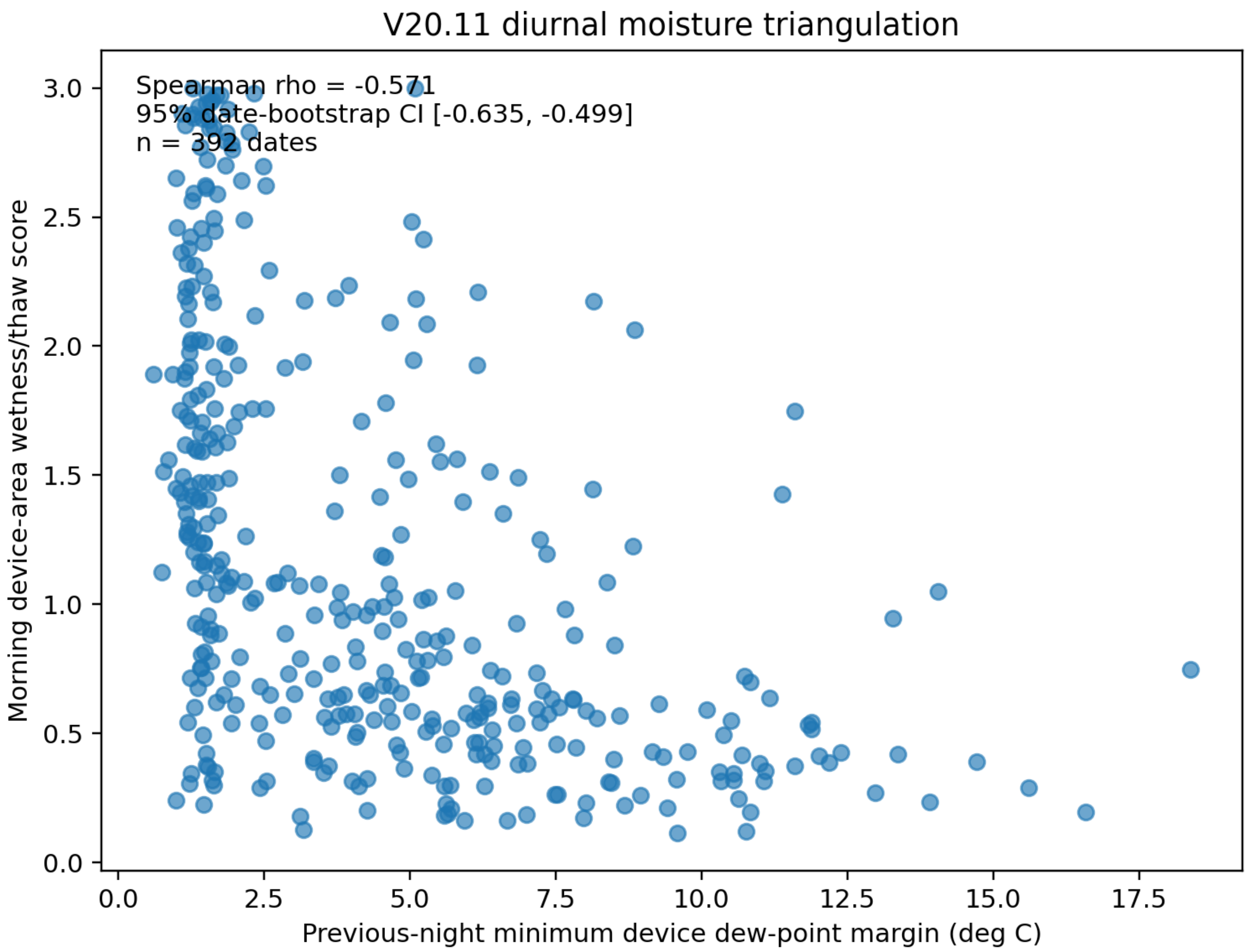

Figure S16. Morning visible wetness/thaw versus the previous-night minimum device dew-point margin. The modalities cover complementary portions of the moisture cycle.

The median 12.50 h/day and 10.0–16.5 h 5th–95th percentile values are midpoint/Voronoi represented observation support. They are not daylight-duration estimates and can exceed alpine winter sun-up hours because each usable observation receives local time support bounded by neighbouring archive timestamps.

S18. Paired environmental exposures and per-cell energy

Identified cell / analysis scope	Integrated per-cell energy (kWh m^{-2})
Alpine C1	6.738
Alpine C3	3.730
Alpine C8	7.797
Alpine C2* (through 31 January)	2.416
Paterna C3 (formal target)	4.177
Paterna C4 (formal target)	10.069

Figure S17 reports the individual-cell integrated yields for the displayed records. The Alpine values are calculated from the common-window cell-energy table; C2 is integrated only to its frozen 31 January terminal marker. The Paterna C3 and C4 values are reproduced from the archived

Valencia daily power-sum source distributed with the Supporting Data and Code package (SHA-256 `f327fc419a776367872e581c51b872a7c53e26cff8029231d4ece10a27ae822a`). Because the available cells differ in selection and record completeness between sites, these per-cell values are not combined into a site-level yield statistic.

For temporal comparison, normalised trajectories use the separate harmonised two-site table: daily cell energy is divided by same-day modelled plane-of-array energy, normalised to the first seven valid common-window days, and smoothed over five days for display only. Physical covariates cover all 66 common dates. Spectral-band comparisons use the 51 dates with valid spectra at both sites; spectral gaps are not filled.

Table S12. Identical-window physical exposure summary.

Site	POA sum (MJ m^{-2})	Mean Tdevice (°C)	Max Tdevice (°C)	Mean night vapour pressure (kPa)	Freeze-thaw days	Spectral coverage
Alpine	638.4	4.9	32.30	0.376	32	66/66
Paterna	813.9	19.6	44.16	0.882	0	51/66

Table S13. Spectral-band energy on 51 paired valid dates.

Site	UV (MJ m^{-2})	VIS (MJ m^{-2})	Red/NIR (MJ m^{-2})	Sub-gap (MJ m^{-2})	Measured bands / POA	UV fraction
Alpine	1.74	119.59	84.45	50.14	0.51	0.68%
Paterna	6.62	242.75	105.43	171.01	0.79	1.26%

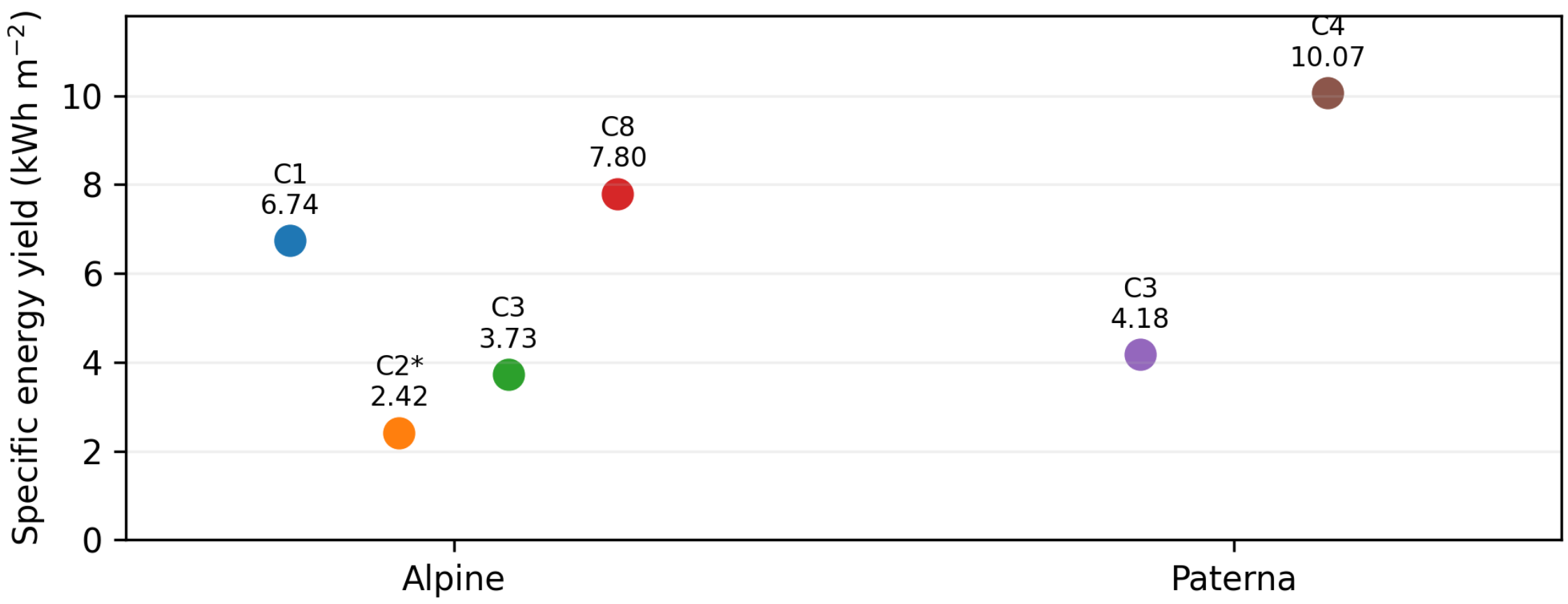

Figure S17. Per-cell integrated yield over the common 66-day window. Alpine C2* is integrated only to its 31 January terminal marker. Paterna C3 and C4 use the archived Valencia daily power-sum source. No site-level aggregate is inferred from these selected cell records.

S19. Outcome-blind Paterna optical transfer test

The second-site analysis tested whether the Alpine optical–electrical timing transferred without retuning a Paterna detector. The STEP05 optical lock named C3 and C4 as the formal electrical target cells before formal electrical-onset unblinding. All eight cell regions were processed under the same frozen optical extraction and

changepoint settings, while only C3/C4 entered the locked transfer classification. The broader Paterna archive contains electrical records for all eight channels; the harmonised 66-day table is used below only as descriptive cohort context.

The image inventory, registration geometry, cell and control regions, image-quality thresholds, daily support rule and changepoint parameters were checksum-frozen before the electrical onset dates were calculated or aligned. The complete window comprised 3,168 readable images from 5 December 2023 to 8 February 2024. The primary extraction retained 833 quality-passed frames; 65 of 66 days met the minimum of four accepted frames. The isolated invalid day, 8 January, was filled only for the optical changepoint sequence by the frozen one-day interpolation rule.

The historical electrical input used at formal unblinding has been recovered with an exact match to the SHA-256 stored in the formal report: `af6482ce5a8ffc9045f6a7569d81e74e8e84296b67bff9615e4cda02da99b1db`. Running the guarded STEP06 script with this input and the archived optical outputs reproduces the C3/C4 onset and match tables: C3 onset on 14 December 2023 and C4 onset on 21 December 2023, with optical changes on 1 and 3 January 2024 (+18 and +13 d). The later harmonised paired-site table has SHA-256 `0c3205aae68ac6d96d5fbe6a2a4d6f62cf7cc521f977f542cf32e55d9e328947`; it is not substituted for the formal unblinding input.

Table S14. Frozen Paterna image and analysis chain.

Stage	Frozen specification or result
Window and inventory	5 December 2023–8 February 2024; 66 calendar days; 3,168/3,168 images readable.
Registration and ROIs	Affine holder registration; eight cell active-area regions; primary and secondary white-holder references; one background control.
Quality calibration	Six neutral frames from 4–5 December; final neutral-frame acceptance 6/6; registration/QC configuration SHA-256 `d18e22be...75e92b`.
Daily extraction	Cell median b^* minus primary-reference median b^*; supporting L^*, a^*, chroma, secondary-reference and dispersion metrics; minimum four accepted frames per day.
Primary support	833 accepted frames; 65 valid days; 8 January contained three accepted frames.
Optical changepoints	PELT–RBF; penalty 8; minimum segment 10 d; no smoothing; isolated one-day linear interpolation; all eight cells processed identically.
Electrical onset	First of three consecutive days below 50% of the first-seven-day median, with at least six of the seven days from onset below threshold; no electrical interpolation.
Transfer match	Optical event from −7 to +2 d relative to onset and not scene-common.
Frozen electrical input	Historical STEP06 input SHA-256 `af6482ce...99b1db`; later harmonised paired-site table SHA-256 `0c3205aa...28947` is a separate input.

Table S15. Paterna target-channel provenance and scope.

Item	Verified status
Optical regions	C1–C8 processed identically under the frozen optical pipeline.
Formal electrical targets	C3 and C4.
Target lock	C3/C4 explicitly named in STEP05 before formal electrical-onset unblinding.
Other Paterna electrical channels	Present in the broader archive and shown below as descriptive context; they did not enter the locked transfer classification.
Selection limitation	Target selection was performance-based rather than random. The formal transfer inference is limited to C3/C4 and is not an eight-cell population estimate.

Item	Verified status
Chronology	C3/C4 designation on 23 June 2026 and formal electrical-onset unblinding on 20 July 2026 are author-reported dates; the recovered lock files establish ordering but do not independently timestamp these milestones.

The frozen electrical criterion placed C3 onset on 14 December and C4 onset on 21 December. PELT detected the C3 optical changepoint on 1 January and the C4 changepoint on 3 January. The corresponding offsets were +18 and +13 d, outside the locked −7 to +2 d window. Neither cell qualified, and the formal classification was null. With a 10-day minimum segment and a 5 December series start, the earliest detectable new segment is 15 December, so only 15–16 December lay inside the C3 matching window. The optical changes were not unique to C3/C4: C7 changed on 23 December, C8 on 24 December, C1/C2 on 28 December, C3 on 1 January, C5/C6 on 2 January and C4 on 3 January; C7 showed a second change on 22 January. Neither scene-control series produced a changepoint.

Table S16. Frozen optical–electrical match result.

Cell	Electrical onset	Optical changepoint	Offset	Frozen result
C3	14 December 2023	1 January 2024	+18 d	Outside match window; non-qualifying.
C4	21 December 2023	3 January 2024	+13 d	Outside match window; non-qualifying.

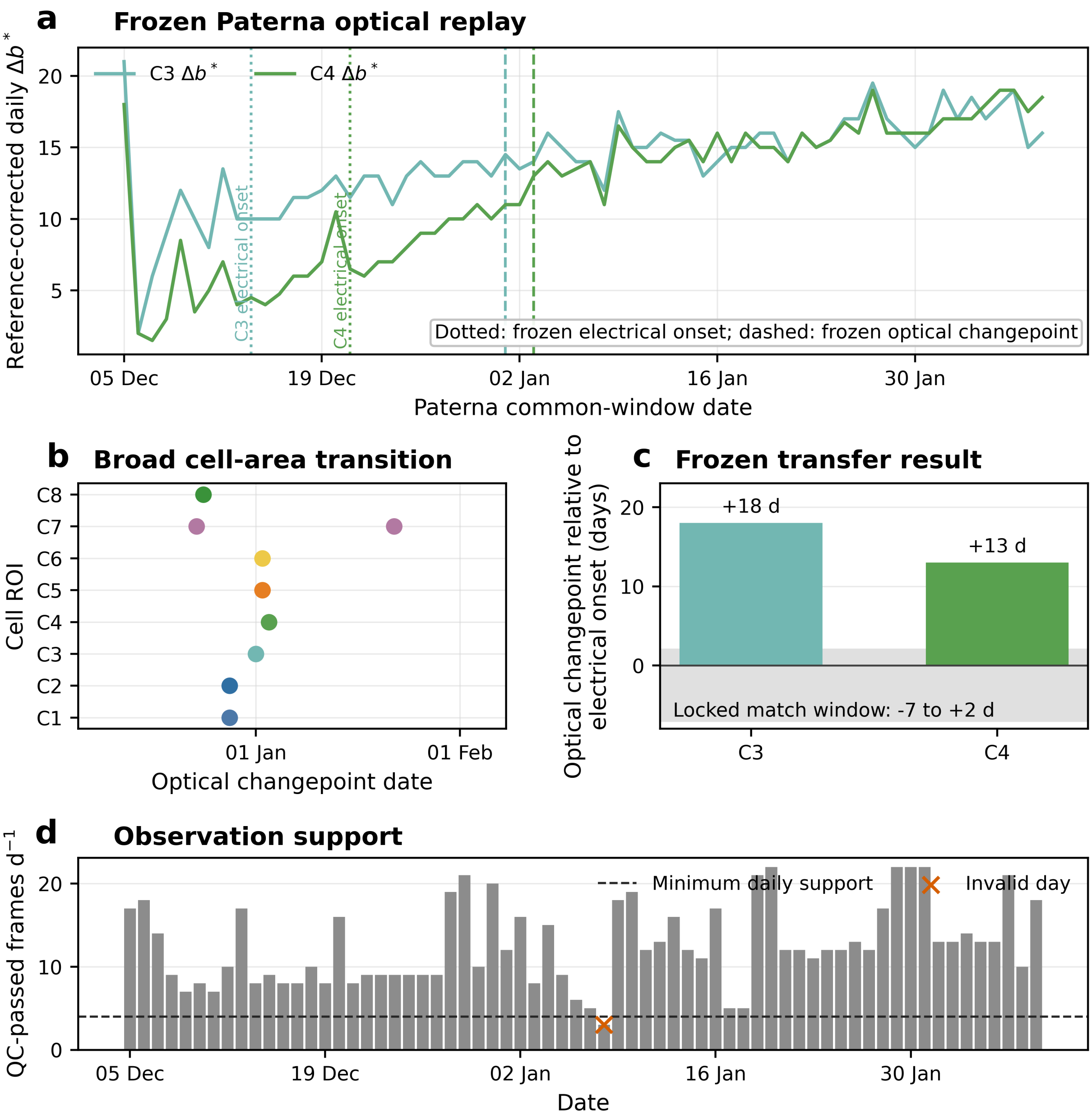

Figure S18. Paterna optical transfer test. a, Daily reference-corrected Δb^* for C3 and C4 with the reproduced formal electrical onsets (dotted) and optical changepoints (dashed). b, Optical changepoints across all eight cell regions, showing a broad late-December/early-January transition rather than a C3/C4-specific precursor. c, C3 and C4 optical changes occur +18 and +13 d after electrical onset, outside the locked −7 to +2 d match window. d, Daily quality-passed image support; 65/66 days meet the four-frame threshold.

Table S17. Post-hoc all-eight Paterna electrical context from the later harmonised 66-day table.

Cell	Numeric days	Non-zero days	Day-1 resp.	First-7 median	66-d median	Target?
C1	66/66	66/66	0.1106	0.008371	1.83×10^{-6}	No
C2	66/66	66/66	0.1064	0.001261	9.66×10^{-5}	No
C3	66/66	66/66	0.07839	0.1075	0.004371	Yes
C4	66/66	66/66	0.1064	0.1490	0.02886	Yes
C5	66/66	66/66	0.1205	0.001076	1.99×10^{-4}	No
C6	66/66	58/66	0.1199	0.001689	3.56×10^{-7}	No
C7	66/66	66/66	0.1087	0.005536	4.25×10^{-4}	No
C8	66/66	66/66	0.1227	0.006318	3.89×10^{-4}	No

All eight channels contain numerical electrical records across the harmonised common window. C6 has eight exactly zero energy values and 58/66 non-zero days; the other channels have 66/66 non-zero days. The six non-target trajectories lose signal steeply during the early part of the record, whereas C3 and C4 retain appreciable output longer. This table is descriptive context and does not alter the pre-specified C3/C4 target set.

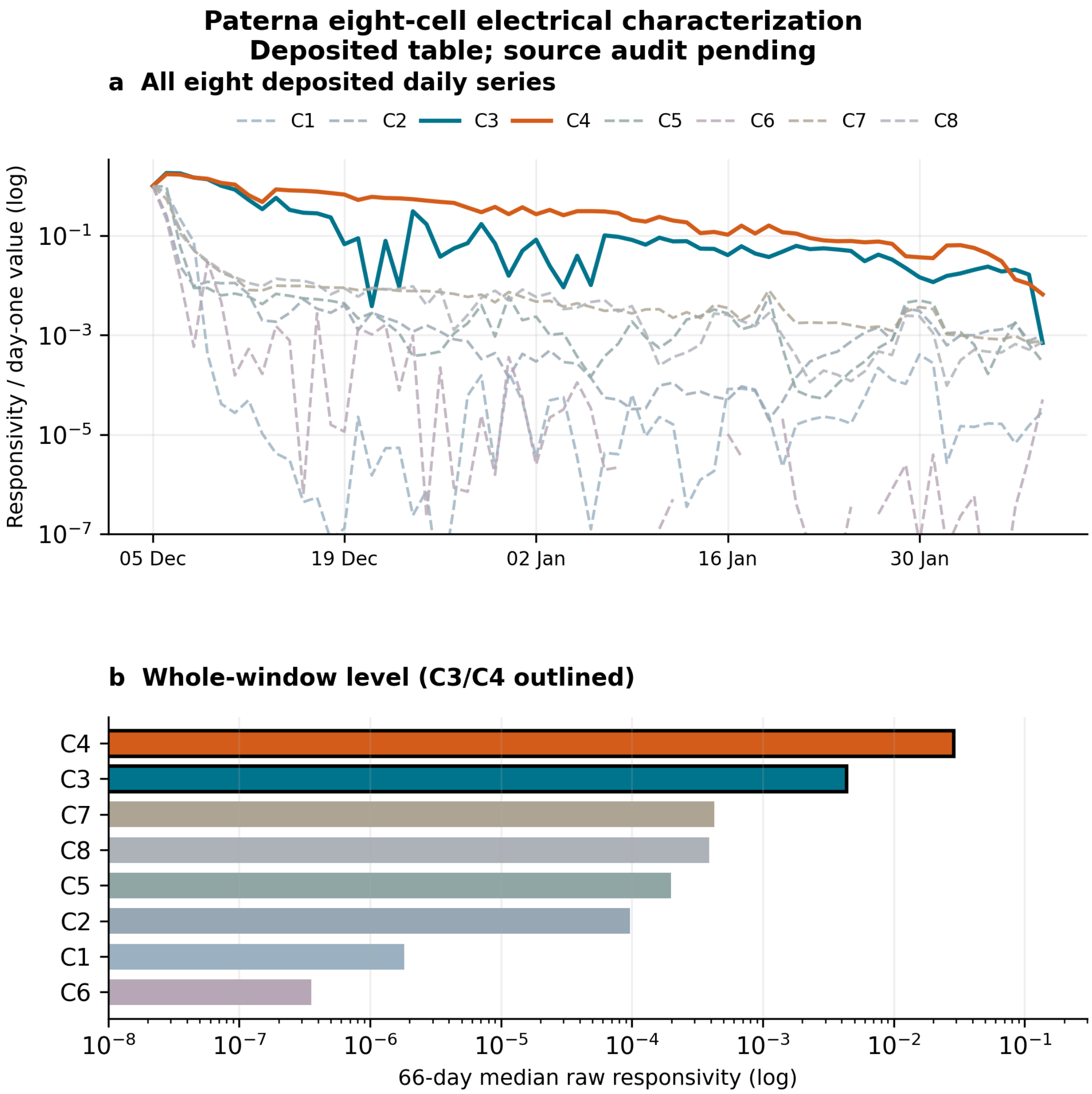

Figure S19. All-eight Paterna electrical context from the harmonised 66-day table. a, Daily responsivity normalised to each channel's 5 December value; measured zeros are omitted only on the logarithmic display. b, Raw whole-window median responsivity and the formal C3/C4 target identities.

S20. Spatial and cross-site common-mode analyses

These exploratory analyses were performed after the frozen Paterna transfer result. They test whether localised spatial information or site-common-mode removal reveals a signal hidden by the cell-median Δb^* metric; they cannot replace or reclassify the primary result. This bounded use of imaging and prediction complements recent outdoor machine-learning and sunlight-excited imaging studies.[3, 6, 12]

For the spatial analysis, 80 quality-passed frames from 5–11 December formed a pixelwise median illumination-corrected Lab baseline. The primary exploratory metrics were the 90th percentile of pixelwise ΔE_{76}, fraction of pixels with $\Delta E_{76} > 5$, spatial b^* interquartile range and 90th percentile of gradient change. C3 ranked highly for some upper-tail and heterogeneity measures before its onset, but this ranking was already present during baseline and did not emerge specifically at the formal 14 December onset. C4 showed no consistent target- specific increase around its formal 21 December onset. The $\Delta E_{76} > 5$ fraction was high for several cell regions during baseline and is therefore an illumination/registration-sensitive change metric rather

than a literal degraded-area fraction.

The common-mode analysis used substrates A–D as the optical experimental units. For the Alpine C5/C6 substrate, C6 had no valid positive electrical baseline and therefore did not contribute to the substrate electrical aggregate; the co-located C5 series defined the electrical value, while the optical substrate mapping was retained. For each site and date, the median across the four substrates defined the optical or electrical common mode; the substrate minus that median defined the residual. The cross-site scalar residual correlation with subsequent electrical loss was 0.055 at the Alpine site and 0.002 at Paterna. Paterna spatial-b^* and gradient residuals were also close to zero ($\rho = -0.069$ and -0.011).

The fair predictive comparison used the same minimal baseline at both sites: current three-day electrical residual, optical common mode and plane-of-array energy. Ridge models were evaluated in ten date-disjoint blocks with a 3-d purge around each held-out block. Adding the scalar optical residual increased mean fold MAE from 0.321 to 0.332 at the Alpine site and from 0.717 to 0.721 at Paterna. Adding the scalar and two Paterna spatial residuals increased mean MAE to 0.737. The scalar model improved 4/10 Alpine folds and 6/10 Paterna folds, but not the mean error; the spatial model improved 3/10 Paterna folds. The continuous residual analysis therefore did not identify a transferable prognostic relation. This does not contradict the Alpine event detector, which was defined by discrete changepoints and evaluated against frozen terminal events rather than by continuous short-horizon regression.

Table S18. Substrate-specific optical residual association with future loss.

Site	Residual feature	Spearman ρ	Substrate-days
Alpine	Scalar optical residual	0.055	252
Paterna	Scalar optical residual	0.002	252
Paterna	Spatial b^* IQR residual	-0.069	252
Paterna	Spatial-gradient p90 residual	-0.011	252

Table S19. Purged blocked prediction.

Site	Model	Mean fold MAE	Mean change versus baseline
Alpine	Shared baseline	0.321	–
Alpine	Baseline + scalar optical residual	0.332	0.011 worse
Paterna	Shared baseline	0.717	–
Paterna	Baseline + scalar optical residual	0.721	0.004 worse
Paterna	Baseline + scalar and spatial residuals	0.737	0.020 worse

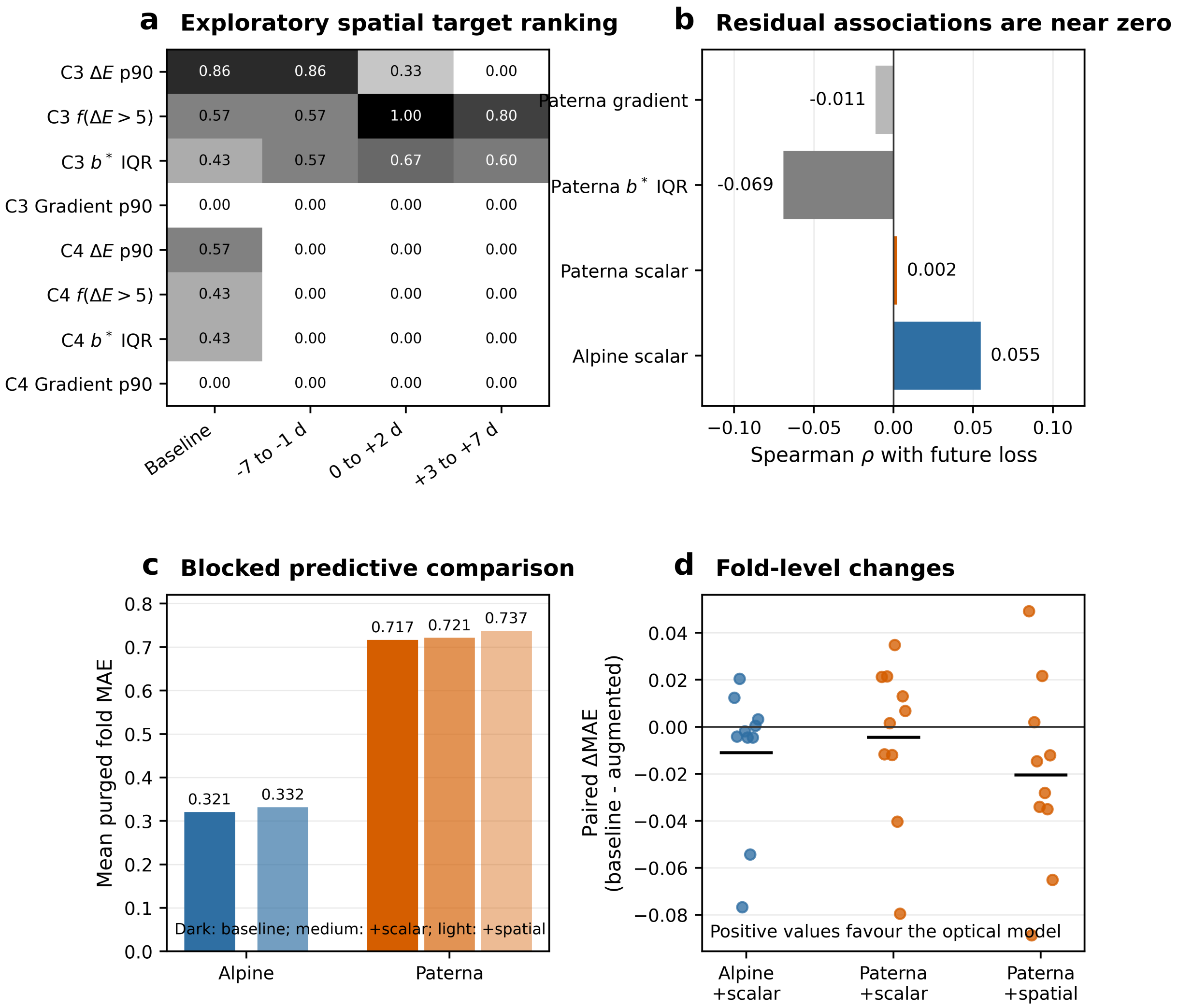

Figure S20. Spatial and cross-site common-mode analyses. a, Fraction of days on which each target cell ranked among the two highest of eight cell regions for four exploratory spatial features; C3 elevation is not onset-specific and C4 shows no onset-specific pattern. b, Substrate-specific residual correlations with subsequent electrical loss are near zero. c, Mean MAE for the shared baseline and optical-residual models in ten purged date blocks. d, Paired fold changes; positive values favour the optical model, whereas mean changes are negative for all three augmented models.

S21. Descriptive regime evidence ladder

Primary contrast	Regime	Pre-event percentile	Block-permutation p	Holm p
`C2_STC_primary`	`thermomechanical`	96.8	0.396	0.396
`C8_SHT_primary`	`moisture_temperature`	100.0	0.050	0.099

The mid-winter transition occurred within the campaign's most intense sustained thermal-cycling period, and the warm-season transition within its most humid period. Because the future-lead windows were also elevated, the regimes persisted beyond the transitions and temporal precedence cannot be established. The regime did not become a causal label.

S22. Machine-readable sequence-preserving replay deliverables

Two 30-day Alpine field-derived chamber recipes are maintained as CSV files in the project repository: the C2 pre-terminal sequence and the C8 warm-season LdT sequence. The archived assembler additionally uses the corresponding daily causal table to produce the complete 66-day Paterna matched-window sequence and an environmental-only 30-day warm-humid replay selected by the maximum standardised Tdev_mean + RH_night index. Electrical outcomes are excluded from that selection rule. Because no equivalent RGB camera operated at Paterna, surface state is explicitly marked not instrumented and no Alpine wetness value is imputed across sites. The rebuilt Paterna table contains 66/66 calendar days and passes the frozen physical-QC gate: Tdev_min >= -50 °C, Tdev_max <= 80 °C, RH_night <= 100% in the working physical series and heating/cooling rates within +/-100 °C h-1.

Paterna sensor QC was applied before daily aggregation and before calculation of ramp rates, vapour pressure, dew-point margin and heat-moisture indices. Relative-humidity readings were likewise preserved raw; known sentinels and grossly implausible values were masked, while finite sensor overshoots were flagged and constrained to the physical 0–100% interval only for working calculations and plots. The final matched replay spans 6.0–44.16 °C, RH_night up to 100%, and heating/cooling extrema of 23.68 and -19.20 °C h-1.

ID	Preregistered hypothesis	Comparison	Primary readout
H1 threshold	Damage begins only after cumulative exposure exceeds a predeclared threshold.	Alpine and Mediterranean sequences; dry-cycle and moisture-only controls	Terminal health loss and chromatic changepoint under >=3 independent substrates/condition
H2 damage memory	Delta D = beta1 E + beta2 D_prev * E; prior damage amplifies the same exposure.	Sequence replay with matched cumulative dose but altered order	Order-dependent divergence at equal integrated dose
H3 order effect	Wet/humid priming before rapid cooling produces more damage than the reverse order.	Wet->freeze versus freeze->wet	Interaction contrast fixed before experiment
H4 wet-primed freeze-thaw	Surface wetness increases freeze-thaw damage relative to dry cycling.	Validated wetness/thaw surface state plus dry-cycle control	Current-limited failure phenotype and spatial endpoint
H5 regime persistence control	Persistent seasonal regime alone is insufficient without the observed sequence.	Time-shuffled or phase-shifted replay retaining marginal distributions	No terminal transition under sequence-disrupted control

S23. Archive structure and event-resolved ISOS-O reporting

The accompanying Supporting Data and Code package contains the compact machine-readable record needed to audit the reported analyses. A top-level `README.txt` and `FILES_AND_SCRIPTS.csv` map the principal figures and revised analyses to their source data and scripts. The package includes the historical Paterna unblinding input, the separate harmonised two-site table, the archived Valencia daily power-sum source used for the Paterna per-cell yields, frozen chromatic and terminal manifests, wetness-development and hidden-audit tables, figure-level inputs, raw endpoint-XRD patterns, Paterna optical/spatial and matching

tables, the switching-benchmark ledger, analysis and plotting scripts, `checks/check_key_results.py`, and a complete `SHA256SUMS.txt` manifest. Owing to file size, the complete long-form sensor streams and full image archive are maintained in the project record and are available from the corresponding author upon reasonable request.[1, 2]

Event-resolved ISOS-O element	Minimum reporting element
Survivor-referenced electrical health	Report definition, surviving references, uncertainty and failure of conventional raw T_{80} under reversible modulation.
Terminal-event calendar	Report event dates, objective persistence rule, right censoring and substrate-level experimental unit.
Retrospective optical correspondence	Frozen-marker correspondence, optical-first offset distribution, other changepoints/window-year and illumination-conditioned misses.
Blind environmental labels	Separate development and frozen audit; report block structure, uncertainty intervals and promotion rule.
Observation-process QC	Report usable support, night/day limitation, glare/blur/OOD exclusions and gaps without interpolation.
Predictive utility	Use date-blocked out-of-sample tests and report minimum detectable improvement; distinguish validity from utility.
Endpoint provenance	Map sample to device fate and disclose recovery, storage, delamination and measurement timeline.
Replay deliverables	Publish machine-readable sequence protocols with ramps, order, surface state and bias.

Supplementary references

References

[1] Khenkin, M. V. et al. Consensus statement for stability assessment and reporting for perovskite photovoltaics based on ISOS procedures. Nature Energy 5, 35–49 (2020). doi:10.1038/s41560-019-0529-5.

[2] Jacobsson, T. J. et al. An open-access database and analysis tool for perovskite solar cells based on the FAIR data principles. Nature Energy 7, 107–115 (2022). doi:10.1038/s41560-021-00941-3.

[3] Paraskeva, V. et al. Diurnal changes and machine learning analysis of perovskite modules based on two years of outdoor monitoring. ACS Energy Letters 9, 5081–5091 (2024). doi:10.1021/acsenergylett.4c01943.

[4] Dunfield, S. P. et al. PARASOL: an open-source platform for parallel testing of solar modules. Sustainable Energy & Fuels 9, 4738–4747 (2025). doi:10.1039/D5SE00513B.

[5] Remec, M. et al. Seasonality in perovskite solar cells: insights from 4 years of outdoor operation. Advanced Energy Materials 15, 2501906 (2025). doi:10.1002/aenm.202501906.

[6] Gayot, F. et al. Outdoor implied open-circuit voltage imaging of perovskite solar cells using sunlight excitation. Joule 9, 101946 (2025). doi:10.1016/j.joule.2025.101946.

[7] Sánchez, S. et al. Flash infrared annealing for antisolvent-free highly efficient perovskite solar cells. Advanced Energy Materials 8, 1702915 (2018). doi:10.1002/aenm.201702915.

[8] Sánchez, S. et al. Flash infrared pulse-time control of perovskite crystal nucleation and growth from solution. Crystal Growth & Design 20, 670–679 (2020). doi:10.1021/acs.cgd.9b01083.

[9] Sánchez, S., Pfeifer, L., Vlachopoulos, N. & Hagfeldt, A. Rapid hybrid perovskite film crystallization from solution. Chemical Society Reviews 50, 7108–7131 (2021). doi:10.1039/D0CS01272F.

[10] Zhang, Z. et al. Big-data-driven perovskite solar cell stability analysis. Nature Communications 13, 7639 (2022). doi:10.1038/s41467-022-35400-4.

[11] EPFL Laboratory for Molecular Engineering of Optoelectronic Nanomaterials. Outdoor operational stability of perovskite solar cells. EPFL LIMNO project page (accessed 23 July 2026).

[12] Kouroudis, I. et al. Artificial intelligence-based, wavelet-aided prediction of long-term outdoor performance of perovskite solar cells. ACS Energy Letters 9, 1581–1586 (2024). doi:10.1021/acsenergylett.4c00328.
